# Tumor-reactive TCRs within exhausted TILs reveal cancer type-specific immune landscapes in renal cell carcinoma

**DOI:** 10.3389/fimmu.2026.1729388

**Published:** 2026-01-29

**Authors:** Mitsuru Komahashi, Shun Horaguchi, Kayoko Tsuji, Daisuke Hoshino, Takeshi Kishida, Kimitsugu Usui, Noboru Nakaigawa, Shinya Sato, Hiroshi Hamana, Hiroyuki Kishi, Feifei Wei, Yasunobu Mano, Taku Kouro, Shuichiro Uehara, Tetsuro Sasada

**Affiliations:** 1 Division of Cancer Immunotherapy, Kanagawa Cancer Center Research Institute, Yokohama, Japan; 2 Cancer Vaccine and Immunotherapy Center, Kanagawa Cancer Center Research Institute, Yokohama, Japan; 3 Department of Pediatric Surgery, Nihon University School of Medicine, Tokyo, Japan; 4 Cancer Biology Division, Kanagawa Cancer Center Research Institute, Yokohama, Japan; 5 Department of Urology, Kanagawa Cancer Center, Yokohama, Japan; 6 Molecular Pathology and Genetics Division, Kanagawa Cancer Center Research Institute, Yokohama, Japan; 7 Morphological Information Laboratory, Kanagawa Cancer Center Research Institute, Yokohama, Japan; 8 Department of Immunology, Faculty of Medicine, Academic Assembly, University of Toyama, Toyama, Japan

**Keywords:** clear cell renal cell carcinoma, machine-learning, organoid, single-cell RNA sequencing, T cell receptor (TCR), TCR-T cell therapy, transcriptome, tumor-infiltrating lymphocytes

## Abstract

Clear cell renal cell carcinoma (ccRCC) presents a unique immunological paradox: abundant CD8^+^ tumor-infiltrating lymphocytes (TILs) correlate with poor prognosis. To clarify their functional status and therapeutic potential, we performed single-cell transcriptomic profiling of TILs from 15 patients with ccRCC and functionally validated dominant T cell receptor (TCR) clonotypes using autologous tumor-derived organoids. Single-cell RNA sequencing revealed dynamic shifts in T cell composition, with effector and progenitor-exhausted CD8^+^ T cells declining and terminally exhausted CD8^+^ and regulatory CD4^+^ T cells enriched in advanced tumors. Despite this exhausted phenotype, in an exploratory analysis with five patients, approximately half of the top 20 TCR clonotypes retained anti-tumor reactivity when re-expressed in non-exhausted T cells, as evidenced by TCR-T cell-mediated cytotoxicity and IFN-γ production against autologous organoids. Transcriptomic signatures enabled the development of a penalized logistic regression classifier that distinguished tumor-reactive from bystander T cells with high accuracy, with AUCs of 0.903 (training) and 0.913 (test). Cross-cancer comparison with pancreatic ductal adenocarcinoma (PDAC) datasets revealed limited generalizability, highlighting the need for cancer type-specific models. Notably, ccRCC-specific TILs exhibited mature, functionally differentiated profiles with limited proliferation, consistent with chronic antigen exposure, whereas PDAC-reactive TILs showed highly proliferative and activated phenotypes indicative of ongoing clonal expansion. Collectively, these findings suggest key features of the immune landscape in ccRCC and provide a preliminary, proof-of-concept transcriptomic framework for prioritizing candidate tumor-reactive TCRs. These insights suggest the feasibility of identifying candidate TCRs for future development of TCR-based adoptive T cell therapies in ccRCC and emphasize the importance of integrating single-cell profiling with functional analyses to refine immunotherapeutic strategies. Given the limited sample size, our results should be considered exploratory and hypothesis-generating, and future studies will be required to validate these findings in larger, independent ccRCC cohorts.

## 1 Introduction

Renal cell carcinoma (RCC) represents the 14th most commonly diagnosed cancer worldwide, and clear cell renal cell carcinoma (ccRCC) is the most prevalent histological subtype, accounting for approximately 80% of all RCC cases (1). Patients with localized ccRCC are typically managed with surgical excision, but those with metastatic disease require systemic therapy. Recently, the advent of immune checkpoint inhibitors (ICIs) has revolutionized the treatment landscape of metastatic ccRCC, significantly improving overall survival (1, 2). However, many patients with metastatic ccRCC fail to achieve durable responses to ICIs, underscoring the need for additional immunotherapeutic strategies. Among these, T cell receptor-engineered T (TCR-T) cell therapy, which allows for the redirection of T cells to recognize and kill tumor cells, has emerged as a promising approach for solid tumors (3–5). Although this approach has shown efficacy in several solid cancers, its application in ccRCC remains limited (6–8). In particular, reliable methods to accurately identify tumor-specific TCRs have yet to be fully developed.

Tumor-infiltrating lymphocytes (TILs) are known to contain T cells capable of recognizing tumor antigens. However, accurately identifying truly tumor-reactive T cells among the broader TIL population in RCC remains a significant challenge (9, 10). Additionally, RCC displays a unique immunological paradox: a high proportion of CD8^+^ T cells in the tumor microenvironment is typically associated with improved prognosis in most solid tumors, but with poorer clinical outcomes in RCC (11–14). The mechanisms underlying this counterintuitive observation, however, remain incompletely understood. To address these knowledge gaps, we conducted single-cell RNA sequencing (scRNA-seq) analysis of TILs from patients with ccRCC to characterize the phenotypic and transcriptional landscape. In addition, we functionally validated T cell clonotypes by assessing their tumor reactivity through co-culture assays with autologous ccRCC-derived organoids. Through this integrative approach, our goal was to gain deeper insight into the relationship between T cell phenotypes and tumor reactivity in ccRCC, and to evaluate the potential of transcriptional features for predicting tumor-specific TCRs, which may ultimately guide the clinical application of TCR-T cell therapy in ccRCC.

## 2 Materials and methods

### 2.1 Patients

A total of 15 patients with pathologically confirmed ccRCC who underwent surgery at Kanagawa Cancer Center (Yokohama, Japan) between February 2022 and September 2024 were enrolled in this study. The study was conducted in accordance with the principles of the Declaration of Helsinki and approved by the Institutional Review Board of Kanagawa Cancer Center (approval number: 2017-11). Written informed consent was obtained from all participants prior to enrollment, following an explanation of the study’s objectives and implications.

### 2.2 Single-cell RNA sequencing and Immunoprofiling

Resected ccRCC tumor tissues obtained during surgery were minced into small pieces and enzymatically dissociated into single-cell suspensions using the Human Tumor Dissociation Kit together with the GentleMACS Dissociator (Miltenyi Biotec, Bergisch Gladbach, Germany) for 1 hour at 37°C. The resulting single-cell suspensions were stained with FITC anti-CD3ϵ monoclonal antibody (mAb) (BioLegend 300406, SanDiego, CA) and CD3^+^ T cells were isolated using FACSAriaII cell sorter (BD Bioscience, San Jose, CA).

Purified T cells were used to generate single-cell sequencing libraries using the Chromium Controller and Chromium Next GEM Single Cell 5’ Reagent Kits v2 (10x Genomics, Pleasanton, CA), according to the manufacturer’s instructions. Barcoded and amplified cDNAs were divided into two libraries: one for TCR profiling and the other for gene expression analysis. Libraries were sequenced using a NovaSeq X sequencer (Illumina, San Diego, CA) and FASTQ files were processed with the Cell Ranger pipeline (10x Genomics).

Downstream analyses were performed using Seurat R package (v.5.3.0) (15). First, gene expression data from individual patients were integrated with the IntegrateLayers function. TCR information was mapped to each cell based on shared barcodes. After normalization, scaling and clustering, clusters were manually annotated based on known marker gene expression (**Supplementary Table S1**). The number of events for each annotated cell type was quantified by decoding sample IDs. For TCR-T cell generation, nucleotide sequences of the top 20 most abundant TCR clonotypes were extracted from the consensus_annotations.csv and filtered_contig.fasta files generated by Cell Ranger. The clonal diversity estimated by Shannon index of CD8^+^ T cells in each sample was calculated using immunarch 0.9.1 R package (v.0.9.1; https://immunarch.com). Analysis code is available in the **Supplementary Text 1**.

### 2.3 Bulk RNA sequencing and cell type deconvolution

Resected ccRCC tumor tissues were stored overnight at 4°C in RNAlater reagent (Thermo Fisher Scientific, Waltham, MA) and total RNA was isolated using the RNeasy Plus Mini kit (Qiagen, Venlo, Netherlands). Sequencing libraries were prepared using the SMART-Seq Standard kit (Takara-Bio, Shiga, Japan) and sequenced using a NovaSeq X sequencer (Illumina). FASTQ files were trimmed with fastp (16) and mapped to the GRCh38 human reference genome using STAR (17). Gene expression was quantified using RSEM (18), and transcripts per million (TPM) values were used as input for CIBERSORTx (https://cibersortx.stanford.edu). For this analysis, a custom signature matrix was created from publicly available ccRCC scRNA-seq dataset (GSE207493). Two samples each from stage I, stage II and stage III tumors were selected, normalized, combined, batch-corrected, and clustered using Seurat (v5.3.0) (15). Clusters were annotated to cell types based on the expression of known marker genes (**Supplementary Table S2**). The resulting gene expression matrix with cell type annotations was downsampled to 50 cells per cell type and used as the CIBERSORTx signature matrix. Analysis code is available in the **Supplementary Text 2**.

### 2.4 Construction of TCR expression vectors and generation of TCR-T cells

TCRα and β variable region cDNA sequence of top 20 abundant clonotypes were obtained from consensus_annotations.csv files of CellRanger output. TCR expression vectors were constructed by cloning artificially synthesized Vα and Vβ cDNAs (Eurofins Genomics KK, Tokyo, Japan) into pMXs-IRES-EGFP retrovirus vector containing human TCRα and β constant regions (19) using the Gibson Assembly Mastermix (New England Biolabs, Ipswich, MA) (**Supplementary Figure S1**, **Supplementary Table S3**). The resulting constructs were transfected into the Phoenix AMPHO packaging cell line (ATCC CRL-3213, Manassas, VA) with the Xfect transfection reagent (Takara-Bio) to produce virus supernatants. For transduction, 500 µL of viral supernatants was added to RetroNectin-coated (40 µg/ml; Takara-Bio) 24-well plates and centrifuged at 2000 × g for 2 hours at 32°C. After washing with PBS supplemented with 0.5% fatty acid–free bovine serum albumin (Fujifilm Wako, Osaka, Japan), 1.0 × 10^5^ Jurkat Δαβ CD8a cells (20) were seeded into each well and centrifuged at 500 × g for 10 minutes at 37°C. To evaluate TCR expression, cells were cultured at 37°C for 4 days and then stained with PE-anti-CD3ϵ mAb (BioLegend 300408), followed by analysis using a FACSCantoII flow cytomter (BD Biosciences).

Peripheral blood mononuclear cells (PBMCs) were isolated from healthy donors after obtaining written informed consent, using Ficoll-paque (GE healthcare, Chicago, IL) density gradient centrifugation. To generate TCR-T cells, 1 × 10^6^ PBMCs were seeded into 24-well plates pre-coated with anti-human CD3ϵ mAb (40 µg/ml; BioLegend 300402) and cultured in AIM-V medium (Thermo Fisher) supplemented with 10% human AB serum (MP Biomedicals, Irvine, CA) and 100 U/ml human IL-2 (Peprotech, Rocky Hill, NJ) at 37°C for 2 days. Activated PBMCs (2.5 × 10^6^) were then transferred to virus-coated plate as described above and centrifuged at 500 × g for 10 minutes at 37°C. After 24 hours, a second round of viral infection was performed using the same procedure. Three days after the 2nd infection, TCR gene transfer efficiency was confirmed by detecting EGFP-positive cells via flow cytometry.

### 2.5 Establishment of organoids from tumor tissues

Resected ccRCC tumor tissues were mechanically minced and enzymatically digested with Dispase II (2 U/ml; Merck, Darmstadt, Germany) and collagenase (0.5 mg/ml; Merck) at 37°C for 30 minutes. The resulting cell suspension was passed through a 70 µm cell strainer, and the small clumps were treated with ACK Lysing Buffer (Thermo Fisher) to remove red blood cells. These clumps were then seeded onto Matrigel-coated plates (Matrigel Matrix Basement Membrane, Growth Factor Reduced; Corning, NY). Larger clumps that remained on the strainer were further digested using TrypLE Express (Thermo Fisher) and filtered again through a 70-µm strainer. The resulting smaller clumps were also seeded onto Matrigel-coated plates and cultured in organoid medium consisting of DMEM/F-12 (Thermo Fisher) supplemented with the following components: 10% FGF10-conditioned medium, 500 ng/ml recombinant R-spondin 1 (prepared in-house using an E. coli expression system), 10 ng/ml human recombinant EGF (Thermo Fisher), 10 µM Y-27632 (Fujifilm-Wako), 1.25 mM N-Acetyl-L-cysteine (Merck), 1.5% B-27 Supplement (50×, serum-free; Thermo Fisher), 5 µM A-83-01 (Fujifilm-Wako), 2 mM GlutaMAX Supplement (Thermo Fisher), and 10 mM HEPES (Thermo Fisher). Organoids were passaged upon reaching confluency by dissociating the Matrigel with 2 U/ml Dispase II, fragmenting the organoids into small clumps with TrypLE Express. The resulting clumps were re-embedded in fresh Matrigel and cultured in organoid medium, which was refreshed weekly.

### 2.6 Immunohistochemical analysis

Original tumor tissues and derived organoids were fixed in 10% formalin and embedded in paraffin. Four-µm-thick sections were cut from formalin-fixed, paraffin-embedded tissues, deparaffinized, and stained with hematoxylin and eosin according to standard protocols. For immunohistochemistry, sections underwent heat-induced epitope retrieval, followed by blocking with 3% hydrogen peroxide. The sections were then incubated with the following primary antibodies: anti-carbonic anhydrase 9 (CA9) mAb (Abcam ab108351, Cambridge, United Kingdom), anti-CD10 mAb (Abcam ab227640), anti-PAX8 mAb (Nichirei 418211, Tokyo, Japan), and anti-Ki-67 mAb (Nichirei 418071). After washing, slides were incubated with either HRP-conjugated anti-mouse Fab’ antibody (Nichirei 424131) or HRP-conjugated anti-rabbit Fab’ antibody (Nichirei 424141) for 30 minutes at room temperature. Signal detection was performed using 3,3′-diaminobenzidine tetrahydrochloride (Nichirei 725191), and the sections were counterstained with hematoxylin.

### 2.7 *In vitro* LDH release and IFN-γ secretion assays

TCR-T cells and autologous organoids were co-cultured in triplicate at effector-to-target (E:T) ratios of 2.5:1, 5:1, and 10:1 in 384-well plates. Cultures were maintained overnight at 37°C in AIM-V medium supplemented with 10% human AB serum and 100 U/ml IL-2. Following co-culture, supernatants were collected for analysis of LDH activity and IFN-γ secretion. LDH activity was measured using the Cytotoxicity LDH Assay Kit-WST (Dojindo, Kumamoto, Japan) according to the manufacturer’s instruction. Specific LDH release was calculated relative to the maximum LDH release induced by lysis buffer. IFN-γ concentrations were quantified after 1:5 dilution of supernatants using the BD OptEIA™ Human IFN-γ ELISA Set (BD Bioscience), following the manufacturer’s protocol. PBMCs transduced with EGFP-containing expression vectors without TCR constructs served as controls. Reactive TCRs were defined based on these *in vitro* assay results. Reactivity was assigned when, at an E:T ratio of 10:1, either the LDH release assay or the IFN-γ ELISA showed a statistically significant difference from the control sample, and when at least two data points showed statistically significant differences across all tested conditions.

### 2.8 Feature selection and classification using signature scores and machine-learning

Tumor-reactive CD8^+^ T cells were identified by *in vitro* LDH release and IFN-γ secretion assays. To predict bystander T cell clonotypes, TCRβ CDR3 amino acid sequences from our dataset were analyzed using the TCR-Match Python script against the IEDB database (http://tools.iedb.org/tcrmatch). T cells carrying TRB CDR3 sequences identical to previously reported TCRs that recognize epitopes derived from EBV, CMV, or influenza virus were labeled as bystander T cells.

Tumor-reactivity–related gene signature scoring was performed using a predefined gene set (TR Score; 30 genes: TNFRSF9, VCAM1, TIGIT, HAVCR2, GZMB, ACP5, NKG7, KRT86, LAYN, HLA-DRB5, CTLA4, HLA-DRB1, IGFLR1, HLA-DRA, LAG3, GEM, CXCL13, LYST, GAPDH, CD74, HMOX1, HLA-DPA1, DUSP4, CD27, ENTPD1, AC243829.4, HLA-DPB1, GZMH, KIR2DL4, and CARD16) (21). Per-cell signature enrichment was calculated with the UCell algorithm using AddModuleScore_UCell (Seurat-compatible implementation), and the resulting TR Score was added to the Seurat object as cell-level metadata for downstream analyses and visualization.

CD8^+^ T cells were randomly divided into training (80%) and test (20%) sets using the caret::createDataPartition (22). Differentially expressed genes (DEGs) between tumor-reactive and bystander CD8^+^ T cells were computed in the training set using Seurat::FindMarkers function (15), with the criteria of adjusted *p* < 0.05, nominal *p* < 0.05, and |log_2_FC| > 1, excluding TCR genes (TRAV and TRBV families). Random forest (RF), penalized logistic regression (PLR), support vector machines (SVM), and gradient boosting machines (GBM) classifiers were trained using the caret::train function with 5-fold cross-validation, with hyperparameter tuning of the mtry value ranging from 1 to 10 (22). Model performance was evaluated based on area under the receiver operating characteristic (ROC) curve (AUC). The final model performance was evaluated using out-of-fold predictions from the 5-fold cross-validation on the training set, and on the held-out test set, summarized by ROC curves. AUC values were calculated using the pROC package (v1.18.5) (23). Analysis code is available in the **Supplementary Text 1**.

### 2.9 ROC analysis for tumor-size-associated T cell subset changes

For each T cell subset, samples were dichotomized into high- and low-frequency groups based on the median value across the cohort. ROC curves were generated using tumor diameter as the predictor and the binary subset status as the outcome. The direction of the ROC analysis was specified according to the observed correlation between tumor size and subset frequency (positive or negative). The optimal cutoff was defined as the value that maximized the Youden index.

To assess the stability of the estimated cutoff values in this relatively small cohort, bootstrap resampling was performed with 2000 iterations. In each bootstrap sample, ROC analysis and cutoff estimation were repeated, and the distribution of optimal cutoffs was summarized by the median and the 95% confidence interval. ROC-based cutoff values were used solely to assess the consistency of tumor size-associated patterns and were not used to define groups in the primary analyses. The R script for these calculations is shown in **Supplementary Text 3**.

### 2.10 Statistical analysis

Statistical analyses were performed using the R packages pROC (v1.18.5) (23) and Seurat (v5.3.0) (15). An unpaired Student’s t test was used to compare means between two groups unless otherwise specified. All *p* values were two-sided, with *p* < 0.05 considered statistically significant. Correlations between tumor diameter and either cell proportions or Shannon index were assessed using Pearson’s product–moment correlation coefficient, implemented with the R packages ggplot2 (https://ggplot2.tidyverse.org) and ggpubr (https://rpkgs.datanovia.com/ggpubr).

## 3 Results

### 3.1 Characterization of tumor-infiltrating T cells by scRNA-seq in ccRCC

**Figure 1:**
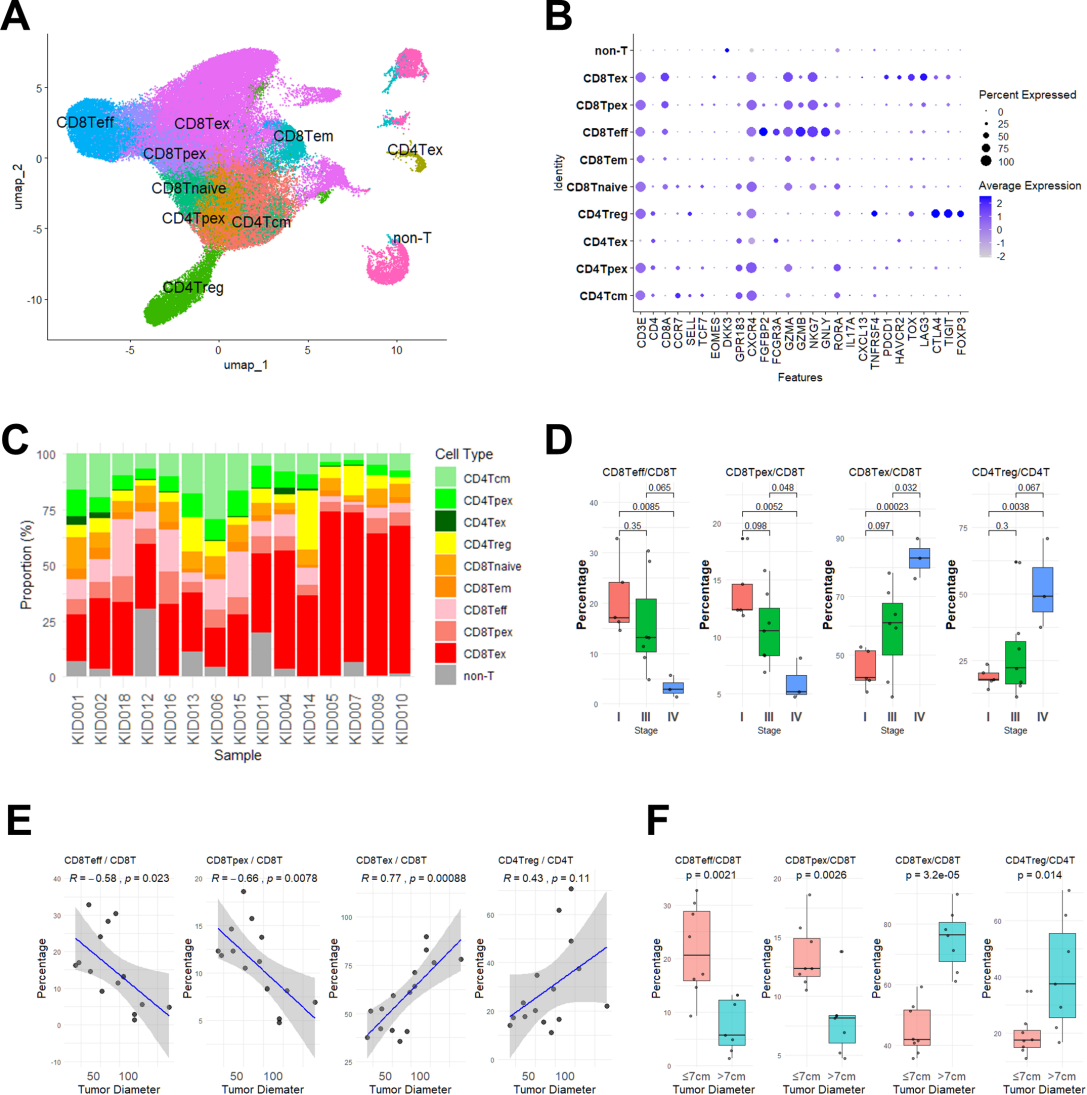
Characterization of tumor-infiltrating T cells by single-cell RNA sequencing in ccRCC. **(A)** scRNA-seq data from CD3^+^ T cells isolated from 15 ccRCC tumor samples were merged, batch-corrected, and visualized using UMAP. Clusters were annotated according to gene expression patterns defined in **Supplementary Table S1**. **(B)** Ballon plot showing the average expression levels and detection frequencies of selected genes in each annotated cluster from **(A)**. **(C)** Proportions of T cell subsets in each sample, arranged in ascending order of tumor diameter. **(D)** Within CD8^+^ T cells, the proportions of CD8Teff, CD8Tpex, and CD8Tex cells, and within CD4^+^ T cells, the proportion of CD4Treg cells, were calculated and compared across tumor stages. *P* values were determined by the Student’s t test. **(E)** Correlations between tumor diameter and proportions of each T cell subset (CD8Teff, CD8Tpex, CD8Tex, and CD4Treg). Linear regression lines (blue) with 95% confidence intervals (gray shaded area) are shown. Pearson correlation coefficients (*R*) and corresponding *p* values are indicated. **(F)** Proportions of T cell subsets (CD8Teff, CD8Tpex, CD8Tex, and CD4Treg) were compared between small (≤ 7cm, n = 8) and large (>7cm, n = 7) tumors. *P* values were determined by the Student’s t test.

Clinical characteristics of 15 ccRCC patients enrolled in this study are shown in **Supplementary Table S4**. According to the AJCC classification, five patients had stage I disease, seven had stage III, and three had stage IV. To characterize phenotypic landscape of TILs, we performed scRNA-seq on CD3^+^ TILs isolated from 15 patients and identified 35 clusters by using UMAP. As shown in **Figure 1A**, these clusters were manually annotated into nine distinct T cell subsets according to the definition listed in **Supplementary Table S1**, **Figure 1B**. **Figure 1C** shows the proportions of each T cell subset.

Notably, within the CD8^+^ T cell compartment, stage IV tumors exhibited a significant decrease in both effector (CD8Teff) and progenitor exhausted T cells (CD8Tpex), along with a marked increase in terminally exhausted T cells (CD8Tex), compared to stage I tumors (**Figure 1D**). In addition, the proportion of regulatory T cells (CD4Treg) within the CD4^+^ T cell compartment was significantly higher in stage IV samples (**Figure 1D**). Because stage III tumors exhibited substantial variation in T cell subset frequencies, no significant differences were observed between stage III and either stage I or stage IV, except for CD8Tpex and CD8Tex, which differed significantly between stage III and stage IV.

We next examined correlations between tumor diameter and the frequencies of individual T cell subsets. As our primary approach, we examined tumor diameter as a continuous variable. As shown in **Figure 1E**, the frequency of CD8Tex cells was strongly and positively correlated with tumor diameter (R = 0.77, *p* = 0.00088), whereas CD8Teff (R = –0.58, *p* = 0.023) and CD8Tpex cells (R = –0.66, *p* = 0.0078) showed significant negative correlations. In addition, CD4Treg cells showed a weak but non-significant positive correlation with tumor size (R = 0.43, *p* = 0.11).

Given the substantial heterogeneity observed within stage III tumors, which are defined by extrarenal invasion rather than tumor size alone, we additionally conducted a secondary stratified analysis using a 7 cm tumor diameter cutoff. This cutoff value has been used in prior prognostic studies of pT3aN0M0 ccRCC to refine risk stratification (24–26). Consistent with this threshold, ROC analyses in our dataset suggested an optimal cutoff of 7.15 cm for CD8Tex-high and CD8Teff-low status, with high discriminative performance (AUCs 0.911 and 0.920, respectively), whereas CD8Tpex-low and CD4Treg-high status showed broader and less stable cutoff ranges (**Supplementary Table S5**). As shown in **Figure 1F**, the proportion of CD8Tex cells was significantly higher in the large group (*p* = 0.000032), while the proportions of CD8Teff and CD8Tpex cells were significantly lower (*p* = 0.0021 and *p* = 0.0026, respectively). Furthermore, the proportion of CD4Treg cells was significantly increased in the larger tumors (*p* = 0.014). Taken together, these results suggest that advanced tumor stage and/or larger tumor size are associated with the accumulation of CD8Tex and CD4Treg cells within the tumor microenvironment.

### 3.2 Characterization of immunological phenotypes in ccRCC by bulk RNA-seq

**Figure 2:**
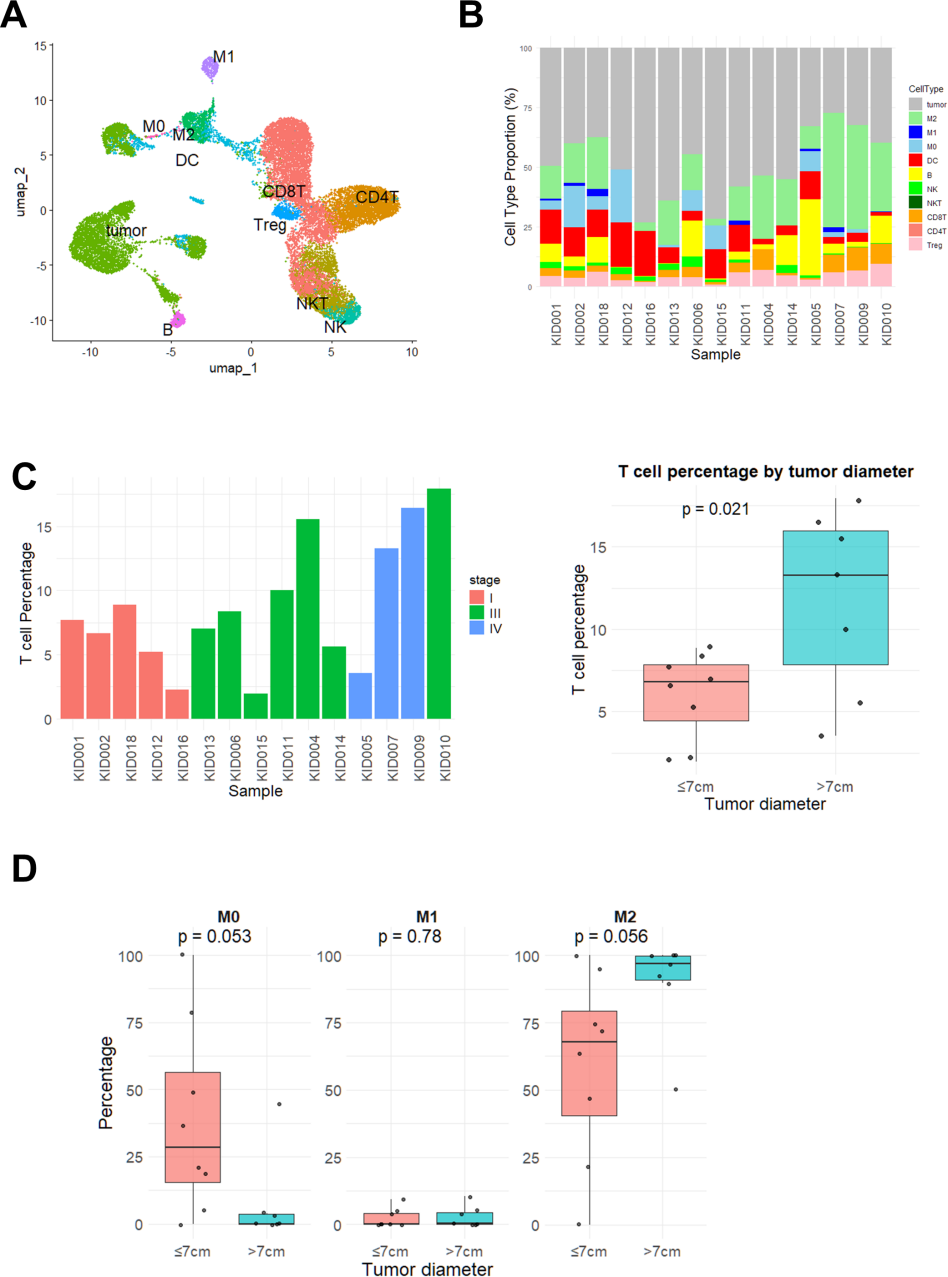
Characterization of immunological phenotypes by bulk RNA sequencing in ccRCC. **(A)** Publicly available scRNA-seq data were analyzed and annotated into 10 immune cell types to generate a reference expression matrix for CIBERSORTx analysis of bulk RNA-seq data. **(B)** Results of CIBERSORTx analysis for 15 ccRCC samples. Samples are arranged in ascending order of tumor diameter. **(C)** Predicted T cell abundance for each sample was calculated by summing the estimated proportions of CD4^+^ and CD8^+^ T cell subsets derived from the CIBERSORTx deconvolution (left). The proportion of T cells was compared between small (≤ 7cm, n = 8) and large (>7cm, n = 7) tumors (right). *P* value was determined using the Student’s t test. **(D)** Within the macrophage compartment, the proportions of M0, M1, and M2 macrophages estimated by CIBERSORTx were compared between small (≤ 7cm, n = 8) and large (>7cm, n = 7) tumors. *P* values were determined using the Student’s t test.

We further performed bulk RNA-seq of tumor tissues from 15 patients to characterize the immunological phenotypes in ccRCC. To estimate the composition of immune cell types, we applied the CIBERSORTx algorithm, which allows deconvolution using a customized reference expression matrix derived from scRNA-seq data. For this purpose, we constructed an RCC-specific signature matrix by manually annotating publicly available scRNA-seq data of RCC tissues (GSE207493) into 10 distinct immune cell types (**Supplementary Table S2**), as illustrated in **Figure 2A**. 

Using this customized signature matrix, we deconvoluted the bulk RNA-seq data from our ccRCC samples and visualized the estimated proportions of each immune cell type (**Figure 2B**). As shown in **Figure 2C**, the proportion of T cells was significantly higher in tumors larger than 7 cm compared to those 7 cm or smaller (*p* = 0.021). In addition, as shown in **Figure 2D**, the proportion of M2 macrophages within the total macrophage compartment tended to be higher in the large tumor group (*p* = 0.056), whereas the proportion of M0 macrophages tended to be lower (*p* = 0.053).

### 3.3 Exhausted phenotype of expanded TIL clonotypes in advanced ccRCC

**Figure 3:**
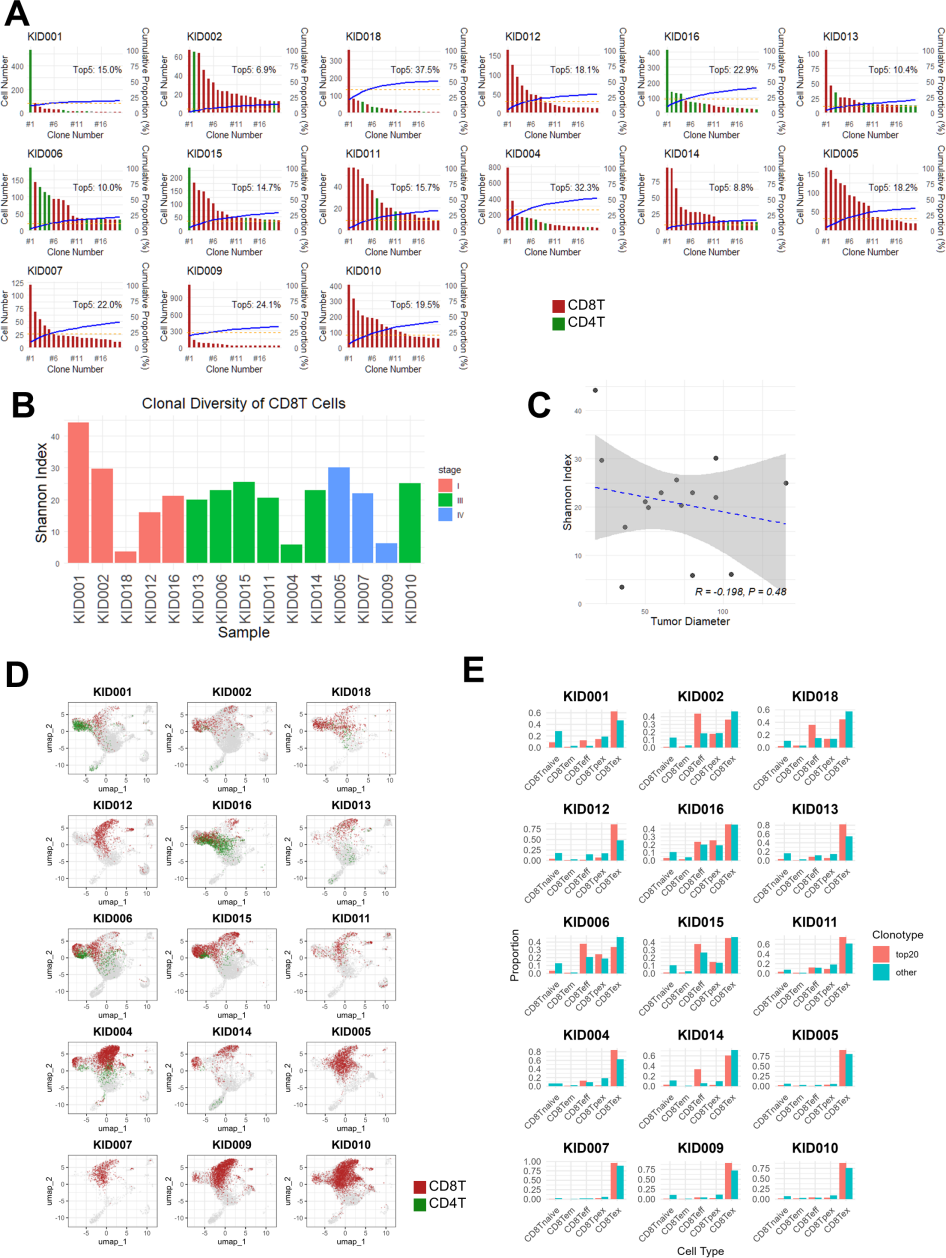
Phenotypes of expanded TIL clonotypes in ccRCC**. (A)** Cell numbers (bar plots: CD4^+^ T cells, green; CD8^+^ T cells, red) and cumulative proportions (blue lines) of the top 20 most abundant TCR clonotypes in each sample. Cumulative proportions (%) of the top 5 clonotypes are also indicated (Yellow dotted line and value (%)). Samples are arranged in ascending order of tumor diameter. **(B)** Bar plots showing TCR diversity (Shannon index) of CD8^+^ T cells in each sample, arranged in ascending order of tumor diameter. **(C)** Correlation between tumor diameter and TCR diversity (Shannon index) across all samples (n=15). Linear regression lines (blue dotted) with 95% confidence intervals (gray shaded areas) are shown. Pearson correlation coefficients (*R*) and corresponding *p* values are indicated. **(D)** T cells corresponding to the top 20 most abundant TCR clonotypes (CD4^+^ T cells, green; CD8^+^ T cells, red) were projected onto the UMAP plot. **(E)** Distribution of CD8^+^ T cell subsets within the top 20 clonotypes (red bars) compared with the remaining CD8^+^ T cells (blue bars).

We analyzed the clonal expansion patterns of T cells in each tumor sample using scRNA-seq data. As shown in **Figure 3A**, in 13 of the 15 cases, the top five most abundant TCR clonotypes accounted for more than 10% of the total TIL population, suggesting strong clonal selection and expansion. Interestingly, larger tumors (KID005/007/009/010) did not contain CD4^+^ T cells among the top 20 most abundant TCR clonotypes, whereas most smaller tumors showed CD4^+^ T cells at higher clonal frequencies. Notably, the degree of clonal diversity estimated by the Shannon index of TCR clonotypes within CD8^+^ T cell subsets varied considerably among samples, reflecting heterogeneity in T cell responses across tumors (**Figure 3B**). As shown in **Figure 3C**, no significant association was observed between clonal diversity assessed by Shannon index and tumor diameter. In addition, statistical comparisons across stages and between tumor size groups showed no significant differences in the occupancy of top 20 clonotypes, suggesting no association between tumor stages or size and extent of T cell expansion (**Supplementary Figure S2**).

To examine whether the expanded clonotypes might preferentially display features of T cell exhaustion, we examined the distribution of T cells with the top 20 most abundant clonotypes in each sample (**Figure 3D**). Smaller tumors contained a diverse array of CD8^+^ T cell subsets, including CD8Teff, CD8Tpex, and CD8Tex cell (KID001/002/018/016/006/015 in **Figure 3E**). In contrast, CD8Tex cells predominated in larger tumors (KID005/007/009/010 in **Figure 3E**). Although there were exceptions, such as KID012 and KID013, which showed CD8Tex-dominant clonotypes despite being smaller tumors, the immunological changes observed at the whole T cell populations (**Figure 1**), including the shift toward T cell exhaustion with tumor progression, were also evident within the clonally expanded T cell populations. Statistical comparisons across stages (**Supplementary Figure S3**) and between tumor size groups (**Supplementary Figure S4**) also showed a significant decrease in CD8Teff and CD8Tpex and an increase in CD8Tex in the advanced diseases. Collectively, these findings suggest that clonal expansion of TILs in advanced disease is accompanied by functional exhaustion, which may limit their anti-tumor efficacy.

### 3.4 Reactivity of the top 20 TCR clonotypes against autologous tumor-derived organoids

**Figure 4:**
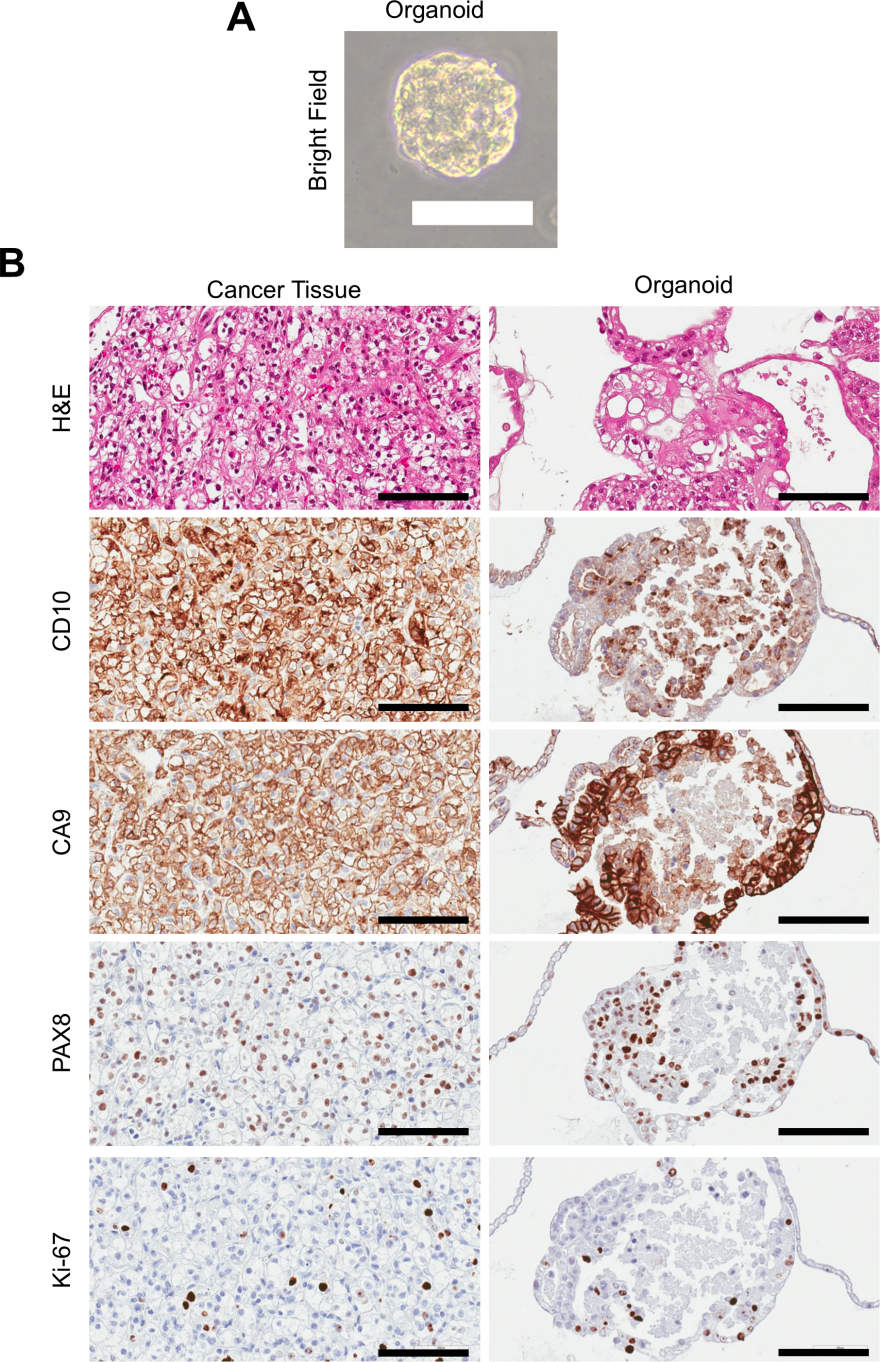
Establishment and phenotyping of ccRCC-derived organoids. **(A)** Representative phase-contrast microscopic image of a ccRCC-derived organoid (KID001). **(B)** Representative hematoxylin–eosin staining and immunohistochemical staining for ccRCC-related antigens (CD10, CA9, and PAX8) and proliferation marker (Ki-67) in KID001. Scale bars: 100 μm.

As an exploratory analysis, we further examined the reactivity of TCRs derived from TILs against autologous tumor-derived organoids from five ccRCC patients (KID001/002/005/007/010). Retroviral vectors encoding the Vα and Vβ cDNA sequences of the top 20 most abundant TCR clonotypes from each sample were transduced into Jurkat Δαβ CD8α cells to confirm proper TCR complex assembly, as assessed by surface CD3ϵ expression (**Supplementary Figure S1**). TCR expression was confirmed in nearly all top 20 clonotypes in each sample, except for one clonotype from KID001 and three clonotypes from KID005, which failed to yield functional TCRs (**Supplementary Table S3**). Meanwhile, organoids derived from autologous tumors were established and characterized by hematoxylin−eosin staining and immunohistochemical analyses for ccRCC−related antigens, including CD10, CA9, and PAX8, as well as the proliferation marker Ki-67. These organoids closely recapitulated the original tumors in both histopathology and antigen−expression profiles, suggesting their utility as surrogate models for native tumors, as shown in **Figure 4**; **Supplementary Figure S5**.

**Figure 5:**
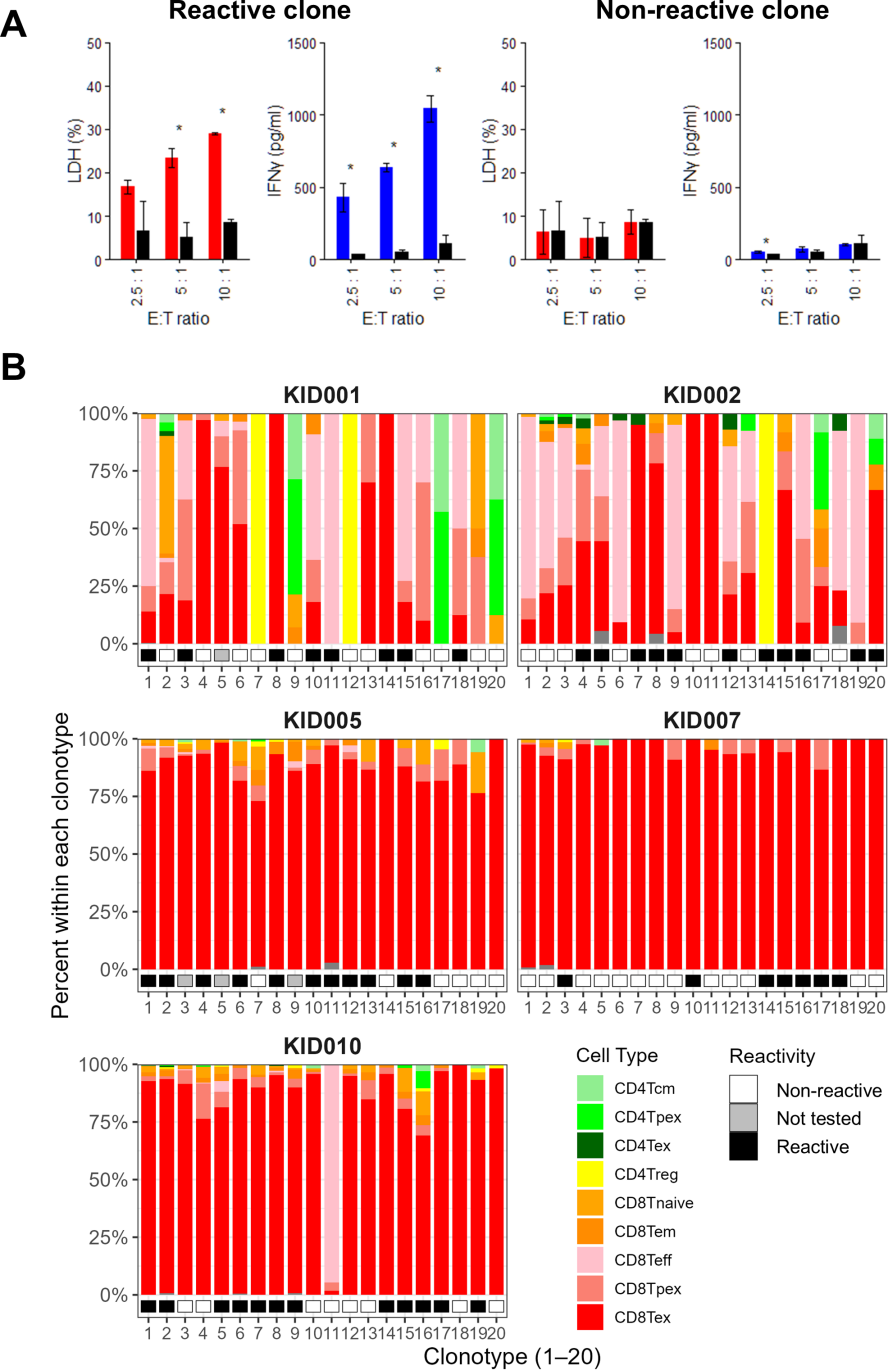
Gene expression profiles of organoid-reactive and -non-reactive T cells. **(A)** Representative results of *in vitro* LDH release (red bars) and IFN-γ secretion (blue bars) assays for TCR-T cells expressing an organoid-reactive clone (KID001#15) and a non-reactive clone (KID001#12). T cells expressing EGFP-containing expression vectors without TCR constructs served as controls (black bars). *P* values were determined by the Student’s t test (*< 0.05). **(B)** Cell type composition of T cell populations expressing each TCR among the top 20 most abundant clonotypes. Reactivity of each clonotype to autologous tumor-derived organoids is indicated by color bars. Cell types were assigned based on the annotated transcriptional clusters as shown in **Figure 1A**. Accordingly, some clonotypes may include cells annotated as both CD4^+^ and CD8^+^ T cells, reflecting limitations in clustering resolution.

PBMC from healthy donors were transfected with TCR-expressing vectors to generate TCR-T cells. These TCR-T cells were co-cultured with autologous tumor-derived organoids, and anti-tumor reactivity was assessed by LDH release assays and IFN-γ secretion measured by ELISA (**Figure 5A**; **Supplementary Figure S6**). With the criteria described in the Methods section, nearly half of the top 20 TCR clonotypes exhibited cytotoxicity and/or IFN-γ production, including 8 in KID001, 11 in KID002, 11 in KID005, 7 in KID007, and 12 in KID010 (**Figure 5B**; **Supplementary Figure S7**, **Supplementary Table S3**). The cell type composition of functionally accessed clonotypes indicated a Tex-skewed phenotype in advanced tumors (KID005, KID007 and KID010) while a more broadly distributed phenotype in early-stage tumors (KID001 and KID002), even among the tumor-reactive clonotypes.

Notably, in smaller tumors (KID001/002), TCR-T cells expressing CD4-derived TCRs demonstrated anti-tumor activity, suggesting the presence of cytotoxic CD4^+^ T cells (27, 28). Furthermore, as shown in **Supplementary Figure S7**, TCR clonotypes from smaller tumors displayed heterogeneous expression patterns of key genes, including effector molecule (IFNg), inhibitory receptors (PDCD1, CTLA4, HAVCR2, LAG3, TIGIT), and the exhaustion-associated factor (TOX). In contrast, clonotypes from larger tumors (KID005/007/010) exhibited uniformly higher expression patterns of these molecules. Importantly, many tumor-reactive TCRs were derived from clonotypes with exhausted phenotypes, yet retained anti-tumor reactivity when expressed in non-exhausted T cells. Collectively, these exploratory *in vitro* assays suggest that tumor-reactive TCRs can be identified even within phenotypically exhausted TILs in our cohort, providing proof-of-concept support for TCR-based approaches in ccRCC.

### 3.5 Gene signatures for prediction of tumor-specific T cells

Several recent studies have demonstrated transcriptomic signatures that are closely associated with tumor reactivity across various cancer types (21, 29–34). To evaluate whether such signatures could predict tumor-reactivity in ccRCC, we analyzed the gene expression profiles of tumor-reactive T cells. For this analysis, we used virus-specific bystander T cells as non-tumor-reactive controls (21, 35, 36), rather than organoid-non-reactive clones, because the latter might recognize tumor antigens that were present in the original tumors but lost during organoid establishment, leading to potential false-negative classification. In early-stage disease, naïve T cells were observed among bystander T cells but were not detected among tumor-reactive T cells; instead, tumor-reactive T cells were enriched for effector phenotypes. In advanced-stage disease, however, both tumor-reactive and bystander T cells showed an exhausted phenotype, raising the possibility that exhaustion is shaped not only by antigen stimulation but also by the tumor microenvironment (**Supplementary Figure S8**).

**Figure 6:**
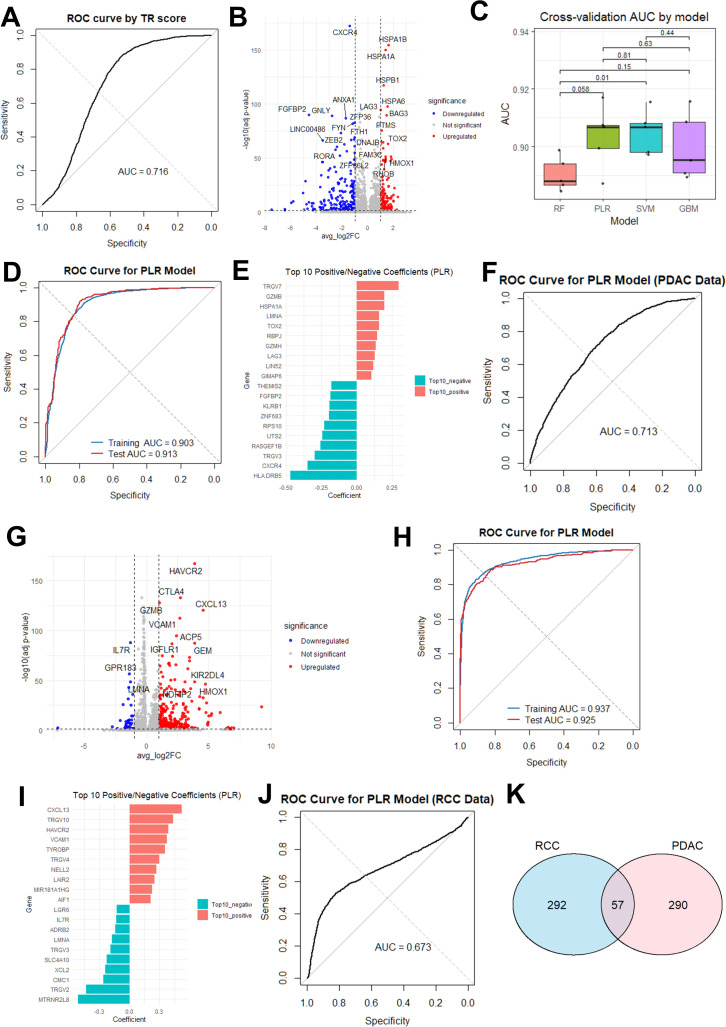
Gene signatures for prediction of tumor-specific T cells. **(A)** Receiver Operating Characteristic (ROC) analysis of our ccRCC dataset using a previously reported tumor reactivity (TR) score established in pancreatic ductal adenocarcinoma (PDAC) (21). AUC, area under the ROC curve. **(B)** Volcano plot of differentially expressed genes (DEGs) between CD8^+^ T cells expressing organoid-reactive TCRs and those expressing bystander TCRs harboring CDR3 amino acid motifs specific for CMV, EBV or influenza virus in our ccRCC dataset. Genes meeting the significance criteria (adjusted *p* < 0.05, nominal *p* < 0.05, and |log_2_FC| > 1) are plotted in red (upregulated) or blue (downregulated). **(C)** AUCs in five-fold cross-validation ROC analysis of our ccRCC dataset were compared among four different machine-learning models. RF, random forest; PLR, penalized logistic regression; SVM, support vector machines; GBM, gradient boosting machines. *P* values were determined using the Student’s t test. **(D)** ROC analysis for the training (blue) and test (red) sets in our ccRCC dataset using the PLR model trained on ccRCC-derived DEGs. **(E)** Top 10 genes with the largest positive and negative coefficients in the ccRCC-trained PLR model. **(F)** ROC analysis of the publicly available PDAC dataset (21) using the ccRCC-trained PLR model. **(G)** Volcano plot of DEGs between tumor-reactive and bystander CD8^+^ T cells in the publicly available PDAC dataset (21). Genes meeting the significance criteria (adjusted *p* < 0.05, nominal *p* < 0.05, and |log_2_FC| > 1) are plotted in red (upregulated) or blue (downregulated). **(H)** ROC analysis for the training (blue) and test (red) set in the PDAC dataset using the PLR model trained on PDAC-derived DEGs. **(I)** Top 10 genes with the largest positive and negative coefficients in the PDAC-trained PLR model. **(J)** ROC analysis for our ccRCC dataset using the PDAC-trained PLR model. **(K)** Overlap and distinct subsets of DEGs between ccRCC and PDAC were visualized using a Venn diagram.

First, we applied a previously reported scoring system for predicting anti-tumor responses, in which the tumor reactivity (TR) score was calculated based on a gene signature derived from the transcriptomic profiles of tumor-reactive CD8^+^ T cells in pancreatic ductal adenocarcinoma (PDAC) (21). When CD8^+^ T cells in our dataset were evaluated by the TR scoring system, the performance was substantially lower (AUC = 0.716), as shown in **Figure 6A**.

Next, we developed a prediction model for anti-tumor responses using four commonly-used machine learning approaches, including RF, PLR, SVM, and GBM, trained on our ccRCC dataset. By comparing the transcriptomic profiles of tumor-reactive T cells, as identified by organoid co-culture assays, with those of bystander T cells, we identified 349 DEGs (**Figure 6B**; **Supplementary Table S6**). Expression of these DEGs in tumor-reactive and bystander CD8^+^ T cells served as input for the machine-learning algorithms to generate a predictive model for tumor-specific T cells. As show in **Figure 6C**, comparisons among the four algorithms based on 5-fold cross-validation revealed no significant differences in performance, except that the RF model showed significantly lower performance than the SVM model (*p* = 0.01). Based on these results, we selected the PLR model for subsequent analyses because of its superior biological interpretability. The PLR model showed strong performance, with AUCs of 0.903 (training) and 0.913 (test) (**Figure 6D**). **Figure 6E** shows the top 10 variables with the largest positive and negative coefficients in the PLR model. For comparison, we applied the ccRCC-derived PLR model to the publicly available PDAC dataset used to develop the original TR scoring system (21). Unexpectedly, as shown in **Figure 6F**, the AUC value (0.713) was substantially lower than that reported for the original TR scoring system (AUC = 0.91) (21).

To further evaluate the validity of the PLR approach, we generated a PDAC-specific model using the PDAC dataset (21). By comparing the transcriptomic profiles of tumor-reactive T cells with those of bystander T cells, we identified 347 DEGs (**Figure 6G**; **Supplementary Table S7**). When the PLR algorithm was employed to construct a predictive model for tumor-specific T cells, this model predicted tumor-reactive T cells with high accuracy, with AUCs of 0.937 (training) and 0.925 (test) (**Figure 6H**), surpassing the performance of the original TR score (AUC = 0.91) (21). The top 10 variables with the largest positive and negative coefficients in the PDAC-specific model (**Figure 6I**) differed substantially from those identified in the ccRCC dataset (**Figure 6E**). In addition, when applied to predict tumor-reactive T cells in our ccRCC dataset, the performance was worse (AUC = 0.673) (**Figure 6J**). Collectively, these results suggest that accurate prediction of tumor-reactive T cells may require cancer type–specific models, underscoring the distinct transcriptomic signatures of tumor-reactive T cells in ccRCC and PDAC.

### 3.6 Differences in gene signatures between ccRCC and PDAC

When the lists of DEGs were compared between ccRCC and PDAC, only 57 genes were found to be shared (**Figure 6K**, **Supplementary Table S8**). To highlight the distinct transcriptomic signatures of tumor-reactive T cells in ccRCC and PDAC, the DEGs unique to ccRCC, unique to PDAC, and shared between the two cancers were categorized according to their biological functions, and representative genes from each category are summarized in **Table 1**. The shared DEGs reflects an immune activation/differentiation profile (e.g., CCR7, IL-7R, SOX4, TOX2) with modest proliferation programs (e.g., ASPM, CKAP2L, CLSPN, NCAPG, TOP2A). This indicates the presence of a conserved tumor-reactive T-cell compartment characterized by activation-associated cycling without dynamic clonal expansion.

**Table 1:** Representative differentially expressed genes (DEGs) categorized by gene functions.

Gene functions	Shared (ccRCC and PDAC)	ccRCC only	PDAC only
Cell cycle/proliferation	ASPM, CKAP2L, CLSPN, NCAPG, TOP2A	CDC14B, CDC25B	AURKB, BUB1B, CCNA2, CCNB2, CDC20, CDC45, CDC6, CDCA5, CDCA7L, CDK5, CDKN3, CENPM, CENPP, CENPU, CENPW, CKS1B, DLGAP5, ECT2, ESCO2, GTSE1, KIF11, KIF14, KIF15, KIF23, KIF2C, KIFC1, MCM2, MCM4, MCM5, MCM10, MKI67, NCAPG2, NCAPH, NUF2, NUSAP1, PCLAF, PKMYT1, POLD1, POLQ, RAD51, RAD51AP1, RRM2, SGO1, SKA1, SMC2, SPC25, TIMELESS, TK1, TPX2, TTK, TYMS, UBE2C, UHRF1, ZWINT.
Immune checkpoints and co-stimulatory	LAIR2	CD226, CD40LG, CD5, CD6, LAG3, LAIR1	PDCD1, CTLA4, HAVCR2, BTLA, ENTPD1, IL2RA, KLRG1, TNFRSF4, TNFRSF9, TNFRSF18, CD38.
Cytotoxic/effector	GZMB	FGFBP2, GNLY, GZMH, IFNG	NCR1
Cytokines/Chemokines and Receptors	CCR7, GPR183, S1PR1, XCL2, IL-7R, CSF1	CCL3, CCR6, CX3CR1, CXCR4, S1PR4, S1PR5, IFNGR1, IL10, IL12RB2, IL18R1	CXCL13, CXCR6, TNF
Adhesion/trafficking	AFAP1L2, ITGA5, PXN	SELPLG, SPON2	ITGA2, VCAM1
Stress/heat-shock	HMOX1	ATF3, GADD45G, HSPA1A, HSPA1B, HSPA6, HSPB1	
Transcription factors/regulators	BCL6, ID3, KLF3, SOX4, TOX2, RBPJ	BACH2, NR4A1, RORA, RORB, STAT4, TBX21, ZEB2, ZNF683	FOXP3, IKZF4, ID1, ETV1, ETV7, ZBTB32, ZFHX3, RUNX2

ccRCC, clear cell renal cell carcinoma; PDAC, pancreatic ductal adenocarcinoma.

Notably, the ccRCC-specific signature is less enriched for proliferation genes (limited to genes such as CDC14B and CDC25B), but instead exhibits expression of immune-regulatory and trafficking (e.g., IL-10, CCR6, CX3CR1, CXCR4), differentiation-associated (e.g., NR4A1, RORA, RORB, STAT4, TBX21, ZEB2), and stress-response (e.g., GADD45G, heat-shock proteins) genes. This pattern suggests that ccRCC TILs represent a more regulatory, functionally mature, and migratory state, potentially reflecting chronic antigen exposure and tissue adaptation through long-term immune surveillance. By contrast, PDAC-specific TILs exhibit a pronounced proliferative signature, with extensive enrichment of cell-cycle and DNA-replication genes (e.g., CCNA2/B2, CDC6/20, MCM2/4/5/10, TPX2, UBE2C). This is accompanied by broad expression of immune checkpoint and co-stimulatory genes (e.g., PDCD1, CTLA4, HAVCR2, BTLA, TNFRSF4/9/18), indicating dynamic clonal expansion with checkpoint engagement, possibly reflecting early phases of anti-tumor immune response.

Collectively, these findings suggest that tumor-reactive T cells share fundamental activation pathways across both tumor types, but diverge in the balance of proliferation, trafficking, and functional regulation reflecting the unique biological contexts of ccRCC and PDAC.

## 4 Discussion

This study aimed to elucidate the immunological landscape of ccRCC by performing single-cell transcriptomic analysis of TILs isolated from ccRCC specimens. Unlike many other solid tumors, ccRCC has been reported to exhibit an inverse correlation between the proportion of CD8^+^ T cells within TILs and clinical prognosis, a paradox that remains poorly understood (11–14). In this study, we suggested that, as tumor stage advances, CD8^+^ T cells within TILs undergo a dynamic compositional shift: the frequencies of CD8Teff and CD8Tpex cells declined significantly during tumor progression, whereas the proportion of CD8Tex cells increased markedly. This pattern suggests a progressive functional deterioration of the intratumoral CD8^+^ T cell compartment during tumor evolution. Although stage-dependent alterations in immune composition in ccRCC have been suggested in a previous single-cell work with a relatively small cohort (n=12) (37), our analysis of TILs from 15 patients reproduces and strengthens this observation, supporting the robustness of stage-associated immune remodeling in ccRCC.

Importantly, we further show that similar immunologic shifts correlate with primary tumor size —a core component of RCC staging— an association that, to our knowledge, has not been specifically examined in previous single-cell studies. According to the current TNM staging system, the T classification of RCC is determined by both tumor size and the anatomical extent of invasion beyond the kidney (1, 2). Tumors classified as T1 or T2 are staged based solely on size, while T3a tumors are defined by extrarenal fat, renal pelvis, and renal vein invasion, regardless of tumor diameter. Consequently, the pathological T3a (pT3a) category includes tumors of widely varying sizes, introducing potential prognostic heterogeneity within this group. To address this issue, several recent studies have stratified pT3aN0M0 (stage III) cases by tumor size, most commonly using a 7-cm cutoff (24–26). In this study, we also stratified pT3a RCC cases based on tumor size using a 7-cm cutoff and characterized the immunological composition of TILs through single-cell transcriptomic analysis, although dichotomization in this small cohort may introduce bias. We found that tumors >7-cm harbored a significantly higher proportion of CD8Tex cells compared with tumors ≤7-cm, whereas the frequencies of CD8 Teff and Tpex cells were reduced in tumors >7-cm. These findings suggest that larger tumors may promote a more immunosuppressive microenvironment, characterized by functional exhaustion of CD8^+^ T cells, which may contribute to impaired anti-tumor immunity and poorer clinical prognosis. Further studies involving larger cohorts and integrative analyses of both pathological and immunological parameters could refine risk stratification and guide treatment strategies for patients with pT3a RCC.

In addition, we suggested that the proportion of CD4Treg cells, as well as M2-polarized macrophages, increased in larger tumors, further indicating the emergence of an immunosuppressive tumor microenvironment. These alterations in the immune landscape likely contribute to the poor clinical outcomes observed in advanced ccRCC. Previous studies have similarly reported an increased presence of M2-polarized macrophages and/or CD4Treg cells together with CD8Tex cells within the tumor microenvironment, supporting the notion of a progressively immunosuppressive milieu in advanced disease (37–41). Collectively, our findings are consistent with, and extend, the emerging view that immune exhaustion and suppression are central drivers of immune evasion and disease progression in ccRCC.

Recent progress in organoid technology has enabled the development of patient-derived tumor organoids from a variety of cancers, including ccRCC (42, 43). These organoids recapitulate key features of the original tumor and offer a valuable platform for modeling the tumor microenvironment, *in vitro* drug screening, and evaluating immunotherapeutic responses (44–46). In particular, co-culture systems combining organoids with immune cells have emerged as powerful tools for assessing tumor-specific T cell reactivity *in vitro* (47–51). In this study, we investigated whether TCRs derived from clonally expanded TILs showed tumor reactivity using autologous tumor-derived organoids. To our knowledge, this is the first study in ccRCC to systematically validate the function of TCRs derived from dominant TIL clonotypes. Tumor-reactive TCRs were identified in all five cases analyzed, regardless of clinical stage. Notably, even in stage IV tumors, where most dominant TCRs originated from phenotypically exhausted CD8^+^ T cells, about half of these TCRs retained anti-tumor function when expressed in non-exhausted T cells. This finding suggests that functional reactivity can be restored through TCR-T cell engineering.

Recently, several studies have identified gene expression signatures that are closely associated with tumor-reactivity across various cancer types (29), including PDAC (21), melanoma (30), lung cancer (31, 32), gastrointestinal cancer (33), and metastatic cancer (34). In parallel with our functional validation experiments, we therefore performed transcriptional profiling of TILs and identified a gene expression signature characteristic of tumor-reactive CD8^+^ T cells. Using the model established by a PLR algorithm, we achieved strong predictive performance, with AUCs of 0.903 (training) and 0.913 (test), suggesting that gene expression-based classification can effectively enrich for tumor-reactive T cell clones in ccRCC. Nevertheless, when the model was applied to external datasets derived from PDAC (21), predictive performance declined markedly, indicating limited generalizability. Indeed, direct comparison of the top 10 positive and negative coefficients from the PLR models revealed substantial differences between ccRCC and PDAC, with minimal overlap among the genes most strongly associated with tumor reactivity (**Figures 6C, I**). These findings indicate that the transcriptional programs underlying tumor-reactive T cells are, at least in part, cancer type–specific and explains why the PDAC-defined TR score performed poorly in the ccRCC dataset.

Interestingly, detailed analyses of DEGs in tumor-reactive T cells revealed distinct immunogenomic landscapes between ccRCC and PDAC. Several genes associated with activation/differentiation and modest proliferation were shared, representing a conserved tumor-reactive subset present in both cancers. In contrast, ccRCC-specific TILs displayed a mature, functionally differentiated profile characterized by trafficking potential but limited proliferation, consistent with chronic antigen exposure and tissue adaptation. Conversely, PDAC-specific TILs exhibited highly proliferative and activated phenotypes, indicative of dynamic clonal expansion driven by strong antigenic stimulation. These findings emphasize that the balance between proliferation, differentiation, and trafficking diverges sharply between ccRCC and PDAC, reflecting tumor-intrinsic biology and the associated microenvironmental pressures. Such differences suggest that therapeutic approaches could be optimized to target the distinct biological properties of TILs in ccRCC and PDAC.

In summary, our integrative single-cell transcriptomic and functional profiling of TILs in ccRCC provides preliminary but new insights into the immune dynamics underlying disease progression. In addition, the protocol used to identify gene expression signatures in this study may serve as an exploratory approach to prioritize candidate tumor-specific TCRs for further functional evaluation. Nevertheless, several limitations should be acknowledged. First, analyses were performed in a cohort of 15 patients, with functional validation conducted in five patients, which may limit statistical power and preclude definitive conclusions regarding associations between immune features and clinical parameters. Second, functional validation of tumor-reactive TCRs was performed exclusively *in vitro* using autologous tumor-derived organoid co-culture systems, which may not fully recapitulate the complexity of *in vivo* immune–tumor interactions. Third, the precise antigens recognized by tumor-reactive TCRs, whether tumor-associated antigens or tumor-specific neoantigens, remain to be determined. Fourth, although the tumor-specific T-cell signature is promising in this exploratory dataset, it has not yet been validated in independent cohorts, as suitable public ccRCC single-cell datasets are currently unavailable. Accordingly, given the limited cohort size and the known heterogeneity of ccRCC, our findings should be interpreted as exploratory and hypothesis-generating, and will require validation in larger, independent ccRCC cohorts in future studies.

## Data Availability

The datasets presented in this study can be found in online repositories. The names of the repository/repositories and accession number(s) can be found below: https://www.ncbi.nlm.nih.gov/geo/, GSE304262; https://www.ncbi.nlm.nih.gov/geo/, GSE305993.
