# Supplementary material for: Tumor-reactive TCRs within exhausted TILs reveal cancer type-specific immune landscapes in renal cell carcinoma
Source: Front Immunol. 2026 Jan 29;17:1729388. doi: 10.3389/fimmu.2026.1729388 (PMC12894369; doi:10.3389/fimmu.2026.1729388)
Supplement: Supplementary file 1 [file DataSheet1.docx]

## Supplementary text 1

## R Script for analysis of single cell RNA sequence data and machine learning

# Load required libraries

library(Seurat)

library(dplyr)

library(tidyverse)

library(patchwork)

library(ggplot2)

library(ggrepel)

library(tibble)

library(VennDiagram)

library(pheatmap)

library(RColorBrewer)

library(viridis)

library(SingleR)

library(celldex)

library(SingleCellExperiment)

library(scRepertoire)

library(readr)

library(ggthemes)

library(MASS)

library(Matrix)

library(pROC)

library(caret)

library(gbm)

library(ggpubr)

library(ggtext)

library(purrr)

library(stringr)

library(tibble)

library(forcats)

library(ggnewscale)

library(tidyr)

library(scales)

library(UCell)

# Set working directory

setwd("path/to/workspace")

# ============================================

# Data Loading and Preprocessing

# ============================================

# Read 10X data and create Seurat objects

KID001.data <- Read10X(data.dir = "../KID001/sample_filtered_feature_bc_matrix")

KID001 <- CreateSeuratObject(counts = KID001.data, project = "KID001", min.cells = 3, min.features = 200)

KID002.data <- Read10X(data.dir = "../KID002/sample_filtered_feature_bc_matrix")

KID002 <- CreateSeuratObject(counts = KID002.data, project = "KID002", min.cells = 3, min.features = 200)

KID004.data <- Read10X(data.dir = "../KID004/sample_filtered_feature_bc_matrix")

KID004 <- CreateSeuratObject(counts = KID004.data, project = "KID004", min.cells = 3, min.features = 200)

KID005.data <- Read10X(data.dir = "../KID005/sample_filtered_feature_bc_matrix")

KID005 <- CreateSeuratObject(counts = KID005.data, project = "KID005", min.cells = 3, min.features = 200)

KID006.data <- Read10X(data.dir = "../KID006/sample_filtered_feature_bc_matrix")

KID006 <- CreateSeuratObject(counts = KID006.data, project = "KID006", min.cells = 3, min.features = 200)

KID007.data <- Read10X(data.dir = "../KID007/sample_filtered_feature_bc_matrix")

KID007 <- CreateSeuratObject(counts = KID007.data, project = "KID007", min.cells = 3, min.features = 200)

KID009.data <- Read10X(data.dir = "../KID009/sample_filtered_feature_bc_matrix")

KID009 <- CreateSeuratObject(counts = KID009.data, project = "KID009", min.cells = 3, min.features = 200)

KID010.data <- Read10X(data.dir = "../KID010/sample_filtered_feature_bc_matrix")

KID010 <- CreateSeuratObject(counts = KID010.data, project = "KID010", min.cells = 3, min.features = 200)

KID011.data <- Read10X(data.dir = "../KID011/sample_filtered_feature_bc_matrix")

KID011 <- CreateSeuratObject(counts = KID011.data, project = "KID011", min.cells = 3, min.features = 200)

KID012.data <- Read10X(data.dir = "../KID012/sample_filtered_feature_bc_matrix")

KID012 <- CreateSeuratObject(counts = KID012.data, project = "KID012", min.cells = 3, min.features = 200)

KID013.data <- Read10X(data.dir = "../KID013/sample_filtered_feature_bc_matrix")

KID013 <- CreateSeuratObject(counts = KID013.data, project = "KID013", min.cells = 3, min.features = 200)

KID014.data <- Read10X(data.dir = "../KID014/sample_filtered_feature_bc_matrix")

KID014 <- CreateSeuratObject(counts = KID014.data, project = "KID014", min.cells = 3, min.features = 200)

KID015.data <- Read10X(data.dir = "../KID015/sample_filtered_feature_bc_matrix")

KID015 <- CreateSeuratObject(counts = KID015.data, project = "KID015", min.cells = 3, min.features = 200)

KID016.data <- Read10X(data.dir = "../KID016/sample_filtered_feature_bc_matrix")

KID016 <- CreateSeuratObject(counts = KID016.data, project = "KID016", min.cells = 3, min.features = 200)

KID018.data <- Read10X(data.dir = "../KID018/sample_filtered_feature_bc_matrix")

KID018 <- CreateSeuratObject(counts = KID018.data, project = "KID018", min.cells = 3, min.features = 200)

# Calculate mitochondrial gene percentage

KID001[["percent.mt"]] <- PercentageFeatureSet(KID001, pattern = "^MT-")

KID002[["percent.mt"]] <- PercentageFeatureSet(KID002, pattern = "^MT-")

KID004[["percent.mt"]] <- PercentageFeatureSet(KID004, pattern = "^MT-")

KID005[["percent.mt"]] <- PercentageFeatureSet(KID005, pattern = "^MT-")

KID006[["percent.mt"]] <- PercentageFeatureSet(KID006, pattern = "^MT-")

KID007[["percent.mt"]] <- PercentageFeatureSet(KID007, pattern = "^MT-")

KID009[["percent.mt"]] <- PercentageFeatureSet(KID009, pattern = "^MT-")

KID010[["percent.mt"]] <- PercentageFeatureSet(KID010, pattern = "^MT-")

KID011[["percent.mt"]] <- PercentageFeatureSet(KID011, pattern = "^MT-")

KID012[["percent.mt"]] <- PercentageFeatureSet(KID012, pattern = "^MT-")

KID013[["percent.mt"]] <- PercentageFeatureSet(KID013, pattern = "^MT-")

KID014[["percent.mt"]] <- PercentageFeatureSet(KID014, pattern = "^MT-")

KID015[["percent.mt"]] <- PercentageFeatureSet(KID015, pattern = "^MT-")

KID016[["percent.mt"]] <- PercentageFeatureSet(KID016, pattern = "^MT-")

KID018[["percent.mt"]] <- PercentageFeatureSet(KID018, pattern = "^MT-")

# Visualize QC metrics

VlnPlot(KID001, features = c("nFeature_RNA", "nCount_RNA", "percent.mt"), ncol = 3)

VlnPlot(KID002, features = c("nFeature_RNA", "nCount_RNA", "percent.mt"), ncol = 3)

VlnPlot(KID004, features = c("nFeature_RNA", "nCount_RNA", "percent.mt"), ncol = 3)

VlnPlot(KID005, features = c("nFeature_RNA", "nCount_RNA", "percent.mt"), ncol = 3)

VlnPlot(KID006, features = c("nFeature_RNA", "nCount_RNA", "percent.mt"), ncol = 3)

VlnPlot(KID007, features = c("nFeature_RNA", "nCount_RNA", "percent.mt"), ncol = 3)

VlnPlot(KID009, features = c("nFeature_RNA", "nCount_RNA", "percent.mt"), ncol = 3)

VlnPlot(KID010, features = c("nFeature_RNA", "nCount_RNA", "percent.mt"), ncol = 3)

VlnPlot(KID011, features = c("nFeature_RNA", "nCount_RNA", "percent.mt"), ncol = 3)

VlnPlot(KID012, features = c("nFeature_RNA", "nCount_RNA", "percent.mt"), ncol = 3)

VlnPlot(KID013, features = c("nFeature_RNA", "nCount_RNA", "percent.mt"), ncol = 3)

VlnPlot(KID014, features = c("nFeature_RNA", "nCount_RNA", "percent.mt"), ncol = 3)

VlnPlot(KID015, features = c("nFeature_RNA", "nCount_RNA", "percent.mt"), ncol = 3)

VlnPlot(KID016, features = c("nFeature_RNA", "nCount_RNA", "percent.mt"), ncol = 3)

VlnPlot(KID018, features = c("nFeature_RNA", "nCount_RNA", "percent.mt"), ncol = 3)

# QC filtering based on visualization results

KID001 <- subset(KID001, subset = nFeature_RNA > 200 & nFeature_RNA < 2500 & percent.mt < 5)

KID002 <- subset(KID002, subset = nFeature_RNA > 200 & nFeature_RNA < 1800 & percent.mt < 5)

KID004 <- subset(KID004, subset = nFeature_RNA > 200 & nFeature_RNA < 2500 & percent.mt < 5)

KID005 <- subset(KID005, subset = nFeature_RNA > 200 & nFeature_RNA < 2600 & percent.mt < 5)

KID006 <- subset(KID006, subset = nFeature_RNA > 200 & nFeature_RNA < 2500 & percent.mt < 5)

KID007 <- subset(KID007, subset = nFeature_RNA > 200 & nFeature_RNA < 2500 & percent.mt < 5)

KID009 <- subset(KID009, subset = nFeature_RNA > 200 & nFeature_RNA < 3500 & percent.mt < 5)

KID010 <- subset(KID010, subset = nFeature_RNA > 200 & nFeature_RNA < 3500 & percent.mt < 5)

KID011 <- subset(KID011, subset = nFeature_RNA > 200 & nFeature_RNA < 5000 & percent.mt < 5)

KID012 <- subset(KID012, subset = nFeature_RNA > 200 & nFeature_RNA < 5000 & percent.mt < 5)

KID013 <- subset(KID013, subset = nFeature_RNA > 200 & nFeature_RNA < 3500 & percent.mt < 5)

KID014 <- subset(KID014, subset = nFeature_RNA > 200 & nFeature_RNA < 2100 & percent.mt < 5)

KID015 <- subset(KID015, subset = nFeature_RNA > 200 & nFeature_RNA < 2100 & percent.mt < 5)

KID016 <- subset(KID016, subset = nFeature_RNA > 200 & nFeature_RNA < 2100 & percent.mt < 7)

KID018 <- subset(KID018, subset = nFeature_RNA > 200 & nFeature_RNA < 2100 & percent.mt < 7)

# Data normalization

KID001 <- NormalizeData(KID001)

KID002 <- NormalizeData(KID002)

KID004 <- NormalizeData(KID004)

KID005 <- NormalizeData(KID005)

KID006 <- NormalizeData(KID006)

KID007 <- NormalizeData(KID007)

KID009 <- NormalizeData(KID009)

KID010 <- NormalizeData(KID010)

KID011 <- NormalizeData(KID011)

KID012 <- NormalizeData(KID012)

KID013 <- NormalizeData(KID013)

KID014 <- NormalizeData(KID014)

KID015 <- NormalizeData(KID015)

KID016 <- NormalizeData(KID016)

KID018 <- NormalizeData(KID018)

# Merge all samples

KIDall <- merge(KID001, y = c(KID002, KID004, KID005, KID006, KID007, KID009, KID010, KID011, KID012, KID013, KID014, KID015, KID016, KID018),

add.cell.ids = c("KID001", "KID002", "KID004", "KID005", "KID006", "KID007", "KID009", "KID010", "KID011", "KID012", "KID013", "KID014", "KID015", "KID016", "KID018"), project = "KIDall", merge.data = TRUE)

# ============================================

# Standard Analysis Workflow

# ============================================

KIDall <- NormalizeData(KIDall)

KIDall <- FindVariableFeatures(KIDall, selection.method = "vst", nfeatures = 2000)

all.genes <- rownames(KIDall)

KIDall <- ScaleData(KIDall, features = all.genes)

KIDall <- RunPCA(KIDall, features = VariableFeatures(object = KIDall))

KIDall <- FindNeighbors(KIDall, dims = 1:10, reduction = "pca")

KIDall <- FindClusters(KIDall, resolution = 2, cluster.name = "unintegrated_clusters")

KIDall <- RunUMAP(KIDall, dims = 1:10, reduction = "pca", reduction.name = "umap.unintegrated")

DimPlot(KIDall, reduction = "umap.unintegrated", group.by = c("orig.ident", " unintegrated_clusters"))

#perform integration

KIDall <- IntegrateLayers(object = KIDall, method = CCAIntegration, orig.reduction = "pca", new.reduction = "integrated.cca",verbose = FALSE)

# re-join layers after integration

KIDall[["RNA"]] <- JoinLayers(KIDall[["RNA"]])

KIDall <- FindNeighbors(KIDall, reduction = "integrated.cca", dims = 1:10)

KIDall <- FindClusters(KIDall, resolution = 2)

KIDall <- RunUMAP(KIDall, dims = 1:10, reduction = "integrated.cca")

# Visualization

DimPlot(KIDall, reduction = "umap", group.by = c("orig.ident", "seurat_clusters"))

# find markers for every cluster compared to all remaining cells, report only the positive ones

KIDall.markers <- FindAllMarkers(KIDall, only.pos = TRUE)

top5 <- KIDall.markers %>%

filter(avg_log2FC > 1) %>%

filter(p_val_adj < 0.05) %>%

group_by(cluster) %>%

slice_max(order_by = avg_log2FC, n = 5, with_ties = FALSE) %>%

ungroup()

# display expression of representative genes in each cluster

DotPlot(KIDall, features = c('CD3E',

'CD4',

'CD8A',

'CCR7',

'SELL',

'TCF7',

'EOMES',

'DKK3',

'GPR183',

'CXCR4',

'FGFBP2',

'FCGR3A',

'GZMA',

'GZMB',

'NKG7',

'GNLY',

'RORA',

'IL17A',

'CXCL13',

'TNFRSF4',

'PDCD1',

'HAVCR2',

'TOX',

'LAG3',

'CTLA4',

'TIGIT',

'FOXP3'), scale.max = 100, scale.min = 0)+ RotatedAxis()

# manual annotation to each cluster

new.cluster.ids <- c('CD8Tex',

'CD8Tex',

'CD8Tex',

'CD4Tcm',

'CD8Tex',

'CD8Tpex',

'CD4Tpex',

'CD4Tcm',

'CD8Tnaive',

'CD4Tcm',

'CD8Tex',

'CD8Teff',

'CD4Treg',

'CD8Tex',

'CD8Tnaive',

'CD8Teff',

'CD4Tpex',

'CD8Tpex',

'CD8Teff',

'CD8Tex',

'CD4Treg',

'CD8Tex',

'CD8Tex',

'CD8Tex',

'CD8Tem',

'non-T',

'non-T',

'CD8Tex',

'non-T',

'CD4Tex',

'CD8Tem',

'non-T',

'CD4Treg',

'non-T',

'non-T')

names(new.cluster.ids) <- levels(KIDall)

KIDall_rename <- RenameIdents(KIDall, new.cluster.ids)

levels(KIDall_rename) <- c('CD4Tcm',

'CD4Tpex',

'CD4Tex',

'CD4Treg',

'CD8Tnaive',

'CD8Tem',

'CD8Teff',

'CD8Tpex',

'CD8Tex',

'non-T')

KIDall_rename$celltype <- Idents(KIDall_rename)

DimPlot(KIDall_rename, reduction = "umap", label = TRUE, pt.size = 0.5) + NoLegend()

# <<<Figure 1A>>>

p <- DimPlot(KIDall_rename, reduction = "umap", pt.size = 0.5) + NoLegend()

LabelClusters(plot = p, id = "ident", repel = TRUE, box = FALSE, size = 6)

# <<<Figure 1B>>>

DotPlot(KIDall_rename, features = c('CD3E',

'CD4',

'CD8A',

'CCR7',

'SELL',

'TCF7',

'EOMES',

'DKK3',

'GPR183',

'CXCR4',

'FGFBP2',

'FCGR3A',

'GZMA',

'GZMB',

'NKG7',

'GNLY',

'RORA',

'IL17A',

'CXCL13',

'TNFRSF4',

'PDCD1',

'HAVCR2',

'TOX',

'LAG3',

'CTLA4',

'TIGIT',

'FOXP3'), scale.max = 100, scale.min = 0)+ RotatedAxis() +

theme(

axis.text.y = element_text(size = 14, face = "bold"),

axis.text.x = element_text(angle = 90, size = 14, vjust = 0.5)

)

# ============================================

# Cluster Composition Analysis

# ============================================

## ---- Cluster composition per sample (count + percent) ----

# Count the number of cells for each (sample × cell type / cluster)

mat_count <- table(KIDall_rename$orig.ident, Idents(KIDall_rename))

# Convert the count matrix to a data frame (rows = samples, columns = cell types / clusters)

df_count <- as.data.frame.matrix(mat_count)

write.csv(df_count, file = "stat_count.csv", quote = FALSE)

# Calculate percentage of each cluster within each sample (row-wise normalization)

mat_pct <- prop.table(mat_count, margin = 1) * 100

df_pct <- as.data.frame.matrix(mat_pct)

write.csv(df_pct, file = "stat_pct.csv", quote = FALSE)

# ============================================

# Barcode-Level Metadata Integration

# ============================================

# Create a lookup table linking cell barcodes to sample IDs (orig.ident)

barcode_df <- tibble::tibble(

barcode = Cells(KIDall_rename),

orig.ident = KIDall_rename$orig.ident

)

# Load external barcode-level reactivity annotation

# IMPORTANT: the CSV file must contain a column named "barcode"

reactivity_raw <- read.csv("path/to/KID125710_barcode_reactivity.csv", header = TRUE)

reactivity_raw <- reactivity_raw[,-1]

# Merge reactivity information while keeping all Seurat cells (left join)

reactivity_merged <- dplyr::left_join(barcode_df, reactivity_raw, by = "barcode")

# Replace empty strings ("") with NA for safer downstream handling,

# then optionally fill missing values with "nonT"

reactivity_merged <- reactivity_merged %>%

dplyr::mutate(dplyr::across(dplyr::everything(), ~ dplyr::na_if(., "")))

# Explicitly fill NA values (except barcode and orig.ident) with "nonT"

cols_to_fill <- setdiff(colnames(reactivity_merged), c("barcode", "orig.ident"))

reactivity_merged <- reactivity_merged %>%

dplyr::mutate(dplyr::across(dplyr::all_of(cols_to_fill), ~ ifelse(is.na(.), "nonT", .)))

# Convert to a data frame with barcodes as row names for AddMetaData

reactivity_meta <- reactivity_merged %>%

tibble::column_to_rownames("barcode")

# Add the reactivity metadata to the Seurat object

KIDall_rename2 <- AddMetaData(

object = KIDall_rename,

metadata = reactivity_meta

)

# ============================================

# Incorporate Repertoire Information

# ============================================

# Load TCR clonotype data

KID001.clone <- read.csv("../KID001/filtered_contig_annotations.csv", header=T)

KID002.clone <- read.csv("../KID002/filtered_contig_annotations.csv", header=T)

KID004.clone <- read.csv("../KID004/filtered_contig_annotations.csv", header=T)

KID005.clone <- read.csv("../KID005/filtered_contig_annotations.csv", header=T)

KID006.clone <- read.csv("../KID006/filtered_contig_annotations.csv", header=T)

KID007.clone <- read.csv("../KID007/filtered_contig_annotations.csv", header=T)

KID009.clone <- read.csv("../KID009/filtered_contig_annotations.csv", header=T)

KID010.clone <- read.csv("../KID010/filtered_contig_annotations.csv", header=T)

KID011.clone <- read.csv("../KID011/filtered_contig_annotations.csv", header=T)

KID012.clone <- read.csv("../KID012/filtered_contig_annotations.csv", header=T)

KID013.clone <- read.csv("../KID013/filtered_contig_annotations.csv", header=T)

KID014.clone <- read.csv("../KID014/filtered_contig_annotations.csv", header=T)

KID015.clone <- read.csv("../KID015/filtered_contig_annotations.csv", header=T)

KID016.clone <- read.csv("../KID016/filtered_contig_annotations.csv", header=T)

KID018.clone <- read.csv("../KID018/filtered_contig_annotations.csv", header=T)

# Combine TCR data

contig_list <- list(KID001.clone, KID002.clone, KID004.clone, KID005.clone, KID006.clone, KID007.clone, KID009.clone, KID010.clone, KID011.clone, KID012.clone, KID013.clone, KID014.clone, KID015.clone, KID016.clone, KID018.clone)

combined <- combineTCR(contig_list,

samples = c("KID001", "KID002", "KID004", "KID005", "KID006", "KID007", "KID009", "KID010", "KID011", "KID012", "KID013", "KID014", "KID015", "KID016", "KID018"))

clonalAbundance(combined,

cloneCall = "gene",

scale = FALSE)

# Combine expression and TCR data

KIDall.vdj_R <- combineExpression(combined, KIDall_rename2)

## add clonotype_id

obj_names <- c("KID001.clone","KID002.clone","KID004.clone","KID005.clone","KID006.clone",

"KID007.clone","KID009.clone","KID010.clone","KID011.clone","KID012.clone",

"KID013.clone","KID014.clone","KID015.clone","KID016.clone","KID018.clone")

df_all <- bind_rows(lapply(obj_names, function(obj_nm) {kid_prefix <- sub("\\.clone$", "_", obj_nm)

x <- get(obj_nm, envir = .GlobalEnv)

as_tibble(x) %>%

transmute(

barcode = paste0(kid_prefix, barcode),

clonotype_id = paste0(kid_prefix, raw_clonotype_id)

)

}))

df_all <- df_all %>% distinct(barcode, .keep_all = TRUE)

meta <- KIDall.vdj_R@meta.data %>%

rownames_to_column("barcode") %>%

left_join(df_all, by = "barcode") %>%

column_to_rownames("barcode")

KIDall.vdj_R@meta.data <- meta

# ============================================

# Identification of Bystander T Cells Using TCR Sequence Information

# ============================================

df <- KIDall.vdj_R@meta.data %>%

dplyr::select(CTaa) %>%

mutate(

cdr3b_aa = CTaa %>%

sub(".*_", "", .) %>%

str_sub(2, -2)

)

dfu <- distinct(df)

# Run TCRmatch externally and load the results from "output_file.csv"

# Python code

# ./tcrmatch -i input_file.tsv -t 8 -d data/IEDB_data.tsv > output_file./tcrmatch -i input_file.tsv -t 8 -d data/IEDB_data.tsv > output_file

# end of Python code

output <- read.csv("output_file.csv")

output <- dplyr::select(output, c(trimmed_input_sequence, organism))

output$organism <- sub(",.*", "", output$organism)

colnames(output) <- c("cdr3b_aa", "specificity")

output <- distinct(output, cdr3b_aa, .keep_all = TRUE)

df$barcode <- rownames(df)

dfdb <- merge(df, output, by="cdr3b_aa", all.x=TRUE)

rownames(dfdb)<-dfdb$barcode

dfdb <- dplyr::select(dfdb, specificity)

KIDall.vdj_R_bys <- AddMetaData(object=KIDall.vdj_R, metadata=dfdb)

# Update the custom_group annotation based on reactivity and specificity

KIDall.vdj_R_bys$custom_group <- "other"

KIDall.vdj_R_bys$custom_group[KIDall.vdj_R_bys$reactivity == "8Y"] <- "8Y"

KIDall.vdj_R_bys$custom_group[grepl("cytomegalo|Epstein|Influenza", KIDall.vdj_R_bys$specificity, ignore.case = TRUE)] <- "Bys"

# ============================================

# Calculation of Clonal Diversity in CD8+ T Cells

# ============================================

# Place filtered_contig_annotations.csv files (renamed by sample ID)

# together with metadata files under the DATA/ directory

# Restrict analysis to CD8+ T cells

KIDall.vdj_R_bys8<-subset(KIDall.vdj_R_bys, CD8A>0 & CD4==0)

bcd <- tibble::tibble(

barcode = Cells(KIDall.vdj_R_bys8),

orig.ident = KIDall.vdj_R_bys8$orig.ident

)

ids <- c("KID001","KID002","KID004","KID005","KID006","KID007","KID009","KID010","KID011","KID012","KID013","KID014","KID015","KID016","KID018")

dir.create("DATA/CD8", recursive = TRUE, showWarnings = FALSE)

for (id in ids) {

bcd_sub <- subset(bcd, orig.ident %in% id)

bcd_sub$barcode <- sub(".*_", "", bcd_sub$barcode)

df <- read.csv(paste0("DATA/", id, ".csv")) %>%

filter(barcode %in% bcd_sub$barcode)

write.csv(df, file = paste0("DATA/CD8/", id, ".csv"), row.names = FALSE)

}

# Define input and output directories

input_dir <- "DATA/CD8/"

output_dir <- "DATA/CD8/immunarch_inputs/"

# Create output directory if it does not exist

if (!dir.exists(output_dir)) dir.create(output_dir)

# Process each sample independently

for (id in ids) {

# read input file

file_path <- paste0(input_dir, id, ".csv")

df <- read.csv(file_path)

# Check that all required columns are present

if (!all(c("cdr3_nt", "cdr3", "v_gene", "j_gene", "d_gene", "raw_clonotype_id") %in% colnames(df))) {

message(paste("Skip: ", id, "（column not found）"))

next

}

# Filter out rows with missing essential information

df <- df %>%

filter(!is.na(cdr3_nt) & !is.na(cdr3) & !is.na(v_gene) & !is.na(j_gene))

# Define one clone by grouping cells with the same raw_clonotype_id

clones <- df %>%

group_by(raw_clonotype_id) %>%

summarise(

Clones = dplyr::n(),

CDR3.nt = dplyr::first(cdr3_nt),

CDR3.aa = dplyr::first(cdr3),

V.name = dplyr::first(v_gene),

D.name = dplyr::first(d_gene),

J.name = dplyr::first(j_gene),

.groups = "drop"

) %>%

mutate(Proportion = Clones / sum(Clones)) %>%

dplyr::select(Clones, Proportion, CDR3.nt, CDR3.aa, V.name, D.name, J.name)

# Write immunarch-compatible clonotype table to file

out_file <- paste0(output_dir, id, ".csv")

write.csv(clones, out_file, row.names = FALSE)

message(paste("done: ", id))

}

library(immunarch)

# Read filtered_contig_annotations.csv and metadata

immdata8 <- repLoad("DATA/CD8/immunarch_inputs/")

exp_vol <- repExplore(immdata8$data, .method = "volume")

# Calculate diversity metrics

imm_pr <- repClonality(immdata8$data, .method = "clonal.prop")

imm_pr

imm_hom <- repClonality(immdata8$data,

.method = "homeo",

.clone.types = c(Small = .0001, Medium = .001, Large = .01, Hyperexpanded = 1)

)

imm_hom

vis(imm_hom, .by = c("Stage"), .meta = immdata8$meta)

imm_rare <- repClonality(immdata8$data, .method = "rare")

imm_rare

#Shannon Index

DivTV <- repDiversity(.data = immdata8$data, .method = "div", .q = 5, .do.norm = NA, .laplace = 0)

write.csv(DivTV, file="ShannonIndex8.csv")

# open stat.csv externally and add metadata for each sample

# (such as tumor size, disease stage, Shannon Index of CD8T cells, etc.)

# save stat.csv

# ============================================

# Statistical Analysis and Visualization

# ============================================

# Load data with additional metadata

df <- read.csv("stat.csv", row.names = 1, check.names = FALSE)

# Explicitly specify the columns to use

cell_cols <- c('CD4Tcm',

'CD4Tpex',

'CD4Tex',

'CD4Treg',

'CD8Tnaive',

'CD8Tem',

'CD8Teff',

'CD8Tpex',

'CD8Tex',

'non-T')

cell_colors <- c('CD4Tcm' ="lightgreen",

'CD4Tpex' = "green",

'CD4Tex' = "darkgreen",

'CD4Treg' = "yellow",

'CD8Tnaive' = "orange",

'CD8Tem' = "darkorange",

'CD8Teff' = "pink",

'CD8Tpex' = "salmon",

'CD8Tex' = "red",

'non-T' = "darkgray")

# Convert to long format

df_long <- df %>%

dplyr::select(all_of(cell_cols)) %>%

mutate(Sample = rownames(.)) %>%

pivot_longer(cols = all_of(cell_cols),

names_to = "CellType",

values_to = "Percent")

# Set the order of CellType levels

df_long$CellType <- factor(df_long$CellType, levels = cell_cols)

# Order by tumor diameter

sample_order <- c('KID001',

'KID002',

'KID018',

'KID012',

'KID016',

'KID013',

'KID006',

'KID015',

'KID011',

'KID004',

'KID014',

'KID005',

'KID007',

'KID009',

'KID010')

# Convert Sample to an ordered factor

df_long$Sample <- factor(df_long$Sample, levels = sample_order)

# <<<Figure 1C>>>

ggplot(df_long, aes(x = Sample, y = Percent, fill = CellType)) +

geom_bar(stat = "identity") +

scale_fill_manual(values = cell_colors) +

labs(x = "Sample", y = "Proportion (%)", fill = "Cell Type") +

theme_minimal() +

theme(axis.text.x = element_text(size = 12, angle = 90, hjust = 0, vjust = 0.5))

# <<<Figure 1D>>>

a<-ggplot(df, aes(x = stage, y = CD8Tex_in_CD8T, fill = stage)) +

geom_boxplot() +

geom_jitter(width = 0.2, alpha = 0.5) +

#geom_text(aes(label = Sample), vjust = -0.5, size = 3) + # Show labels slightly above the points

labs(title = "CD8Tex/CD8T",

x = "Stage", y = "Percentage") +

stat_compare_means(comparisons = list(c("I", "III"), c("I","IV"), c("III","IV")),

method = "t.test",

method.args = list(var.equal = TRUE, paired = FALSE),

label = "p.format", size = 4) +

theme_minimal()+

theme(axis.text.x = element_text(size = 16, face = "bold", angle = 0, hjust = 0.5),

axis.title.y = element_text(size = 16, face = "bold"),

legend.position = "none")

b<-ggplot(df, aes(x = stage, y = CD8Teff_in_CD8T, fill = stage)) +

geom_boxplot() +

geom_jitter(width = 0.2, alpha = 0.5) +

labs(title = "CD8Teff/CD8T",

x = "Stage", y = "Percentage") +

stat_compare_means(comparisons = list(c("I", "III"), c("I","IV"), c("III","IV")),

method = "t.test",

method.args = list(var.equal = TRUE, paired = FALSE),

label = "p.format", size = 4) +

theme_minimal()+

theme(axis.text.x = element_text(size = 16, face = "bold", angle = 0, hjust = 0.5),

axis.title.y = element_text(size = 16, face = "bold"),

legend.position = "none")

c<-ggplot(df, aes(x = stage, y = CD8Tpex_in_CD8T, fill = stage)) +

geom_boxplot() +

geom_jitter(width = 0.2, alpha = 0.5) +

#geom_text(aes(label = Sample), vjust = -0.5, size = 3) + # Show labels slightly above the points

labs(title = "CD8Tpex/CD8T",

x = "Stage", y = "Percentage") +

stat_compare_means(comparisons = list(c("I", "III"), c("I","IV"), c("III","IV")),

method = "t.test",

method.args = list(var.equal = TRUE, paired = FALSE),

label = "p.format", size = 4) +

theme_minimal()+

theme(axis.text.x = element_text(size = 16, face = "bold", angle = 0, hjust = 0.5),

axis.title.y = element_text(size = 16, face = "bold"),

legend.position = "none")

d<-ggplot(df, aes(x = stage, y = CD4Treg_in_CD4T, fill = stage)) +

geom_boxplot() +

geom_jitter(width = 0.2, alpha = 0.5) +

#geom_text(aes(label = Sample), vjust = -0.5, size = 3) + # Show labels slightly above the points

labs(title = "CD4Treg/CD4T",

x = "Stage", y = "Percentage") +

stat_compare_means(comparisons = list(c("I", "III"), c("I","IV"), c("III","IV")),

method = "t.test",

method.args = list(var.equal = TRUE, paired = FALSE),

label = "p.format", size = 4) +

theme_minimal()+

theme(axis.text.x = element_text(size = 16, face = "bold", angle = 0, hjust = 0.5),

axis.title.y = element_text(size = 16, face = "bold"),

legend.position = "none")

plot_grid(b, c, a, d, ncol = 4)

# <<<Figure 1F>>>

df$size <- factor(df$size, levels = c("≤7cm", ">7cm"), labels = c("≤7cm", ">7cm"))

a<-ggplot(df, aes(x = size, y = CD8Tex_in_CD8T, fill = size)) +

geom_boxplot(alpha = 0.6) +

geom_jitter(width = 0.2, alpha = 0.5) +

labs(title = "CD8Tex/CD8T",

x = "Tumor Diameter", y = "Percentage") +

stat_compare_means(method = "t.test",

method.args = list(var.equal = TRUE, paired = FALSE),

label = "p.format", label.y = 95, size = 5) +

theme_minimal()+

theme(legend.position = "none",

axis.text.x = element_text(size = 16),

axis.title.x = element_text(size = 16),

axis.title.y = element_text(size = 16))

b<-ggplot(df, aes(x = size, y = CD8Teff_in_CD8T, fill = size)) +

geom_boxplot(alpha = 0.6) +

geom_jitter(width = 0.2, alpha = 0.5) +

labs(title = "CD8Teff/CD8T",

x = "Tumor Diameter", y = "Percentage") +

stat_compare_means(method = "t.test",

method.args = list(var.equal = TRUE, paired = FALSE),

label = "p.format", label.y = 35, size = 5) +

theme_minimal()+

theme(legend.position = "none",

axis.text.x = element_text(size = 16),

axis.title.x = element_text(size = 16),

axis.title.y = element_text(size = 16))

c<-ggplot(df, aes(x = size, y = CD8Tpex_in_CD8T, fill = size)) +

geom_boxplot(alpha = 0.6) +

geom_jitter(width = 0.2, alpha = 0.5) +

labs(title = "CD8Tpex/CD8T",

x = "Tumor Diameter", y = "Percentage") +

stat_compare_means(method = "t.test",

method.args = list(var.equal = TRUE, paired = FALSE),

label = "p.format", label.y = 20, size = 5) +

theme_minimal()+

theme(legend.position = "none",

axis.text.x = element_text(size = 16),

axis.title.x = element_text(size = 16),

axis.title.y = element_text(size = 16))

d<-ggplot(df, aes(x = size, y = CD4Treg_in_CD4T, fill = size)) +

geom_boxplot(alpha = 0.6) +

geom_jitter(width = 0.2, alpha = 0.5) +

labs(title = "CD4Treg/CD4T",

x = "Tumor Diameter", y = "Percentage") +

stat_compare_means(method = "t.test",

method.args = list(var.equal = TRUE, paired = FALSE),

label = "p.format", label.y = 75, size = 5) +

theme_minimal()+

theme(legend.position = "none",

axis.text.x = element_text(size = 16),

axis.title.x = element_text(size = 16),

axis.title.y = element_text(size = 16))

plot_grid(b, c, a, d, ncol = 4)

# <<<Figure 1E>>>

a <- ggplot(df, aes(x = diameter, y = CD8Teff_in_CD8T)) +

geom_point(size = 3, alpha = 0.7) +

geom_smooth(method = "lm", se = TRUE, color = "blue") +

stat_cor(method = "pearson", label.x = 10, label.y = 40, size = 5,

aes(label = paste(..r.label.., ..p.label.., sep = "~`,`~"))) +

labs(title = "CD8Teff / CD8T",

x = "Tumor Diameter",

y = "Percentage") +

theme_minimal() +

theme(axis.text.x = element_text(hjust = 1,size = 16),

axis.title.x = element_text(size = 16),

axis.title.y = element_text(size = 16))

b <- ggplot(df, aes(x = diameter, y = CD8Tpex_in_CD8T)) +

geom_point(size = 3, alpha = 0.7) +

geom_smooth(method = "lm", se = TRUE, color = "blue") +

stat_cor(method = "pearson", label.x = 10, label.y = 20, size = 5,

aes(label = paste(..r.label.., ..p.label.., sep = "~`,`~"))) +

labs(title = "CD8Tpex / CD8T",

x = "Tumor Diemater",

y = "Percentage") +

theme_minimal() +

theme(axis.text.x = element_text(hjust = 1,size = 16),

axis.title.x = element_text(size = 16),

axis.title.y = element_text(size = 16))

c <- ggplot(df, aes(x = diameter, y = CD8Tex_in_CD8T)) +

geom_point(size = 3, alpha = 0.7) +

geom_smooth(method = "lm", se = TRUE, color = "blue") +

stat_cor(method = "pearson", label.x = 10, label.y = 120, size = 5,

aes(label = paste(..r.label.., ..p.label.., sep = "~`,`~"))) +

labs(title = "CD8Tex / CD8T",

x = "Tumor Diameter",

y = "Percentage") +

theme_minimal() +

theme(axis.text.x = element_text(hjust = 1,size = 16),

axis.title.x = element_text(size = 16),

axis.title.y = element_text(size = 16))

d <- ggplot(df, aes(x = diameter, y = CD4Treg_in_CD4T)) +

geom_point(size = 3, alpha = 0.7) +

geom_smooth(method = "lm", se = TRUE, color = "blue") +

stat_cor(method = "pearson", label.x = 10, label.y = 75, size = 5,

aes(label = paste(..r.label.., ..p.label.., sep = "~`,`~"))) +

labs(title = "CD4Treg / CD4T",

x = "Tumor Diameter",

y = "Percentage") +

theme_minimal() +

theme(axis.text.x = element_text(hjust = 1,size = 16),

axis.title.x = element_text(size = 16),

axis.title.y = element_text(size = 16))

plot_grid (a, b, c, d, ncol = 4)

# CD8T Shannon

df_Shannon <- dplyr::select(df, c("stage", "size", "CD8T_Shannon"))

df_Shannon$sample <- row.names(df_Shannon)

df_Shannon$sample <- factor(df_Shannon$sample, levels = sample_order)

# Draw a bar plot <<<Figure 3B>>>

ggplot(df_Shannon, aes(x = sample, y = CD8T_Shannon, fill = stage)) +

geom_bar(stat = "identity", position = position_dodge(width = 0.8)) +

labs(title = "Clonal Diversity of CD8T Cells",

x = "Sample", y = "Shannon Index") +

theme_minimal()+

theme(

axis.text.x = element_text(angle = 90, vjust = 0.5, size = 18),

axis.title.x = element_text(angle = 0, hjust = 0.5, size = 18),

axis.title.y = element_text(angle = 90, hjust = 0.5, size = 18),

plot.title = element_text(hjust = 0.5, size = 18)

)

# Correlation between Shannon index and tumor diameter

# <<<Figure 3C>>>

ggplot(df, aes(x = diameter, y = CD8T_Shannon)) +

geom_point() +

geom_smooth(method = "lm", se = TRUE, color = "blue") +

stat_cor(method = "pearson", label.x = min(df$diameter), label.y = max(df$CD8T_Shannon)*0.95) +

geom_text(aes(label = row.names(df)), vjust = -0.5, size = 3) + # NOTE: Comment translated from Japanese (see original script for details).

labs(x = "Diameter", y = "Shannon Index") +

theme_minimal()

# Top20 Ratio Analysis

df_top20 <- dplyr::select(df, c("stage", "size", "top20_ratio"))

df_top20$sample <- row.names(df_top20)

df_top20$sample <- factor(df_top20$sample, levels = sample_order)

# Draw a bar plot <<<Figure S2A>>>

ggplot(df_top20, aes(x = sample, y = top20_ratio, fill = stage)) +

geom_bar(stat = "identity", position = position_dodge(width = 0.8)) +

labs(title = "Occupancy of Top 20 Clones",

x = "Sample", y = "Top 20 %") +

theme_minimal()+

theme(

axis.text.x = element_text(angle = 90, vjust = 0.5, size = 18),

axis.title.x = element_text(angle = 0, hjust = 0.5, size = 18),

axis.title.y = element_text(angle = 90, hjust = 0.5, size = 18),

plot.title = element_text(hjust = 0.5, size = 18)

)

# (Optional) Explicitly set the order of stage levels

df_top20 <- df_top20 %>%

mutate(stage = factor(stage, levels = c("I", "III", "IV")))

# Comparisons to perform

my_comparisons <- list(

c("I", "III"),

c("I", "IV"),

c("III", "IV")

)

p <- ggplot(df_top20, aes(x = stage, y = top20_ratio, fill = stage)) +

geom_boxplot(alpha = 0.8, outlier.shape = NA) +

geom_jitter(width = 0.15, size = 2, alpha = 0.7) +

stat_compare_means(

comparisons = my_comparisons,

method = "t.test",

label = "p.format"

) +

labs(

title = "Occupancy of Top 20 Clones by Stage",

x = "Stage",

y = "Top 20 %"

) +

theme_bw(base_size = 14) +

theme(

axis.text.x = element_text(angle = 0, hjust = 0.5, size = 18),

legend.position = "none",

plot.title = element_text(hjust = 0.5, size = 14) # Center the title

)

p

# <<<Figure S2B>>>

# (Optional) Explicitly set the order of size levels

df_top20 <- df_top20 %>%

mutate(size = factor(size, levels = c("≤7cm", ">7cm")))

# Comparisons to perform

my_comparisons <- list(

c("≤7cm", ">7cm")

)

p <- ggplot(df_top20, aes(x = size, y = top20_ratio, fill = size)) +

geom_boxplot(alpha = 0.8, outlier.shape = NA) +

geom_jitter(width = 0.15, size = 2, alpha = 0.7) +

stat_compare_means(

comparisons = my_comparisons,

method = "t.test",

label = "p.format"

) +

labs(

title = "Occupancy of Top 20 Clones by Tumor Size",

x = "Tumor Diameter",

y = "Top 20 %"

) +

theme_bw(base_size = 14) +

theme(

axis.text.x = element_text(angle = 0, hjust = 0.5, size = 18),

legend.position = "none",

plot.title = element_text(hjust = 0.5, size = 14))

p

# ============================================

# Clonotype Analysis

# ============================================

# Celltype distribution of top20 clones

top20_ids <- paste0("clonotype", 1:20)

KIDall.vdj_R_bys$topclones <- ifelse(

!is.na(KIDall.vdj_R_bys$clonotype_id) &

str_extract(KIDall.vdj_R_bys$clonotype_id, "clonotype\\d+") %in% top20_ids,

"top20",

"other"

)

KIDall.vdj_R_bys$topclones <- factor(KIDall.vdj_R_bys$topclones, levels = c("top20", "other"))

df <- as.data.frame(KIDall.vdj_R_bys@meta.data)

# list of samples in size order

idents <- c('KID001',

'KID002',

'KID018',

'KID012',

'KID016',

'KID013',

'KID006',

'KID015',

'KID011',

'KID014',

'KID004',

'KID007',

'KID005',

'KID009',

'KID010')

# celltype distribution of top20 clones (CD8T only)

top20_ids <- paste0("clonotype", 1:20)

KIDall.vdj_R_bys8$topclones <- ifelse(

!is.na(KIDall.vdj_R_bys8$clonotype_id) &

str_extract(KIDall.vdj_R_bys8$clonotype_id, "clonotype\\d+") %in% top20_ids,

"Top_20",

"Other"

)

KIDall.vdj_R_bys8$topclones <- factor(KIDall.vdj_R_bys8$topclones, levels = c("Top_20", "Other"))

# celltype distribution of top20/other groups

df <- as.data.frame(KIDall.vdj_R_bys8@meta.data)

# List of orig.ident

idents <- c('KID001',

'KID002',

'KID018',

'KID012',

'KID016',

'KID013',

'KID006',

'KID015',

'KID011',

'KID004',

'KID014',

'KID005',

'KID007',

'KID009',

'KID010')

# <<<Figure 3E>>>

# List to store plots

plots <- lapply(idents, function(sid) {

df_sub <- df %>%

dplyr::filter(orig.ident == sid) %>%

dplyr::filter(celltype %in% c('CD8Tnaive',

'CD8Tem',

'CD8Teff',

'CD8Tpex',

'CD8Tex')) %>%

group_by(celltype, topclones) %>%

summarise(n = n(), .groups = "drop") %>%

group_by(topclones) %>%

mutate(Proportion = as.numeric(n) / sum(as.numeric(n))) %>%

ungroup()

ggplot(df_sub, aes(x = celltype, y = Proportion, fill = topclones)) +

geom_bar(stat = "identity", position = position_dodge(width = 0.8)) +

labs(title = sid, x = "Cell Type", y = "Proportion", fill = "Clonotype") +

theme_minimal(base_size = 11) +

theme(

axis.text.x = element_text(angle = 45, hjust = 1),

plot.title = element_text(face = "bold")

)

})

# Arrange multiple plots (e.g., 3 columns)）

wrap_plots(plots, ncol = 3) +

plot_layout(guides = "collect") &

theme(legend.position = "right")

#TIFF export

p <- wrap_plots(plots, ncol = 3) +

plot_layout(guides = "collect") &

theme(legend.position = "right")

ggsave(

filename = "Fig3E.tiff",

plot = p,

device = ragg::agg_tiff,

width = 150, height = 200,

units = "mm",

dpi = 300,

bg = "white"

)

# celltype distribution of reactive clones (CD8T only)

# 8Y/bystanderでグループ分けしてそのcelltype distributionをみる

df <- as.data.frame(KIDall.vdj_rename2_bys_sc8@meta.data)

# orig.identの一覧取得

idents <- c('KID001',

'KID002',

'KID005',

'KID007',

'KID010')

# <<<Figure S8>>>

df <- as.data.frame(KIDall.vdj_rename2_bys_sc8@meta.data)

# list of orig.ident

idents <- c('KID001',

'KID002',

'KID005',

'KID007',

'KID010')

# list to store plots

cell_lv <- c('CD8Tnaive','CD8Tem','CD8Teff','CD8Tpex','CD8Tex')

grp_lv <- c("8Y","Bys")

plots <- lapply(idents, function(sid) {

df_sub <- df %>%

dplyr::filter(orig.ident == sid,

celltype %in% cell_lv,

custom_group %in% grp_lv) %>%

dplyr::count(celltype, custom_group, name = "n") %>%

tidyr::complete(

celltype = cell_lv,

custom_group = grp_lv,

fill = list(n = 0)

) %>%

dplyr::group_by(custom_group) %>%

dplyr::mutate(Proportion = n / sum(n)) %>%

dplyr::ungroup() %>%

dplyr::mutate(

celltype = factor(celltype, levels = cell_lv),

custom_group = factor(custom_group, levels = grp_lv)

)

ggplot(df_sub, aes(x = celltype, y = Proportion, fill = custom_group)) +

geom_col(position = position_dodge2(width = 0.8, preserve = "single"),

width = 0.7) +

scale_x_discrete(drop = FALSE) +

scale_y_continuous(labels = scales::percent_format(accuracy = 1)) +

labs(title = sid, x = "Cell Type", y = "Proportion", fill = "Clonotype")+

scale_fill_discrete(

breaks = c("8Y", "Bys"),

labels = c("Reactive", "Bystander")

) +

theme_minimal(base_size = 11) +

theme(axis.text.x = element_text(angle = 45, hjust = 1, size = 12),

plot.title = element_text(face = "bold", hjust = 0.5),

legend.text = element_text(size = 12))

})

# arrange plot

p <- wrap_plots(plots, ncol = 3) +

plot_layout(guides = "collect") &

theme(legend.position = "right")

p

# TIFF export

ggsave(

filename = "reactivity_celltype_bar.tiff",

plot = p,

width = 8.5,

height = 5.5,

dpi = 300,

units = "in",

device = "tiff",

compression = "lzw"

)

# clonotype visualization <<<Figure 3A>>>

meta_df <- KIDall.vdj_R_bys@meta.data

samples <- c('KID001', 'KID002', 'KID018', 'KID012', 'KID016',

'KID013', 'KID006', 'KID015', 'KID011', 'KID004',

'KID014', 'KID005', 'KID007', 'KID009', 'KID010')

# Calculate top20 clonotypes by sample

top20_list <- setNames(

lapply(samples, function(sid) {

df <- meta_df %>%

dplyr::filter(orig.ident == sid, !is.na(clonotype_id),

percent.mt < 5, nFeature_RNA < 3500) %>%

dplyr::count(clonotype_id, sort = TRUE) %>%

dplyr::slice_max(n, n = 20) %>%

dplyr::mutate(orig.ident = sid)

return(df)

}),

samples

)

top20_all <- bind_rows(top20_list)

# Import expression data for CD4 and CD8A

expr <- FetchData(KIDall.vdj_R_bys, vars = c("CD4", "CD8A", "clonotype_id"))

clone_expr <- expr %>%

filter(!is.na(clonotype_id)) %>%

group_by(clonotype_id) %>%

summarise(

mean_CD4 = mean(CD4, na.rm = TRUE),

mean_CD8A = mean(CD8A, na.rm = TRUE)

) %>%

mutate(

expr_based_group = case_when(

mean_CD4 > mean_CD8A ~ "CD4T",

mean_CD8A > mean_CD4 ~ "CD8T",

TRUE ~ "Other"

)

)

# Merge expression-based clone annotation

top20_all <- left_join(top20_all, clone_expr[, c("clonotype_id", "expr_based_group")],

by = "clonotype_id")

# Calculate total number of clonotype-assigned cells per sample

full_clone_counts <- meta_df %>%

filter(!is.na(clonotype_id)) %>%

count(orig.ident, clonotype_id, name = "n")

total_n_per_sample <- full_clone_counts %>%

group_by(orig.ident) %>%

summarise(total_n = sum(n), .groups = "drop")

# Merge, calculate cumulative proportion, and assign clone rank

top20_all <- top20_all %>%

left_join(total_n_per_sample, by = "orig.ident") %>%

group_by(orig.ident) %>%

arrange(desc(n)) %>%

mutate(

cum_n = cumsum(n),

cum_ratio = cum_n / total_n * 100,

rank = row_number()

) %>%

filter(rank <= 20) %>%

mutate(short_id = paste0("#", rank)) %>%

ungroup()

top20_all$expr_based_group[is.na(top20_all$expr_based_group)] <- "Other"

top20_all$expr_based_group <- factor(

top20_all$expr_based_group,

levels = c("CD8T", "CD4T", "Other")

)

top20_all$short_id <- factor(top20_all$short_id, levels = paste0("#", 1:20))

top20_all$orig.ident <- factor(top20_all$orig.ident, levels = samples)

# Generate plots

plots <- lapply(split(top20_all, top20_all$orig.ident), function(df_sub) {

max_n <- max(df_sub$n, na.rm = TRUE)

dummy_row <- data.frame(

short_id = factor("#1", levels = paste0("#", 1:20)),

n = 0,

cum_ratio = 0,

expr_based_group = factor("Other", levels = c("CD8T", "CD4T", "Other")),

orig.ident = unique(df_sub$orig.ident)

)

df_sub <- df_sub[, c("short_id", "n", "cum_ratio", "expr_based_group", "orig.ident")]

df_sub <- bind_rows(df_sub, dummy_row)

ggplot(df_sub, aes(x = short_id)) +

geom_bar(aes(y = n, fill = expr_based_group), stat = "identity", width = 0.5) +

geom_line(data = df_sub %>% filter(n > 0),

aes(y = cum_ratio * max_n / 100, group = 1),

color = "blue", linewidth = 1) +

scale_y_continuous(

name = "Cell Number",

sec.axis = sec_axis(~ . / max_n * 100, name = "Cumulative Proportion (%)")

) +

scale_x_discrete(name = "Clone Number", breaks = paste0("#", c(1, 6, 11, 16))) +

scale_fill_manual(

values = c("CD8T" = "firebrick", "CD4T" = "forestgreen", "Other" = "gray60"),

drop = FALSE

) +

labs(title = unique(df_sub$orig.ident)) +

theme_minimal() +

theme(axis.text.x = element_text(angle = 0, hjust = 0.5),

legend.position = "none")

})

final_plot <- wrap_plots(plots, ncol = 6)

final_plot

# <<<Figure 3D>>> Top20 overlay

obj <- KIDall.vdj_R_bys

top20_map <- top20_all %>%

transmute(

orig.ident = as.character(orig.ident),

clonotype_id = as.character(clonotype_id),

top20_grp = as.character(expr_based_group) # "CD8T","CD4T","Other"

) %>% distinct()

obj$top20_grp <- "bg"

key_meta <- paste0(as.character(obj$orig.ident), "||", as.character(obj$clonotype_id))

key_map <- paste0(top20_map$orig.ident, "||", top20_map$clonotype_id)

hit <- match(key_meta, key_map)

obj$top20_grp[!is.na(hit)] <- top20_map$top20_grp[hit[!is.na(hit)]]

samples <- c('KID001','KID002','KID018','KID012','KID016',

'KID013','KID006','KID015','KID011','KID004',

'KID014','KID005','KID007','KID009','KID010')

cols_map <- c(bg="grey85", Other="gray40", CD4T="forestgreen", CD8T="firebrick")

draw_order <- c("CD8T","CD4T","Other","bg")

plots <- lapply(samples, function(sid){

DimPlot(

obj, reduction="umap",

cells = WhichCells(obj, expression = orig.ident == sid),

group.by = "top20_grp",

cols = cols_map,

order = draw_order,

shuffle = F,

pt.size = 0.3,

raster = FALSE

) +

ggtitle(sid) +

theme_bw(base_size = 11) +

theme(legend.position = "none",

plot.title = element_text(face = "bold", hjust = 0.5))

})

p<-wrap_plots(plots, ncol = 3)

p

# <<<Figure S7>>> Violin plot with overlaid bars

idents <- c('KID001', 'KID002', 'KID005', 'KID007', 'KID010')

clonoid <- read.csv("clonoid_final.csv")

KIDtopclones <- subset(KIDall.vdj_R_bys, clonotype_id %in% clonoid$clonotype_id)

KIDtopclones$clonotype_id <- factor(

KIDtopclones$clonotype_id,

levels = clonoid$clonotype_id

)

meta <- KIDtopclones@meta.data

meta <- left_join(meta, clonoid[, c("clonotype_id", "clone_number")],

by = "clonotype_id")

meta$clone_number <- as.numeric(gsub("clone", "", meta$clone_number))

KIDtopclones$clone_number <- meta$clone_number

# Create display labels for each clone

KIDtopclones$clone_display <- paste0("#", KIDtopclones$clone_number, "-", KIDtopclones$reactivity)

# Define display order by ascending clone_number

clonoid_order <- KIDtopclones@meta.data %>%

dplyr::select(clone_number, reactivity, clone_display) %>%

distinct() %>%

arrange(as.numeric(clone_number)) %>%

pull(clone_display)

KIDtopclones$clone_display <- factor(KIDtopclones$clone_display, levels = clonoid_order)

Idents(KIDtopclones) <- "clone_display"

vlnplots <- lapply(idents, function(sid) {

VlnPlot(

subset(KIDtopclones, subset = orig.ident == sid),

group.by = "clone_display",

features = c('CD3E',

'CD8A',

'CD4',

'FOXP3',

'TNF',

'IFNG',

'GZMB',

'PRF1',

'CXCL13',

'PDCD1',

'CTLA4',

'HAVCR2',

'LAG3',

'TIGIT',

'TOX',

'NR4A1',

'TGFB1',

'TCF7',

'CCR7'),

stack = TRUE, flip = FALSE

) +

NoLegend() +

ggtitle(sid) +

theme(

axis.text.y = element_blank(),

axis.title.y = element_blank(),

axis.ticks.y = element_blank(),

axis.text.x = element_text(size = 8, face = "plain"),

strip.text.x = element_text(size = 10, face = "plain"),

plot.title = element_text(hjust = 0.5, size = 14),

plot.margin = margin(t = 5.5, r = 5.5, b = 5.5, l = 0)

)

})

## Strip-annotation table

meta_all <- KIDtopclones@meta.data %>% as.data.frame()

df_strip <- meta_all %>%

dplyr::select(orig.ident, clone_display, reactivity) %>%

distinct() %>%

mutate(

ynx = str_sub(reactivity, -1),

ynx_class = case_when(

ynx == "Y" ~ "Y",

ynx == "N" ~ "N",

ynx == "X" ~ "X",

TRUE ~ NA_character_

),

react_prefix = case_when(

str_detect(reactivity, "^4") ~ "4",

str_detect(reactivity, "^8") ~ "8",

str_detect(reactivity, "^DN") ~ "DN",

TRUE ~ NA_character_

)

)

make_strip_plot_y <- function(sid) {

d <- df_strip %>% filter(orig.ident == sid)

d_long <- d %>%

transmute(

clone_display,

ynx_class,

react_prefix

) %>%

pivot_longer(

cols = c("ynx_class", "react_prefix"),

names_to = "strip_type",

values_to = "value"

) %>%

mutate(

strip_type = factor(

strip_type,

levels = c("ynx_class", "react_prefix"),

labels = c("YNX", "react")

)

)

ggplot(d_long, aes(x = strip_type, y = clone_display, fill = value)) +

geom_tile(width = 0.7, height = 0.9,

color = "black",

linewidth = 0.1) +

scale_fill_manual(

values = c(

"Y" = "black",

"N" = "white",

"X" = "gray",

"4" = "darkgreen",

"8" = "firebrick",

"DN" = "gray"

),

na.value = "white",

guide = "none"

) +

scale_x_discrete(expand = c(0, 0)) +

scale_y_discrete(

expand = c(0, 0),

labels = function(x) sub("-.*", "", x)

) +

labs(y = "Clonotype") +

theme_void() +

theme(

axis.text.y = element_text(size = 8),

axis.title.y = element_text(

size = 10,

angle = 90,

margin = margin(r = 4)

),

plot.margin = margin(t = 5.5, r = 0, b = 5.5, l = 8))

}

# Combine VlnPlot + strip

vln_with_strip_y <- map2(vlnplots, idents, function(p, sid) {

p_strip <- make_strip_plot_y(sid)

(p_strip | p) +

plot_layout(widths = c(0.3, 5))

})

# Reorder panels

p1 <- vln_with_strip_y[[1]] # KID001

p2 <- vln_with_strip_y[[2]] # KID002

p3 <- vln_with_strip_y[[3]] # KID005

p4 <- vln_with_strip_y[[4]] # KID007

p5 <- vln_with_strip_y[[5]] # KID010

final_plot <- wrap_plots(p1, p2, p3, p4, p5, ncol = 2)

# Cell-type ratio of top20 clones <<<Figure 5B>>>

meta2 <- KIDtopclones@meta.data

meta2 <- dplyr::select(meta2, c("orig.ident", "celltype", "clone_number", "reactivity"))

meta2$clone_number <- as.character(meta2$clone_number)

meta2$clone_number <- factor(meta2$clone_number, levels = as.character(1:20))

## Cell-type proportion (%)

df_plot <- meta2 %>%

group_by(orig.ident, clone_number, celltype) %>%

summarise(n = n(), .groups = "drop") %>%

group_by(orig.ident, clone_number) %>%

mutate(pct = n / sum(n)) %>%

ungroup()

# Summarize reactivity per clone

df_react <- meta2 %>%

group_by(orig.ident, clone_number) %>%

summarise(

reactivity_raw = dplyr::first(reactivity),

.groups = "drop"

) %>%

mutate(

reactivity_class = case_when(

str_detect(reactivity_raw, "Y") ~ "Y",

str_detect(reactivity_raw, "N") ~ "N",

str_detect(reactivity_raw, "X") ~ "X",

TRUE ~ NA_character_

)

)

## Stacked bar plot

p <- ggplot() +

geom_col(

data = df_plot,

aes(x = clone_number, y = pct, fill = celltype), width = 0.8

) +

facet_wrap(~ orig.ident, scales = "free_x", ncol = 2) +

scale_y_continuous(

labels = percent_format(),

limits = c(-0.1, 1),

expand = c(0, 0)

) +

scale_fill_manual(

values = c(

'CD4Tcm' = "lightgreen",

'CD4Tpex' = "green",

'CD4Tex' = "darkgreen",

'CD4Treg' = "yellow",

'CD8Tnaive' = "orange",

'CD8Tem' = "darkorange",

'CD8Teff' = "pink",

'CD8Tpex' = "salmon",

'CD8Tex' = "red",

'non.T' = "darkgray"

),

name = "Cell Type"

) +

labs(x = "Clonotype (1–20)", y = "Percent within clonotype") +

theme_bw(base_size = 12) +

theme(

axis.text.x = element_text(angle = 0, hjust = 0.5, size = 10.5),

axis.text.y = element_text(size = 12),

axis.title.x = element_text(size = 14),

axis.title.y = element_text(size = 14),

strip.text = element_text(face = "bold", size = 14),

strip.background = element_rect(fill = "white", colour = NA)

) +

ggnewscale::new_scale_fill() +

geom_tile(

data = df_react,

aes(x = clone_number, y = -0.05, fill = reactivity_class),

height = 0.05,

width = 0.8,

color = "black",

linewidth = 0.2

) +

scale_fill_manual(

values = c(

"Y" = "black",

"N" = "white",

"X" = "grey"

),

name = "Reactivity"

)

p

# Box plot of top20 cell-type distribution

KIDall.vdj_R_bys$clone_number <- sub(".*_clonotype", "", KIDall.vdj_R_bys$clonotype_id)

KIDall.vdj_R_bys_top20 <- subset(KIDall.vdj_R_bys, clone_number %in% c(1:20))

allmeta <- KIDall.vdj_R_bys@meta.data

allmeta <- dplyr::select(allmeta, c("orig.ident", "celltype", "clone_number", "reactivity", "custom_group"))

infometa <- read.csv("info.csv")

allmeta <- merge(allmeta, infometa, by = "orig.ident", all = T)

top20meta <- KIDall.vdj_R_bys_top20@meta.data

top20meta <- dplyr::select(top20meta, c("orig.ident", "celltype", "clone_number", "reactivity", "custom_group"))

top20meta <- merge(top20meta, infometa, by = "orig.ident", all = T)

# Cell type vs stage <<<Figure S3>>>

sample_stage <- top20meta %>%

distinct(orig.ident, stage)

celltypes <- c("CD8Tnaive", "CD8Tem", "CD8Teff", "CD8Tpex", "CD8Tex")

all_combo <- crossing(

orig.ident = sample_stage$orig.ident,

celltype = celltypes

) %>%

left_join(sample_stage, by = "orig.ident")

df_counts <- top20meta %>%

count(orig.ident, celltype, stage, name = "n")

df_pct <- all_combo %>%

left_join(df_counts,

by = c("orig.ident", "celltype", "stage")) %>%

mutate(

n = if_else(is.na(n), 0L, n)

) %>%

group_by(orig.ident) %>%

mutate(

pct = n / sum(n) * 100

) %>%

ungroup() %>%

mutate(

stage = factor(stage, levels = c("I", "III", "IV"))

)

plot_list <- map(celltypes, function(ct) {

df_ct <- df_pct %>% filter(celltype == ct)

stages_ct <- sort(unique(df_ct$stage[!is.na(df_ct$stage)]))

comps_all <- if (length(stages_ct) >= 2) {

combn(as.character(stages_ct), 2, simplify = FALSE)

} else {

list()

}

comps_ct <- Filter(function(cp) {

n1 <- sum(df_ct$stage == cp[1] & !is.na(df_ct$pct))

n2 <- sum(df_ct$stage == cp[2] & !is.na(df_ct$pct))

n1 >= 2 && n2 >= 2

}, comps_all)

p <- ggplot(df_ct, aes(x = stage, y = pct, fill = stage)) +

geom_boxplot(outlier.shape = NA, alpha = 0.8) +

geom_jitter(width = 0.15, size = 2, alpha = 0.7) +

scale_y_continuous(expand = expansion(mult = c(0.02, 0.2))) +

labs(

title = paste0(ct, " (Top 20)"),

x = "Stage",

y = paste0("Percent of ", ct, " (%)")

) +

theme_bw(base_size = 12) +

theme(

plot.title = element_text(hjust = 0.5),

legend.position = "none",

axis.text.x = element_text(angle = 0, hjust = 0.5)

)

if (length(comps_ct) > 0) {

p <- p +

stat_compare_means(

comparisons = comps_ct,

method = "wilcox.test",

label = "p.format",

tip.length = 0.01,

size = 4

)

}

p

})

p <- wrap_plots(plot_list, ncol = 3)

p

# celltype vs size <<<Figure S4>>>

sample_size <- top20meta %>%

distinct(orig.ident, size)

all_combo <- crossing(

orig.ident = sample_size$orig.ident,

celltype = celltypes

) %>%

left_join(sample_size, by = "orig.ident")

df_counts <- top20meta %>%

count(orig.ident, celltype, size, name = "n")

df_pct_size <- all_combo %>%

left_join(df_counts,

by = c("orig.ident", "celltype", "size")) %>%

mutate(

n = if_else(is.na(n), 0L, n)

) %>%

group_by(orig.ident) %>%

mutate(

pct = n / sum(n) * 100

) %>%

ungroup() %>%

mutate(

size = factor(size, levels = c("≤7cm", ">7cm"))

)

plot_list_size <- map(celltypes, function(ct) {

df_ct <- df_pct_size %>% filter(celltype == ct)

sizes_ct <- sort(unique(df_ct$size[!is.na(df_ct$size)]))

comps_all <- if (length(sizes_ct) >= 2) {

combn(as.character(sizes_ct), 2, simplify = FALSE)

} else {

list()

}

comps_ct <- Filter(function(cp) {

n1 <- sum(df_ct$size == cp[1] & !is.na(df_ct$pct))

n2 <- sum(df_ct$size == cp[2] & !is.na(df_ct$pct))

n1 >= 2 && n2 >= 2

}, comps_all)

p <- ggplot(df_ct, aes(x = size, y = pct, fill = size)) +

geom_boxplot(outlier.shape = NA, alpha = 0.8) +

geom_jitter(width = 0.15, size = 2, alpha = 0.7) +

scale_y_continuous(expand = expansion(mult = c(0.02, 0.2))) +

labs(

title = paste0(ct, " (Top 20)"),

x = "Tumor Diameter",

y = paste0("Percent of ", ct, " (%)")

) +

theme_bw(base_size = 12) +

theme(

plot.title = element_text(hjust = 0.5),

legend.position = "none",

axis.text.x = element_text(angle = 0, hjust = 0.5)

)

if (length(comps_ct) > 0) {

p <- p +

stat_compare_means(

comparisons = comps_ct,

method = "wilcox.test",

label = "p.format",

tip.length = 0.01,

size = 3

)

}

p

})

p <- wrap_plots(plot_list_size, ncol = 3)

p

# ============================================

# Machine Learning for Prediction of Tumor-Reactive T Cells

# ============================================

split_stratified_barcodes_by_combo <- function(seu, test_frac = 0.2, seed = 123) {

set.seed(seed)

meta <- seu@meta.data

combo <- with(meta, paste(orig.ident, custom_group, sep = "_"))

valid_cells <- which(!is.na(combo) & !grepl("NA", combo))

combo_factor <- factor(combo[valid_cells])

test_idx <- caret::createDataPartition(combo_factor, p = test_frac, list = FALSE)

test_barcodes <- rownames(meta)[valid_cells][test_idx]

train_barcodes <- setdiff(colnames(seu), test_barcodes)

return(list(train = train_barcodes, test = test_barcodes))

}

barcode_split <- split_stratified_barcodes_by_combo(KIDall.vdj_R_bys8, test_frac = 0.2)

train_barcodes <- barcode_split$train

test_barcodes <- barcode_split$test

KIDall.vdj_R_bys8_train <- subset(KIDall.vdj_R_bys8, cells = train_barcodes)

KIDall.vdj_R_bys8_test <- subset(KIDall.vdj_R_bys8, cells = test_barcodes)

# Extract DEGs

Idents(KIDall.vdj_R_bys8_train) <- "custom_group"

DEG <- FindMarkers(KIDall.vdj_R_bys8_train, ident.1 = "8Y", ident.2 = "Bys")

fDEG <- DEG %>%

dplyr::filter(

p_val < 0.05,

p_val_adj < 0.05,

abs(avg_log2FC) > 1

) %>%

.[!grepl("^TRAV|^TRBV", rownames(.)), ]

DEGs <- rownames(fDEG)

# <<<Figure 6B>>>

DEG$gene <- rownames(DEG)

DEG <- DEG %>%

mutate(

log10_pval = -log10(p_val_adj + 1e-300),

significance = case_when(

p_val_adj < 0.05 & avg_log2FC > 1 ~ "Upregulated",

p_val_adj < 0.05 & avg_log2FC < -1 ~ "Downregulated",

TRUE ~ "Not significant"

)

) %>%

.[!grepl("^TRAV|^TRBV", rownames(.)), ]

top_genes <- DEG %>%

filter(p_val_adj < 0.05, abs(avg_log2FC) > 1) %>%

arrange(p_val_adj) %>%

slice_head(n = 30)

ggplot(DEG, aes(x = avg_log2FC, y = log10_pval)) +

geom_point(aes(color = significance), alpha = 0.8) +

geom_text_repel(data = top_genes, aes(label = gene), size = 4) +

scale_color_manual(

values = c(

"Upregulated" = "red",

"Downregulated" = "blue",

"Not significant" = "grey"

)

) +

geom_vline(xintercept = c(-1, 1), linetype = "dashed", color = "black") +

geom_hline(yintercept = -log10(0.05), linetype = "dashed", color = "black") +

labs(x = "avg_log2FC", y = "-log10(adj p-value)") +

theme_minimal()

# Prepare data for machine learning

expr_mat <- t(GetAssayData(KIDall.vdj_R_bys8_train, slot = "data")[DEGs, ])

df_rf <- as.data.frame(expr_mat)

colnames(df_rf) <- make.names(colnames(df_rf))

df_rf$label <- KIDall.vdj_R_bys8_train$custom_group

df_rf$orig.ident <- KIDall.vdj_R_bys8_train$orig.ident

df_rf_subset <- subset(df_rf, label %in% c("8Y", "Bys") & !is.na(label))

df_rf_subset$label <- factor(df_rf_subset$label, levels = c("8Y", "Bys"), labels = c("Y8", "Bys"))

df_rf_subset$orig.ident <- factor(df_rf_subset$orig.ident)

trainData <- df_rf_subset[, names(df_rf_subset) != "orig.ident"]

# Cross-validation settings

control <- trainControl(

method = "cv",

number = 5,

classProbs = TRUE,

summaryFunction = twoClassSummary,

savePredictions = "final"

)

set.seed(123)

# Train models

rf_model_cv <- train(

label ~ .,

data = trainData,

method = "rf",

trControl = control,

metric = "ROC",

tuneGrid = expand.grid(mtry = 1:min(10, ncol(trainData) - 1)),

importance = TRUE

)

glmnet_model_cv <- train(

label ~ .,

data = trainData,

method = "glmnet",

trControl = control,

metric = "ROC",

preProcess = c("center", "scale")

)

svm_model_cv <- train(

label ~ .,

data = trainData,

method = "svmRadial",

trControl = control,

metric = "ROC",

preProcess = c("center", "scale"),

tuneLength = 5

)

gbm_model_cv <- train(

label ~ .,

data = trainData,

method = "gbm",

trControl = control,

metric = "ROC",

verbose = FALSE,

tuneLength = 10

)

# Model comparison

resamps <- resamples(list(

RF = rf_model_cv,

PLR = glmnet_model_cv,

SVM = svm_model_cv,

GBM = gbm_model_cv

))

# <<<Figure 6C>>>

roc_long <- resamps$values %>%

dplyr::select(Resample, contains("ROC")) %>%

pivot_longer(

cols = -Resample,

names_to = "model",

values_to = "ROC"

) %>%

mutate(

model = sub("~ROC", "", model),

model = factor(model, levels = c("RF", "PLR", "SVM", "GBM"))

)

mods <- levels(roc_long$model)

all_pairs <- combn(mods, 2, simplify = FALSE)

ggplot(roc_long, aes(x = model, y = ROC, fill = model)) +

geom_boxplot(alpha = 0.8, outlier.shape = NA) +

geom_jitter(width = 0.1, alpha = 0.6, size = 1) +

stat_compare_means(

comparisons = all_pairs,

method = "t.test",

p.adjust.method = "bonferroni",

label = "p.format",

size = 3

) +

labs(title = "Cross-validation AUC by model", x = "Model", y = "AUC") +

theme_bw(base_size = 12) +

theme(legend.position = "none", plot.title = element_text(hjust = 0.5))

# Top 10 positive/negative coefficients for PLR

best <- glmnet_model_cv$bestTune

beta <- as.matrix(coef(glmnet_model_cv$finalModel, s = best$lambda))

coef_df <- data.frame(

gene = rownames(beta),

coef = as.numeric(beta)

) %>%

filter(gene != "(Intercept)", coef != 0)

coef_df$gene <- gsub("\\_", ".", coef_df$gene)

coef_df$coef <- -coef_df$coef

top_pos10 <- coef_df %>%

arrange(desc(coef)) %>%

slice_head(n = 10) %>%

mutate(group = "Top10_positive")

top_neg10 <- coef_df %>%

arrange(coef) %>%

slice_head(n = 10) %>%

mutate(group = "Top10_negative")

coef_tb20 <- bind_rows(top_pos10, top_neg10)

# <<<Figure 6E>>>

ggplot(coef_tb20, aes(x = reorder(gene, coef), y = coef, fill = group)) +

geom_bar(stat = "identity") +

coord_flip() +

scale_fill_manual(

values = c("Top10_positive" = "#F8766D", "Top10_negative" = "#00BFC4")

) +

labs(title = "Top 10 Positive/Negative Coefficients (PLR)",

x = "Gene", y = "Coefficient", fill = "") +

theme_minimal()

# Test model performance

expr_mat_test <- t(GetAssayData(KIDall.vdj_R_bys8_test, slot = "data")[DEGs, ])

df_test <- as.data.frame(expr_mat_test)

colnames(df_test) <- make.names(colnames(df_test))

df_test$label <- KIDall.vdj_R_bys8_test$custom_group

df_test$orig.ident <- KIDall.vdj_R_bys8_test$orig.ident

df_test_subset <- subset(df_test, label %in% c("8Y", "Bys") & !is.na(label))

df_test_subset$label <- factor(df_test_subset$label, levels = c("8Y", "Bys"), labels = c("Y8", "Bys"))

testData <- df_test_subset[, names(df_test_subset) != "orig.ident"]

# <<<Figure 6D>>>

eval_model_roc_cv <- function(model, testData, positive = "Y8", main_title = "Model") {

pred <- model$pred

bt <- model$bestTune

for (nm in names(bt)) {

pred <- pred[pred[[nm]] == bt[[nm]], ]

}

roc_cv <- roc(

response = pred$obs,

predictor = pred[[positive]],

levels = c("Bys", positive)

)

prob_test <- predict(model, newdata = testData, type = "prob")

roc_test <- roc(

response = testData$label,

predictor = prob_test[[positive]],

levels = c("Bys", positive)

)

plot(roc_cv, col = "#1f77b4", lwd = 2, main = paste0("ROC Curve for ", main_title, " Model"))

plot(roc_test, col = "#d62728", lwd = 2, add = TRUE)

abline(a = 0, b = 1, lty = 2, col = "gray50")

legend("bottom",

legend = c(

paste0("Training AUC = ", sprintf("%.3f", auc(roc_cv))),

paste0("Test AUC = ", sprintf("%.3f", auc(roc_test)))

),

col = c("#1f77b4", "#d62728"),

lwd = 2, bty = "n", cex = 1, y.intersp = 1.2)

invisible(list(cv = roc_cv, test = roc_test))

}

eval_model_roc_cv(glmnet_model_cv, testData, main_title = "PLR")

# ============================================

# Validation Using Public Dataset (Meng_PDAC)

# ============================================

# Analysis of public PDAC data by Meng et al.

mat <- data.table::fread(file = "GSE254250_PDAC_9sample_CD8_RNA_expression_matrix.csv.gz")

mat <- data.frame(mat, row.names = 1)

mat[is.na(mat)] <- 0

TIL <- CreateSeuratObject(mat)

metadata <- read.csv("GSE254250_PDAC_9sample_CD8_RNA_metadata_matrix.csv.gz")

rownames(metadata) <- metadata$X

metadata <- metadata[, -1]

TIL <- AddMetaData(TIL, metadata)

TIL <- NormalizeData(TIL)

TIL$custom_group <- "NA"

TIL$custom_group[TIL$Reactivity..n.118. == "TR"] <- "8Y"

TIL$custom_group[TIL$Reactivity..n.118. == "NTR"] <- "Bys"

TIL$custom_group[TIL$Reactivity..n.149. == "TR"] <- "8Y"

TIL$custom_group[TIL$Reactivity..n.149. == "NTR"] <- "Bys"

# Apply model to PDAC data

available_genes <- rownames(GetAssayData(TIL, layer = "data"))

common_genes <- intersect(DEGs, available_genes)

gene_order <- colnames(trainData)[colnames(trainData) != "label"]

valid_genes <- intersect(gene_order, rownames(GetAssayData(TIL, layer = "data")))

expr_mat_test <- t(GetAssayData(TIL, layer = "data")[valid_genes, ])

df_test <- as.data.frame(expr_mat_test)

missing_genes <- setdiff(gene_order, colnames(df_test))

for (gene in missing_genes) {

df_test[[gene]] <- 0

}

df_test <- df_test[, gene_order]

df_test$label <- TIL$custom_group

df_test <- subset(df_test, label %in% c("8Y", "Bys") & !is.na(label))

df_test$label <- factor(df_test$label, levels = c("8Y", "Bys"), labels = c("Y8", "Bys"))

testData <- df_test

# Class prediction (glmnet)

pred_class <- predict(glmnet_model_cv, newdata = testData)

pred_prob <- predict(glmnet_model_cv, newdata = testData, type = "prob")

true_labels <- testData$label

roc_obj <- roc(response = true_labels,

predictor = pred_prob[, "Y8"],

levels = levels(true_labels))

# <<<Figure 6F>>>

plot(roc_obj, main = "ROC Curve for PLR Model (PDAC Data)")

abline(a = 0, b = 1, lty = 2, col = "gray")

auc_value <- auc(roc_obj)

text(x = 0.4, y = 0.2, labels = paste0("AUC = ", sprintf("%.3f", auc_value)))

# ============================================

# TR Score ROC Analysis

# ============================================

# Scoring TR score on ccRCC data using UCell

TR_list <- list(c('TNFRSF9',

'VCAM1',

'TIGIT',

'HAVCR2',

'GZMB',

'ACP5',

'NKG7',

'KRT86',

'LAYN',

'HLA-DRB5',

'CTLA4',

'HLA-DRB1',

'IGFLR1',

'HLA-DRA',

'LAG3',

'GEM',

'CXCL13',

'LYST',

'GAPDH',

'CD74',

'HMOX1',

'HLA-DPA1',

'DUSP4',

'CD27',

'ENTPD1',

'AC243829.4',

'HLA-DPB1',

'GZMH',

'KIR2DL4',

'CARD16'))

gene_sets <- list(TR_Score = TR_list)

KIDall.vdj_R_bys <- AddModuleScore_UCell(KIDall.vdj_R_bys, features = gene_sets)

df <- KIDall.vdj_R_bys@meta.data %>%

dplyr::filter(custom_group %in% c("8Y", "Bys"))

df$label <- ifelse(df$custom_group == "8Y", 1, 0)

roc_result3 <- pROC::roc(response = df$label, predictor = df$TR_Score_UCell)

# <<<Figure 6A>>>

plot(roc_result3, main = "ROC Curve for TR Score")

abline(a = 0, b = 1, lty = 2, col = "gray")

auc_value <- auc(roc_result3)

text(x = 0.4, y = 0.2, labels = paste0("AUC = ", sprintf("%.3f", auc_value)))

# ============================================

# Prediction Model Construction on Public PDAC Data

# ============================================

split_stratified_barcodes_by_combo <- function(seu, test_frac = 0.2, seed = 123) {

set.seed(seed)

meta <- seu@meta.data

combo <- with(meta, paste(orig.ident, custom_group, sep = "_"))

valid_cells <- which(!is.na(combo) & !grepl("NA", combo))

combo_factor <- factor(combo[valid_cells])

test_idx <- caret::createDataPartition(combo_factor, p = test_frac, list = FALSE)

train_idx <- setdiff(seq_along(combo_factor), test_idx)

test_barcodes <- rownames(meta)[valid_cells][test_idx]

train_barcodes <- rownames(meta)[valid_cells][train_idx]

return(list(train = train_barcodes, test = test_barcodes))

}

barcode_split <- split_stratified_barcodes_by_combo(TIL, test_frac = 0.2)

train_barcodes <- barcode_split$train

test_barcodes <- barcode_split$test

TIL_train <- subset(TIL, cells = train_barcodes)

TIL_test <- subset(TIL, cells = test_barcodes)

# Extract DEGs from PDAC data

Idents(TIL_train) <- "custom_group"

DEG <- FindMarkers(TIL_train, ident.1 = "8Y", ident.2 = "Bys")

fDEG <- DEG %>%

dplyr::filter(

p_val < 0.05,

p_val_adj < 0.05,

abs(avg_log2FC) > 1

) %>%

.[!grepl("^TRAV|^TRBV", rownames(.)), ]

DEGs <- rownames(fDEG)

# <<<Figure 6G>>>

DEG$gene <- rownames(DEG)

DEG <- DEG %>%

mutate(

log10_pval = -log10(p_val_adj + 1e-300),

significance = case_when(

p_val_adj < 0.05 & avg_log2FC > 1 ~ "Upregulated",

p_val_adj < 0.05 & avg_log2FC < -1 ~ "Downregulated",

TRUE ~ "Not significant"

)

) %>%

.[!grepl("^TRAV|^TRBV", rownames(.)), ]

top_genes <- DEG %>%

filter(p_val_adj < 0.05, abs(avg_log2FC) > 1) %>%

arrange(p_val_adj) %>%

slice_head(n = 30)

ggplot(DEG, aes(x = avg_log2FC, y = log10_pval)) +

geom_point(aes(color = significance), alpha = 0.8) +

geom_text_repel(data = top_genes, aes(label = gene), size = 4) +

scale_color_manual(

values = c(

"Upregulated" = "red",

"Downregulated" = "blue",

"Not significant" = "grey"

)

) +

geom_vline(xintercept = c(-1, 1), linetype = "dashed", color = "black") +

geom_hline(yintercept = -log10(0.05), linetype = "dashed", color = "black") +

labs(x = "avg_log2FC", y = "-log10(adj p-value)") +

theme_minimal()

# Prepare PDAC data for machine learning

expr_mat <- t(GetAssayData(TIL_train, slot = "data")[DEGs, ])

df_rf <- as.data.frame(expr_mat)

colnames(df_rf) <- make.names(colnames(df_rf))

df_rf$label <- TIL_train$custom_group

df_rf$orig.ident <- TIL_train$orig.ident

df_rf_subset <- subset(df_rf, label %in% c("8Y", "Bys") & !is.na(label))

df_rf_subset$label <- factor(df_rf_subset$label, levels = c("8Y", "Bys"), labels = c("Y8", "Bys"))

df_rf_subset$orig.ident <- factor(df_rf_subset$orig.ident)

trainData <- df_rf_subset[, names(df_rf_subset) != "orig.ident"]

# Train models on PDAC data

rf_model_cv <- train(

label ~ .,

data = trainData,

method = "rf",

trControl = control,

metric = "ROC",

tuneGrid = expand.grid(mtry = 1:min(10, ncol(trainData) - 1)),

importance = TRUE

)

glmnet_model_cv <- train(

label ~ .,

data = trainData,

method = "glmnet",

trControl = control,

metric = "ROC",

preProcess = c("center", "scale")

)

svm_model_cv <- train(

label ~ .,

data = trainData,

method = "svmRadial",

trControl = control,

metric = "ROC",

preProcess = c("center", "scale"),

tuneLength = 5

)

gbm_model_cv <- train(

label ~ .,

data = trainData,

method = "gbm",

trControl = control,

metric = "ROC",

verbose = FALSE,

tuneLength = 10

)

# Shared DEGs between ccRCC and PDAC

ccRCCDEG <- read.csv(file = "I:/R/Seurat/KID12456791011121314151618VDJ/fDEG_with_coef.csv")

colnames(ccRCCDEG) <- paste0("ccRCC_", colnames(ccRCCDEG))

colnames(fDEGwCoef) <- paste0("PDAC_", colnames(fDEGwCoef))

sharedDEG <- merge(

ccRCCDEG,

fDEGwCoef,

by.x = "ccRCC_gene",

by.y = "PDAC_gene",

all = FALSE

)

write.csv(sharedDEG, file = "sharedDEG.csv")

# Top 10 positive/negative coefficients for PLR (PDAC)

best <- glmnet_model_cv$bestTune

beta <- as.matrix(coef(glmnet_model_cv$finalModel, s = best$lambda))

coef_df <- data.frame(

gene = rownames(beta),

coef = as.numeric(beta)

) %>%

filter(gene != "(Intercept)", coef != 0)

coef_df$gene <- gsub("\\_", ".", coef_df$gene)

coef_df$coef <- -coef_df$coef

top_pos10 <- coef_df %>%

arrange(desc(coef)) %>%

slice_head(n = 10) %>%

mutate(group = "Top10_positive")

top_neg10 <- coef_df %>%

arrange(coef) %>%

slice_head(n = 10) %>%

mutate(group = "Top10_negative")

coef_tb20 <- bind_rows(top_pos10, top_neg10)

# <<<Figure 6I>>>

ggplot(coef_tb20, aes(x = reorder(gene, coef), y = coef, fill = group)) +

geom_bar(stat = "identity") +

coord_flip() +

scale_fill_manual(

values = c("Top10_positive" = "#F8766D", "Top10_negative" = "#00BFC4")

) +

labs(title = "Top 10 Positive / Negative Coefficients (PLR)",

x = "Gene", y = "Coefficient", fill = "") +

theme_minimal()

# Test PDAC model performance

expr_mat_test <- t(GetAssayData(TIL_test, slot = "data")[DEGs, ])

df_test <- as.data.frame(expr_mat_test)

colnames(df_test) <- make.names(colnames(df_test))

df_test$label <- TIL_test$custom_group

df_test$orig.ident <- TIL_test$orig.ident

df_test_subset <- subset(df_test, label %in% c("8Y", "Bys") & !is.na(label))

df_test_subset$label <- factor(df_test_subset$label, levels = c("8Y", "Bys"), labels = c("Y8", "Bys"))

testData <- df_test_subset[, names(df_test_subset) != "orig.ident"]

# <<<Figure 6H>>>

eval_model_roc_cv(glmnet_model_cv, testData, main_title = "PLR")

# ============================================

# Model Application on RCC Data

# ============================================

# Apply PDAC-trained model to RCC data

available_genes <- rownames(GetAssayData(KIDall.vdj_rename2_bys_sc_F8, layer = "data"))

common_genes <- intersect(DEGs, available_genes)

gene_order <- colnames(trainData)[colnames(trainData) != "label"]

valid_genes <- intersect(gene_order, rownames(GetAssayData(KIDall.vdj_rename2_bys_sc_F8, layer = "data")))

expr_mat_test <- t(GetAssayData(KIDall.vdj_rename2_bys_sc_F8, layer = "data")[valid_genes, ])

df_test <- as.data.frame(expr_mat_test)

missing_genes <- setdiff(gene_order, colnames(df_test))

for (gene in missing_genes) {

df_test[[gene]] <- 0

}

df_test <- df_test[, gene_order]

df_test$label <- KIDall.vdj_rename2_bys_sc_F8$custom_group

df_test <- subset(df_test, label %in% c("8Y", "Bys") & !is.na(label))

df_test$label <- factor(df_test$label, levels = c("8Y", "Bys"), labels = c("Y8", "Bys"))

testData <- df_test

# Test GBM model on RCC data

pred_class <- predict(gbm_model_cv, newdata = testData)

pred_prob <- predict(gbm_model_cv, newdata = testData, type = "prob")

true_labels <- testData$label

roc_obj <- roc(response = true_labels,

predictor = pred_prob[, "Y8"],

levels = levels(true_labels))

# <<<Figure 6J>>>

plot(roc_obj, main = "ROC Curve for GBM Model (RCC Data)")

abline(a = 0, b = 1, lty = 2, col = "gray")

auc_value <- auc(roc_obj)

text(x = 0.6, y = 0.2, labels = paste0("AUC = ", sprintf("%.3f", auc_value)))

# =========<<<<sessionInfo()>>>>======================

R version 4.5.1 (2025-06-13 ucrt)

Platform: x86_64-w64-mingw32/x64

Running under: Windows 11 x64 (build 26200)

Matrix products: default

LAPACK version 3.12.1

locale:

[1] LC_COLLATE=Japanese_Japan.utf8 LC_CTYPE=Japanese_Japan.utf8 LC_MONETARY=Japanese_Japan.utf8

[4] LC_NUMERIC=C LC_TIME=Japanese_Japan.utf8

time zone: Asia/Tokyo

tzcode source: internal

attached base packages:

[1] stats4 grid stats graphics grDevices utils datasets methods base

other attached packages:

[1] UCell_2.14.0 scales_1.4.0 ggnewscale_0.5.2

[4] ggtext_0.1.2 ggpubr_0.6.2 gbm_2.2.2

[7] caret_7.0-1 lattice_0.22-7 pROC_1.19.0.1

[10] Matrix_1.7-4 MASS_7.3-65 ggthemes_5.1.0

[13] scRepertoire_2.5.8 SingleCellExperiment_1.32.0 celldex_1.20.0

[16] SingleR_2.12.0 SummarizedExperiment_1.40.0 Biobase_2.70.0

[19] GenomicRanges_1.62.0 Seqinfo_1.0.0 IRanges_2.44.0

[22] S4Vectors_0.48.0 BiocGenerics_0.56.0 generics_0.1.4

[25] MatrixGenerics_1.22.0 matrixStats_1.5.0 viridis_0.6.5

[28] viridisLite_0.4.2 RColorBrewer_1.1-3 pheatmap_1.0.13

[31] VennDiagram_1.7.3 futile.logger_1.4.3 ggrepel_0.9.6

[34] patchwork_1.3.2 lubridate_1.9.4 forcats_1.0.1

[37] stringr_1.6.0 purrr_1.2.0 readr_2.1.5

[40] tidyr_1.3.1 tibble_3.3.0 ggplot2_4.0.0

[43] tidyverse_2.0.0 dplyr_1.1.4 Seurat_5.3.1

[46] SeuratObject_5.2.0 sp_2.2-0

loaded via a namespace (and not attached):

[1] spatstat.sparse_3.1-0 httr_1.4.7 backports_1.5.0

[4] tools_4.5.1 sctransform_0.4.2 alabaster.base_1.10.0

[7] R6_2.6.1 HDF5Array_1.38.0 lazyeval_0.2.2

[10] uwot_0.2.4 rhdf5filters_1.22.0 withr_3.0.2

[13] gridExtra_2.3 progressr_0.18.0 quantreg_6.1

[16] cli_3.6.5 formatR_1.14 spatstat.explore_3.5-3

[19] fastDummies_1.7.5 iNEXT_3.0.2 alabaster.se_1.10.0

[22] S7_0.2.0 spatstat.data_3.1-9 ggridges_0.5.7

[25] pbapply_1.7-4 dichromat_2.0-0.1 parallelly_1.45.1

[28] rstudioapi_0.17.1 RSQLite_2.4.5 ica_1.0-3

[31] spatstat.random_3.4-2 car_3.1-3 abind_1.4-8

[34] lifecycle_1.0.4 yaml_2.3.11 carData_3.0-5

[37] recipes_1.3.1 rhdf5_2.54.1 SparseArray_1.10.6

[40] BiocFileCache_3.0.0 Rtsne_0.17 blob_1.2.4

[43] promises_1.5.0 ExperimentHub_3.0.0 crayon_1.5.3

[46] miniUI_0.1.2 beachmat_2.26.0 cowplot_1.2.0

[49] chromote_0.5.1 KEGGREST_1.50.0 pillar_1.11.1

[52] rjson_0.2.23 future.apply_1.20.1 codetools_0.2-20

[55] glue_1.8.0 spatstat.univar_3.1-4 data.table_1.17.8

[58] vctrs_0.6.5 png_0.1-8 gypsum_1.6.0

[61] spam_2.11-1 gtable_0.3.6 cachem_1.1.0

[64] gower_1.0.2 prodlim_2025.04.28 S4Arrays_1.10.1

[67] mime_0.13 tidygraph_1.3.1 survival_3.8-3

[70] timeDate_4051.111 iterators_1.0.14 hardhat_1.4.2

[73] lava_1.8.2 fitdistrplus_1.2-4 ipred_0.9-15

[76] ROCR_1.0-11 nlme_3.1-168 bit64_4.6.0-1

[79] alabaster.ranges_1.10.0 filelock_1.0.3 RcppAnnoy_0.0.22

[82] irlba_2.3.5.1 rpart_4.1.24 KernSmooth_2.23-26

[85] otel_0.2.0 DBI_1.2.3 nnet_7.3-20

[88] processx_3.8.6 tidyselect_1.2.1 bit_4.6.0

[91] compiler_4.5.1 curl_7.0.0 rvest_1.0.5

[94] httr2_1.2.2 BiocNeighbors_2.4.0 h5mread_1.2.1

[97] SparseM_1.84-2 xml2_1.5.1 ggdendro_0.2.0

[100] DelayedArray_0.36.0 plotly_4.11.0 lmtest_0.9-40

[103] rappdirs_0.3.3 digest_0.6.38 goftest_1.2-3

[106] spatstat.utils_3.2-0 alabaster.matrix_1.10.0 XVector_0.50.0

[109] htmltools_0.5.8.1 pkgconfig_2.0.3 sparseMatrixStats_1.22.0

[112] dbplyr_2.5.1 fastmap_1.2.0 rlang_1.1.6

[115] htmlwidgets_1.6.4 shiny_1.12.1 DelayedMatrixStats_1.32.0

[118] immApex_1.4.2 farver_2.1.2 zoo_1.8-14

[121] jsonlite_2.0.0 BiocParallel_1.44.0 ModelMetrics_1.2.2.2

[124] magrittr_2.0.4 Formula_1.2-5 dotCall64_1.2

[127] Rhdf5lib_1.32.0 Rcpp_1.1.0 evmix_2.12

[130] reticulate_1.44.0 stringi_1.8.7 alabaster.schemas_1.10.0

[133] ggalluvial_0.12.5 ggraph_2.2.2 AnnotationHub_4.0.0

[136] plyr_1.8.9 parallel_4.5.1 listenv_0.10.0

[139] deldir_2.0-4 Biostrings_2.78.0 graphlayouts_1.2.2

[142] splines_4.5.1 gridtext_0.1.5 tensor_1.5.1

[145] hash_2.2.6.3 hms_1.1.4 ps_1.9.1

[148] igraph_2.2.1 spatstat.geom_3.6-0 ggsignif_0.6.4

[151] RcppHNSW_0.6.0 reshape2_1.4.5 futile.options_1.0.1

[154] BiocVersion_3.22.0 lambda.r_1.2.4 BiocManager_1.30.26

[157] foreach_1.5.2 tzdb_0.5.0 tweenr_2.0.3

[160] httpuv_1.6.16 MatrixModels_0.5-4 RANN_2.6.2

[163] polyclip_1.10-7 future_1.68.0 scattermore_1.2

[166] ggforce_0.5.0 broom_1.0.11 xtable_1.8-4

[169] RSpectra_0.16-2 rstatix_0.7.3 later_1.4.4

[172] class_7.3-23 gsl_2.1-9 websocket_1.4.4

[175] memoise_2.0.1 AnnotationDbi_1.72.0 cluster_2.1.8.1

[178] timechange_0.3.0 globals_0.18.0
