# Supplementary material for: Tumor-reactive TCRs within exhausted TILs reveal cancer type-specific immune landscapes in renal cell carcinoma
Source: Front Immunol. 2026 Jan 29;17:1729388. doi: 10.3389/fimmu.2026.1729388 (PMC12894369; doi:10.3389/fimmu.2026.1729388)
Supplement: Supplementary file 2 [file DataSheet2.docx]

## Supplementary text 2

## R script for analysis of bulk RNA sequence data

############################################################

## Load required libraries

############################################################

library(Seurat)

library(dplyr)

library(tidyverse)

library(purrr)

library(readr)

library(stringr)

library(tidyr)

library(ggplot2)

library(ggpubr)

# ============================================

# Part 1. Prepare ccRCC scRNA-seq data

# (public datasets, processed identically)

# ============================================

# First, prepare expression matrix for custom CIBERSORTx run using publically availeble ccRCC scRNAseq data.

setwd("path/to/a folder containing public RCC scRNA data in sub-folders with sample names)

dir <- "RCC101"

TIL.data <- Read10X(data.dir = dir)

TIL <- CreateSeuratObject(counts = TIL.data, project = dir, min.cells = 3, min.features = 200,names.field = 2)

TIL[['percent.mt']] <- PercentageFeatureSet(TIL, pattern = '^MT-')

VlnPlot(TIL, features = c('nFeature_RNA', 'nCount_RNA', 'percent.mt'), ncol = 3)

TIL1 <- subset(TIL, subset = nFeature_RNA > 200 & nFeature_RNA < 2000 & percent.mt < 8)

dir <- "RCC113"

TIL.data <- Read10X(data.dir = dir)

TIL <- CreateSeuratObject(counts = TIL.data, project = dir, min.cells = 3, min.features = 200,names.field = 2)

TIL[['percent.mt']] <- PercentageFeatureSet(TIL, pattern = '^MT-')

VlnPlot(TIL, features = c('nFeature_RNA', 'nCount_RNA', 'percent.mt'), ncol = 3)

TIL2 <- subset(TIL, subset = nFeature_RNA > 200 & nFeature_RNA < 2000 & percent.mt < 8)

dir <- "RCC96"

TIL.data <- Read10X(data.dir = dir)

TIL <- CreateSeuratObject(counts = TIL.data, project = dir, min.cells = 3, min.features = 200,names.field = 2)

TIL[['percent.mt']] <- PercentageFeatureSet(TIL, pattern = '^MT-')

VlnPlot(TIL, features = c('nFeature_RNA', 'nCount_RNA', 'percent.mt'), ncol = 3)

TIL3 <- subset(TIL, subset = nFeature_RNA > 200 & nFeature_RNA < 2000 & percent.mt < 8)

dir <- "RCC100"

TIL.data <- Read10X(data.dir = dir)

TIL <- CreateSeuratObject(counts = TIL.data, project = dir, min.cells = 3, min.features = 200,names.field = 2)

TIL[['percent.mt']] <- PercentageFeatureSet(TIL, pattern = '^MT-')

VlnPlot(TIL, features = c('nFeature_RNA', 'nCount_RNA', 'percent.mt'), ncol = 3)

TIL4 <- subset(TIL, subset = nFeature_RNA > 200 & nFeature_RNA < 2000 & percent.mt < 8)

dir <- "RCC87"

TIL.data <- Read10X(data.dir = dir)

TIL <- CreateSeuratObject(counts = TIL.data, project = dir, min.cells = 3, min.features = 200,names.field = 2)

TIL[['percent.mt']] <- PercentageFeatureSet(TIL, pattern = '^MT-')

VlnPlot(TIL, features = c('nFeature_RNA', 'nCount_RNA', 'percent.mt'), ncol = 3)

TIL5 <- subset(TIL, subset = nFeature_RNA > 200 & nFeature_RNA < 2000 & percent.mt < 8)

dir <- "RCC104"

TIL.data <- Read10X(data.dir = dir)

TIL <- CreateSeuratObject(counts = TIL.data, project = dir, min.cells = 3, min.features = 200,names.field = 2)

TIL[['percent.mt']] <- PercentageFeatureSet(TIL, pattern = '^MT-')

VlnPlot(TIL, features = c('nFeature_RNA', 'nCount_RNA', 'percent.mt'), ncol = 3)

TIL6 <- subset(TIL, subset = nFeature_RNA > 200 & nFeature_RNA < 2000 & percent.mt < 8)

# ============================================

# Part 2. Normalization and merging

# ============================================

TIL1 <- NormalizeData(TIL1)

TIL2 <- NormalizeData(TIL2)

TIL3 <- NormalizeData(TIL3)

TIL4 <- NormalizeData(TIL4)

TIL5 <- NormalizeData(TIL5)

TIL6 <- NormalizeData(TIL6)

# Merge all samples

TILall_s <- merge(TIL1, y = c(TIL2,TIL3, TIL4, TIL5, TIL6),

add.cell.ids = c('RCC101',

'RCC113',

'RCC96',

'RCC100',

'RCC87',

'RCC104'),

project = "TILall_s", merge.data = TRUE)

# Unintegrated analysis

TILall_s <- NormalizeData(TILall_s)

TILall_s <- FindVariableFeatures(TILall_s, selection.method = "vst", nfeatures = 2000)

all.genes <- rownames(TILall_s)

TILall_s <- ScaleData(TILall_s, features = all.genes)

TILall_s <- RunPCA(TILall_s, features = VariableFeatures(object = TILall_s))

TILall_s <- FindNeighbors(TILall_s, dims = 1:10, reduction = "pca")

TILall_s <- FindClusters(TILall_s, resolution = 0.6, cluster.name = "unintegrated_clusters")

TILall_s <- RunUMAP(TILall_s, dims = 1:10, reduction = "pca", reduction.name = "umap.unintegrated")

DimPlot(TILall_s, reduction = "umap.unintegrated", group.by = c("orig.ident", "seurat_clusters"))

# ============================================

# Part 3. Integration (CCA)

# ============================================

TILall_s <- IntegrateLayers(object = TILall_s, method = CCAIntegration, orig.reduction = "pca", new.reduction = "integrated.cca",verbose = FALSE)

# Rejoin RNA layers

TILall_s[["RNA"]] <- JoinLayers(TILall_s[["RNA"]])

TILall_s <- FindNeighbors(TILall_s, reduction = "integrated.cca", dims = 1:10)

TILall_s <- FindClusters(TILall_s, resolution = 2.5)

TILall_s <- RunUMAP(TILall_s, dims = 1:10, reduction = "integrated.cca")

# ============================================

# Part 4. Marker expression and manual annotation

# ============================================

DotPlot(TILall_s, features = c('CD3E',

'CD4',

'CD8A',

'FOXP3',

'MS4A1',

'CD14',

'LYZ',

'FCGR3A',

'MS4A7',

'GNLY',

'NKG7',

'FCER1A',

'CST3',

'PPBP',

'IFNG',

'CD86',

'STAT1',

'IDO1',

'CD163',

'MRC1',

'IRF4',

'STAT6',

'PTPRC'),

scale.max = 100, scale.min = 0)+RotatedAxis()

# manual cluster annotation

new.cluster.ids <- c('CD8T',

'CD4T',

'CD8T',

'CD8T',

'NKT',

'CD8T',

'tumor',

'NKT',

'CD4T',

'M2',

'CD8T',

'tumor',

'tumor',

'CD8T',

'CD8T',

'tumor',

'tumor',

'tumor',

'NK',

'CD4T',

'DC',

'tumor',

'CD4T',

'Treg',

'tumor',

'tumor',

'M1',

'tumor',

'CD8T',

'B',

'DC',

'DC',

'DC',

'M0')

names(new.cluster.ids) <- levels(TILall_s)

TILall_s_rename <- RenameIdents(TILall_s, new.cluster.ids)

TILall_s_rename$celltype <- Idents(TILall_s_rename)

# <<<Figure 2A>>>

DimPlot(TILall_s_rename, reduction = "umap", label = TRUE, pt.size = 0.4) + NoLegend()

# ============================================

# Part 5. Build CIBERSORTx signature matrix

# ============================================

# ==== 1. Retrieve non-log-transformed expression matrix ====

expr_log <- LayerData(TILall_s_rename, assay = "RNA", layer = "data")

expr <- expm1(expr_log)

# ==== 2. Extract cell type information from metadata ====

celltype_vec <- TILall_s_rename$celltype

names(celltype_vec) <- colnames(TILall_s_rename)

# ==== 3. Subsample up to 50 cells per cell type ====

set.seed(123)

celltype_df <- data.frame(Cell = names(celltype_vec), CellType = as.character(celltype_vec))

subsampled_df <- celltype_df %>%

group_split(CellType) %>%

map_dfr(function(df) {

n_keep <- min(50, nrow(df))

df[sample(seq_len(nrow(df)), n_keep), ]

})

# ==== 4. Extract expression values for the selected cells ====

subsampled_cells <- subsampled_df$Cell

subsampled_celltypes <- subsampled_df$CellType

names(subsampled_celltypes) <- subsampled_cells

expr_sub <- expr[, subsampled_cells]

# ==== 5. Format for CIBERSORTx ====

expr_df <- as.data.frame(expr_sub)

expr_df$GeneSymbol <- rownames(expr_df)

expr_df <- expr_df[, c("GeneSymbol", subsampled_df$Cell)]

colnames(expr_df) <- c("GeneSymbol",

paste0(subsampled_df$CellType, "_",

seq_len(nrow(subsampled_df))))

write.table(

expr_df,

"CIBERSORTx_GEP_CelltypeHeader_nonlog_subsampled.txt",

sep = "\t", quote = FALSE,

row.names = FALSE,

fileEncoding = "UTF-8"

)

# ============================================

# Part 6. CIBERSORTx results visualization

# ============================================

# Proccess ccRCC bulk RNAseq data with above-created expression matrix in CIBERSORTx webtool ~~~~~~~~~~~~~~~~~~~

# save output file as "CIBERSORTx_Results.csv"

# add metadata information and save as "CIBERSORTx_Results_meta.csv"

# Load CIBERSORTx output

# Transpose with t() so that samples are rows

result_t <- read.csv("CIBERSORTx_Results_meta.csv", header = T, row.names = 1, fileEncoding = "UTF-8")

result_df <- as.data.frame(result_t)

result_df$Sample <- sub("C$", "", rownames(result_df))

# Long format

result_long <- pivot_longer(

result_df,

cols = c('tumor',

'M2',

'M1',

'M0',

'DC',

'B',

'NK',

'NKT',

'CD8T',

'CD4T',

'Treg'),

names_to = "CellType",

values_to = "Score")

result_percent <- result_long %>%

group_by(Sample) %>%

mutate(Percent = Score / sum(Score) * 100)

# Define sample order

result_percent$Sample <- factor(result_percent$Sample,

levels = c('KID001',

'KID002',

'KID018',

'KID012',

'KID016',

'KID013',

'KID006',

'KID015',

'KID011',

'KID004',

'KID014',

'KID005',

'KID007',

'KID009',

'KID010')) # ordered by diameter

# Specify CellType order and convert to factor

result_percent$CellType <- factor(result_percent$CellType,

levels = c('tumor',

'M2',

'M1',

'M0',

'DC',

'B',

'NK',

'NKT',

'CD8T',

'CD4T',

'Treg'))

# <<<Figure 2B>>>

ggplot(result_percent, aes(x = Sample, y = Percent, fill = CellType)) +

geom_bar(stat = "identity") +

theme_minimal() +

labs(x = "Sample",

y = "Cell Type Proportion (%)") +

theme(axis.text.x = element_text(angle = 90, hjust = 1, size = 16),

axis.title.x = element_text(size = 18),

axis.title.y = element_text(size = 18))+

scale_fill_manual(

values = c('B' = 'yellow',

'CD4T' = 'salmon',

'Treg' = 'pink',

'CD8T' = 'orange',

'DC' = 'red',

'M0' = 'skyblue',

'M1' = 'blue',

'M2' = 'lightgreen',

'NK' = 'green',

'NKT' = "darkgreen",

'tumor' = 'gray'))

# ============================================

# T cell abundance

# ============================================

# <<<Figure 2C left>>>

result_df$Sample <- factor(result_df$Sample,

levels = c('KID001',

'KID002',

'KID018',

'KID012',

'KID016',

'KID013',

'KID006',

'KID015',

'KID011',

'KID004',

'KID014',

'KID005',

'KID007',

'KID009',

'KID010')) #diameter order

result_df$Tcell_percent <- 100*rowSums(result_df[, c("CD4T",

"CD8T", "NKT", "Treg")], na.rm = TRUE)

ggplot(result_df, aes(x = Sample, y = Tcell_percent)) +

geom_bar(stat = "identity") +

labs(x = "Sample",

y = "T cell Percentage") +

theme_minimal() +

theme(axis.text.x = element_text(angle = 90, hjust = 1, vjust = 0.5, size = 16)) +

theme(

plot.title = element_text(size = 18, face = "bold", hjust = 0.5),

axis.title.x = element_text(size = 18),

axis.title.y = element_text(size = 18),

axis.text.x = element_text(size = 16),

axis.text.y = element_text(size = 16),

strip.text = element_text(size = 16, face = "bold"),

legend.text = element_text(size = 14),

legend.title = element_text(size = 14),

legend.position = "none"

)

# <<<Figure 2C right>>>

# Explicitly categorize tumor size (ordered)

result_df$size <- factor(

result_df$size,

levels = c("≤7cm", ">7cm"),

labels = c("≤7cm", ">7cm")

)

ggplot(result_df, aes(x = size, y = Tcell_percent, fill = size)) +

geom_boxplot(outlier.shape = NA, alpha = 0.6) +

geom_jitter(width = 0.2, size = 2, alpha = 0.7) +

stat_compare_means(

aes(x = size, y = Tcell_percent),

method = "t.test",

method.args = list(var.equal = TRUE, paired = FALSE),

label = "p.format",

label.y = 14,

size = 6

) +

labs(title = "T cell percentage by tumor diameter",

x = "Tumor diameter",

y = "T cell percentage") +

theme_minimal() +

theme(

plot.title = element_text(size = 18, face = "bold", hjust = 0.5),

axis.title.x = element_text(size = 18),

axis.title.y = element_text(size = 18),

axis.text.x = element_text(size = 24),

axis.text.y = element_text(size = 16),

strip.text = element_text(size = 16, face = "bold"),

legend.text = element_text(size = 14),

legend.title = element_text(size = 14),

legend.position = "none"

)

# ============================================

# Macrophage subset analysis

# ============================================

# <<<Figure 2D>>>

df <- read.csv("CIBERSORTxResults.csv", row.names = 1)

# Explicitly categorize tumor size (ordered)

df$size <- factor(df$size, levels = c("≤7cm", ">7cm"), labels = c("≤7cm", ">7cm"))

m_df <- df[, c("M0", "M1", "M2")]

m_total <- rowSums(m_df, na.rm = TRUE)

m_percent <- m_df / m_total * 100

m_percent$Sample <- rownames(m_percent)

m_long <- pivot_longer(m_percent, cols = -Sample,

names_to = "Subtype", values_to = "RelativePercent")

# Merge metadata information

meta_data <- df[, "size", drop = FALSE] %>%

mutate(Sample = rownames(.))

m_long <- left_join(m_long, meta_data, by = "Sample")

# Compute 1.05× the maximum value per subtype for p-value label positions

label_pos_df <- m_long %>%

group_by(Subtype) %>%

summarise(label_y = max(RelativePercent, na.rm = TRUE)*1.05 )

# Specify comparisons here (two-group comparison)

my_comparisons <- list(c("≤7cm", ">7cm")) # adjust according to size categories

# Plot

ggplot(m_long, aes(x = size, y = RelativePercent, fill = size)) +

geom_boxplot(outlier.shape = NA, alpha = 0.7) +

geom_jitter(position = position_jitter(width = 0.2), size = 1.5, alpha = 0.6) +

stat_compare_means(

method = "t.test",

method.args = list(var.equal = TRUE, paired = FALSE),

label = "p.format",

size = 6,

label.y = label_pos_df$label_y

) +

facet_wrap(~Subtype, scales = "fixed", ncol = 3) +

scale_y_continuous(limits = c(-5, 115), breaks = seq(0, 100, 20), expand = c(0, 0))+

labs(x = "Diameter", y = "Percentage") +

theme_minimal() +

theme(

axis.title.x = element_text(size = 20),

axis.title.y = element_text(size = 20),

axis.text.x = element_text(size = 14),

axis.text.y = element_text(size = 14),

strip.text = element_text(size = 16, face = "bold"),

legend.text = element_text(size = 14),

legend.title = element_text(size = 14),

legend.position = "none"

)

# =======<<<<sessionInfo()>>>>==================================

R version 4.5.1 (2025-06-13 ucrt)

Platform: x86_64-w64-mingw32/x64

Running under: Windows 11 x64 (build 26200)

Matrix products: default

LAPACK version 3.12.1

locale:

[1] LC_COLLATE=Japanese_Japan.utf8 LC_CTYPE=Japanese_Japan.utf8 LC_MONETARY=Japanese_Japan.utf8

[4] LC_NUMERIC=C LC_TIME=Japanese_Japan.utf8

time zone: Asia/Tokyo

tzcode source: internal

attached base packages:

[1] stats graphics grDevices utils datasets methods base

other attached packages:

[1] ggpubr_0.6.2 lubridate_1.9.4 forcats_1.0.1 stringr_1.6.0 purrr_1.2.0

[6] readr_2.1.5 tidyr_1.3.1 tibble_3.3.0 ggplot2_4.0.0 tidyverse_2.0.0

[11] dplyr_1.1.4 Seurat_5.3.1 SeuratObject_5.2.0 sp_2.2-0

loaded via a namespace (and not attached):

[1] deldir_2.0-4 pbapply_1.7-4 gridExtra_2.3 rlang_1.1.6

[5] magrittr_2.0.4 RcppAnnoy_0.0.22 otel_0.2.0 matrixStats_1.5.0

[9] ggridges_0.5.7 compiler_4.5.1 spatstat.geom_3.6-0 png_0.1-8

[13] vctrs_0.6.5 reshape2_1.4.5 pkgconfig_2.0.3 fastmap_1.2.0

[17] backports_1.5.0 promises_1.5.0 tzdb_0.5.0 jsonlite_2.0.0

[21] goftest_1.2-3 later_1.4.4 spatstat.utils_3.2-0 broom_1.0.11

[25] irlba_2.3.5.1 parallel_4.5.1 cluster_2.1.8.1 R6_2.6.1

[29] ica_1.0-3 stringi_1.8.7 RColorBrewer_1.1-3 spatstat.data_3.1-9

[33] reticulate_1.44.0 car_3.1-3 parallelly_1.45.1 spatstat.univar_3.1-4

[37] lmtest_0.9-40 scattermore_1.2 Rcpp_1.1.0 tensor_1.5.1

[41] future.apply_1.20.1 zoo_1.8-14 sctransform_0.4.2 timechange_0.3.0

[45] httpuv_1.6.16 Matrix_1.7-4 splines_4.5.1 igraph_2.2.1

[49] tidyselect_1.2.1 rstudioapi_0.17.1 dichromat_2.0-0.1 abind_1.4-8

[53] spatstat.random_3.4-2 codetools_0.2-20 miniUI_0.1.2 spatstat.explore_3.5-3

[57] listenv_0.10.0 lattice_0.22-7 plyr_1.8.9 withr_3.0.2

[61] shiny_1.12.1 S7_0.2.0 ROCR_1.0-11 Rtsne_0.17

[65] future_1.68.0 fastDummies_1.7.5 survival_3.8-3 polyclip_1.10-7

[69] fitdistrplus_1.2-4 pillar_1.11.1 carData_3.0-5 KernSmooth_2.23-26

[73] plotly_4.11.0 generics_0.1.4 RcppHNSW_0.6.0 hms_1.1.4

[77] scales_1.4.0 globals_0.18.0 xtable_1.8-4 glue_1.8.0

[81] lazyeval_0.2.2 tools_4.5.1 data.table_1.17.8 RSpectra_0.16-2

[85] ggsignif_0.6.4 RANN_2.6.2 dotCall64_1.2 cowplot_1.2.0

[89] grid_4.5.1 nlme_3.1-168 patchwork_1.3.2 Formula_1.2-5

[93] cli_3.6.5 spatstat.sparse_3.1-0 spam_2.11-1 viridisLite_0.4.2

[97] uwot_0.2.4 gtable_0.3.6 rstatix_0.7.3 digest_0.6.38

[101] progressr_0.18.0 ggrepel_0.9.6 htmlwidgets_1.6.4 farver_2.1.2

[105] htmltools_0.5.8.1 lifecycle_1.0.4 httr_1.4.7 mime_0.13

[109] MASS_7.3-65
