# Supplementary material for: Tumor-reactive TCRs within exhausted TILs reveal cancer type-specific immune landscapes in renal cell carcinoma
Source: Front Immunol. 2026 Jan 29;17:1729388. doi: 10.3389/fimmu.2026.1729388 (PMC12894369; doi:10.3389/fimmu.2026.1729388)
Supplement: Supplementary file 3 [file DataSheet3.docx]

## Supplementary Text 3

## ROC-based tumor diameter cutoff for separating cell type proportion

# =========================================================

# Input: stat.csv

# Output: optimal cutoff (Youden), AUC, and a plot

# =========================================================

setwd("path/to/the folder with stat.csv")

suppressPackageStartupMessages({

library(tidyverse)

library(pROC)

})

### CD8Tex

# 1) Load

df <- read.csv("stat.csv", stringsAsFactors = FALSE) %>%

transmute(

diameter = as.numeric(diameter),

CD8Tex = as.numeric(CD8Tex)

) %>%

filter(!is.na(diameter), !is.na(CD8Tex))

# 2) Define binary outcome (Tex-high vs Tex-low)

tex_threshold <- median(df$CD8Tex, na.rm = TRUE)

df <- df %>%

mutate(TexHigh = if_else(CD8Tex >= tex_threshold, 1L, 0L))

# 3) ROC: predictor = diameter

roc_obj <- roc(

response = df$TexHigh,

predictor = df$diameter,

direction = "<",

ci = TRUE

)

# 4) Best cutoff by Youden index

best <- coords(

roc_obj,

x = "best",

best.method = "youden",

ret = c("threshold", "sensitivity", "specificity", "youden"),

transpose = FALSE

)

cat("Tex-high definition: CD8Tex >= median =", tex_threshold, "\n\n")

print(best)

cat("\nAUC =", as.numeric(auc(roc_obj)), "\n")

cat("AUC 95% CI =", paste(round(ci.auc(roc_obj), 3), collapse = " - "), "\n")

# 5) Plot

plot(roc_obj, main = "ROC: tumor diameter predicting CD8Tex-high")

abline(a = 0, b = 1, lty = 2)

# 6) Bootstrap stability of cutoff (fully robust)

set.seed(123)

B <- 2000

boot_cut <- replicate(B, {

idx <- sample(seq_len(nrow(df)), replace = TRUE)

if (length(unique(df$TexHigh[idx])) < 2) return(NA_real_)

r <- tryCatch(

roc(df$TexHigh[idx], df$diameter[idx], direction = "<", quiet = TRUE),

error = function(e) NULL

)

if (is.null(r)) return(NA_real_)

th <- tryCatch(

coords(r, x = "best", best.method = "youden",

ret = c("threshold", "youden"),

transpose = FALSE),

error = function(e) NULL

)

if (is.null(th)) return(NA_real_)

m <- as.matrix(th)

if (!("threshold" %in% colnames(m)) && ("threshold" %in% rownames(m))) {

m <- t(m)

}

if (!all(c("threshold", "youden") %in% colnames(m))) return(NA_real_)

m <- m[order(-as.numeric(m[, "youden"])), , drop = FALSE]

as.numeric(m[1, "threshold"])

})

boot_cut_ok <- boot_cut[!is.na(boot_cut)]

cat("\nBootstrap valid iterations:", length(boot_cut_ok), "/", B, "\n")

print(summary(boot_cut_ok))

cat("Bootstrap 95% interval:",

paste(round(quantile(boot_cut_ok, c(0.025, 0.975)), 3), collapse = " - "),

"\n")

### CD8Tpex

# 1) Load

df <- read.csv("stat.csv", stringsAsFactors = FALSE) %>%

transmute(

diameter = as.numeric(diameter),

CD8Tpex = as.numeric(CD8Tpex)

) %>%

filter(!is.na(diameter), !is.na(CD8Tpex))

# 2) Define binary outcome (Tpex-high vs Tpex-low)

Tpex_threshold <- median(df$CD8Tpex, na.rm = TRUE)

df <- df %>%

mutate(TpexHigh = if_else(CD8Tpex >= Tpex_threshold, 1L, 0L))

# 3) ROC: predictor = diameter

roc_obj <- roc(

response = df$TpexHigh,

predictor = df$diameter,

direction = ">",

ci = TRUE

)

# 4) Best cutoff by Youden index

best <- coords(

roc_obj,

x = "best",

best.method = "youden",

ret = c("threshold", "sensitivity", "specificity", "youden"),

transpose = FALSE

)

cat("Tex-high definition: CD8Tpex >= median =", tex_threshold, "\n\n")

print(best)

cat("\nAUC =", as.numeric(auc(roc_obj)), "\n")

cat("AUC 95% CI =", paste(round(ci.auc(roc_obj), 3), collapse = " - "), "\n")

# 5) Plot

plot(roc_obj, main = "ROC: tumor diameter predicting CD8Tpex-low")

abline(a = 0, b = 1, lty = 2)

# 6) Bootstrap stability of cutoff (fully robust)

set.seed(123)

B <- 2000

boot_cut <- replicate(B, {

idx <- sample(seq_len(nrow(df)), replace = TRUE)

if (length(unique(df$TpexHigh[idx])) < 2) return(NA_real_)

r <- tryCatch(

roc(df$TpexHigh[idx], df$diameter[idx], direction = ">", quiet = TRUE),

error = function(e) NULL

)

if (is.null(r)) return(NA_real_)

th <- tryCatch(

coords(r, x = "best", best.method = "youden",

ret = c("threshold", "youden"),

transpose = FALSE),

error = function(e) NULL

)

if (is.null(th)) return(NA_real_)

m <- as.matrix(th)

if (!("threshold" %in% colnames(m)) && ("threshold" %in% rownames(m))) {

m <- t(m)

}

if (!all(c("threshold", "youden") %in% colnames(m))) return(NA_real_)

m <- m[order(-as.numeric(m[, "youden"])), , drop = FALSE]

as.numeric(m[1, "threshold"])

})

boot_cut_ok <- boot_cut[!is.na(boot_cut)]

cat("\nBootstrap valid iterations:", length(boot_cut_ok), "/", B, "\n")

print(summary(boot_cut_ok))

cat("Bootstrap 95% interval:",

paste(round(quantile(boot_cut_ok, c(0.025, 0.975)), 3), collapse = " - "),

"\n")

### CD8Teff

# 1) Load

df <- read.csv("stat.csv", stringsAsFactors = FALSE) %>%

transmute(

diameter = as.numeric(diameter),

CD8Teff = as.numeric(CD8Teff)

) %>%

filter(!is.na(diameter), !is.na(CD8Teff))

# 2) Define binary outcome (Teff-high vs Teff-low)

Teff_threshold <- median(df$CD8Teff, na.rm = TRUE)

df <- df %>%

mutate(TeffHigh = if_else(CD8Teff >= Teff_threshold, 1L, 0L))

# 3) ROC: predictor = diameter

roc_obj <- roc(

response = df$TeffHigh,

predictor = df$diameter,

direction = ">",

ci = TRUE

)

# 4) Best cutoff by Youden index

best <- coords(

roc_obj,

x = "best",

best.method = "youden",

ret = c("threshold", "sensitivity", "specificity", "youden"),

transpose = FALSE

)

cat("Tex-high definition: CD8Teff >= median =", tex_threshold, "\n\n")

print(best)

cat("\nAUC =", as.numeric(auc(roc_obj)), "\n")

cat("AUC 95% CI =", paste(round(ci.auc(roc_obj), 3), collapse = " - "), "\n")

# 5) Plot

plot(roc_obj, main = "ROC: tumor diameter predicting CD8Teff-low")

abline(a = 0, b = 1, lty = 2)

# 6) Bootstrap stability of cutoff (fully robust)

set.seed(123)

B <- 2000

boot_cut <- replicate(B, {

idx <- sample(seq_len(nrow(df)), replace = TRUE)

if (length(unique(df$TeffHigh[idx])) < 2) return(NA_real_)

r <- tryCatch(

roc(df$TeffHigh[idx], df$diameter[idx], direction = ">", quiet = TRUE),

error = function(e) NULL

)

if (is.null(r)) return(NA_real_)

th <- tryCatch(

coords(r, x = "best", best.method = "youden",

ret = c("threshold", "youden"),

transpose = FALSE),

error = function(e) NULL

)

if (is.null(th)) return(NA_real_)

m <- as.matrix(th)

if (!("threshold" %in% colnames(m)) && ("threshold" %in% rownames(m))) {

m <- t(m)

}

if (!all(c("threshold", "youden") %in% colnames(m))) return(NA_real_)

m <- m[order(-as.numeric(m[, "youden"])), , drop = FALSE]

as.numeric(m[1, "threshold"])

})

boot_cut_ok <- boot_cut[!is.na(boot_cut)]

cat("\nBootstrap valid iterations:", length(boot_cut_ok), "/", B, "\n")

print(summary(boot_cut_ok))

cat("Bootstrap 95% interval:",

paste(round(quantile(boot_cut_ok, c(0.025, 0.975)), 3), collapse = " - "),

"\n")

### CD4Treg

# 1) Load

df <- read.csv("stat.csv", stringsAsFactors = FALSE) %>%

transmute(

diameter = as.numeric(diameter),

CD4Treg = as.numeric(CD4Treg)

) %>%

filter(!is.na(diameter), !is.na(CD4Treg))

# 2) Define binary outcome (Treg-high vs Treg-low)

Treg_threshold <- median(df$CD4Treg, na.rm = TRUE)

df <- df %>%

mutate(TregHigh = if_else(CD4Treg >= Treg_threshold, 1L, 0L))

# 3) ROC: predictor = diameter

roc_obj <- roc(

response = df$TregHigh,

predictor = df$diameter,

direction = "<",

ci = TRUE

)

# 4) Best cutoff by Youden index

best <- coords(

roc_obj,

x = "best",

best.method = "youden",

ret = c("threshold", "sensitivity", "specificity", "youden"),

transpose = FALSE

)

cat("Treg-high definition: CD4Treg >= median =", tex_threshold, "\n\n")

print(best)

cat("\nAUC =", as.numeric(auc(roc_obj)), "\n")

cat("AUC 95% CI =", paste(round(ci.auc(roc_obj), 3), collapse = " - "), "\n")

# 5) Plot

plot(roc_obj, main = "ROC: tumor diameter predicting CD4Treg-high")

abline(a = 0, b = 1, lty = 2)

# 6) Bootstrap stability of cutoff (fully robust)

set.seed(123)

B <- 2000

boot_cut <- replicate(B, {

idx <- sample(seq_len(nrow(df)), replace = TRUE)

if (length(unique(df$TregHigh[idx])) < 2) return(NA_real_)

r <- tryCatch(

roc(df$TregHigh[idx], df$diameter[idx], direction = "<", quiet = TRUE),

error = function(e) NULL

)

if (is.null(r)) return(NA_real_)

th <- tryCatch(

coords(r, x = "best", best.method = "youden",

ret = c("threshold", "youden"),

transpose = FALSE),

error = function(e) NULL

)

if (is.null(th)) return(NA_real_)

m <- as.matrix(th)

if (!("threshold" %in% colnames(m)) && ("threshold" %in% rownames(m))) {

m <- t(m)

}

if (!all(c("threshold", "youden") %in% colnames(m))) return(NA_real_)

m <- m[order(-as.numeric(m[, "youden"])), , drop = FALSE]

as.numeric(m[1, "threshold"])

})

boot_cut_ok <- boot_cut[!is.na(boot_cut)]

cat("\nBootstrap valid iterations:", length(boot_cut_ok), "/", B, "\n")

print(summary(boot_cut_ok))

cat("Bootstrap 95% interval:",

paste(round(quantile(boot_cut_ok, c(0.025, 0.975)), 3), collapse = " - "),

"\n")

####<<sessionInfo()>>########

R version 4.5.1 (2025-06-13 ucrt)

Platform: x86_64-w64-mingw32/x64

Running under: Windows 11 x64 (build 26200)

Matrix products: default

LAPACK version 3.12.1

locale:

[1] LC_COLLATE=Japanese_Japan.utf8 LC_CTYPE=Japanese_Japan.utf8 LC_MONETARY=Japanese_Japan.utf8

[4] LC_NUMERIC=C LC_TIME=Japanese_Japan.utf8

time zone: Asia/Tokyo

tzcode source: internal

attached base packages:

[1] stats graphics grDevices utils datasets methods base

other attached packages:

[1] pROC_1.19.0.1 lubridate_1.9.4 forcats_1.0.1 stringr_1.6.0 dplyr_1.1.4 purrr_1.2.0

[7] readr_2.1.5 tidyr_1.3.1 tibble_3.3.0 ggplot2_4.0.0 tidyverse_2.0.0

loaded via a namespace (and not attached):

[1] vctrs_0.6.5 cli_3.6.5 rlang_1.1.6 stringi_1.8.7 generics_0.1.4

[6] S7_0.2.0 glue_1.8.0 hms_1.1.4 scales_1.4.0 grid_4.5.1

[11] tzdb_0.5.0 lifecycle_1.0.4 compiler_4.5.1 RColorBrewer_1.1-3 Rcpp_1.1.0

[16] timechange_0.3.0 pkgconfig_2.0.3 rstudioapi_0.17.1 farver_2.1.2 R6_2.6.1

[21] dichromat_2.0-0.1 tidyselect_1.2.1 pillar_1.11.1 magrittr_2.0.4 tools_4.5.1

[26] withr_3.0.2 gtable_0.3.6
