# Supplementary material for: Tumor-reactive TCRs within exhausted TILs reveal cancer type-specific immune landscapes in renal cell carcinoma
Source: Front Immunol. 2026 Jan 29;17:1729388. doi: 10.3389/fimmu.2026.1729388 (PMC12894369; doi:10.3389/fimmu.2026.1729388)
Supplement: Supplementary file 4 [file Presentation1.pdf]

Tumor-reactive TCRs within exhausted TILs reveal cancer type-specific immune landscapes in renal cell carcinoma

Mitsuru Komahashi[1,2,3]*, Shun Horaguchi[1,2,3]*, Kayoko Tsuji[1,2]*, Daisuke Hoshino[4], Takeshi Kishida[5], Kimitsugu Usui[5], Noboru Nakaigawa[5], Shinya Sato[6,7], Hiroshi Hamana[8], Hiroyuki Kishi[8], Feifei Wei[1,2], Yasunobu Mano[1,2], Taku Kouro[1,2#], Shuichiro Uehara[3#] and Tetsuro Sasada[1,2#]

Supplementary Figures

Figure S1

A

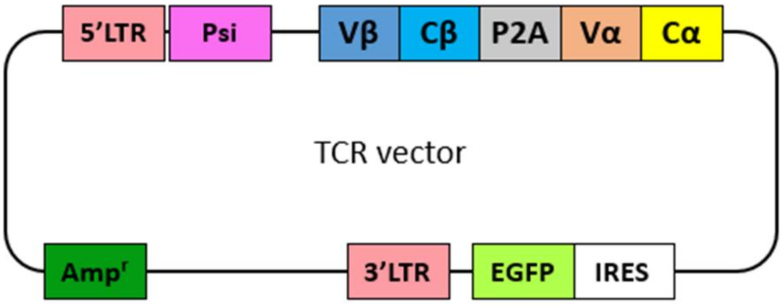
5'LTR
Psi
Vβ
Cβ
P2A
Vα
Cα
TCR vector
Amp^r
3'LTR
EGFP
IRES

B

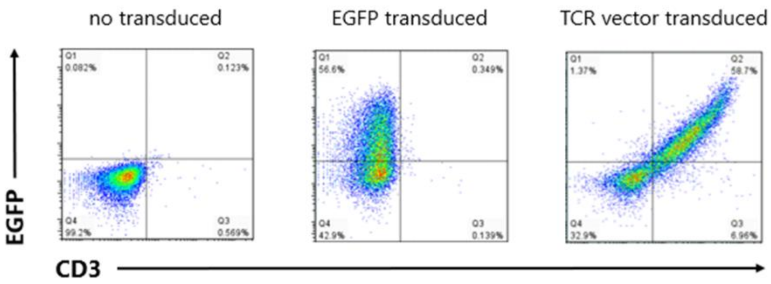
no transduced
EGFP transduced
TCR vector transduced
EGFP
CD3

Figure S1. Construction of TCR expression vectors and confirmation of their expression on T cells

(A) Schematic illustration of TCR expression vector containing artificially synthesized Vα and Vβ constructs derived from the top 20 most abundant TCR clonotypes. (B) Confirmation of proper TCR complex assembly, assessed by surface CD3ε expression. Representative flow cytometry plots showing CD3ε and EGFP expression in Jurkat Δαβ CD8a cells transduced with EGFP-containing expression vectors with or without TCR constructs.

Figure S2

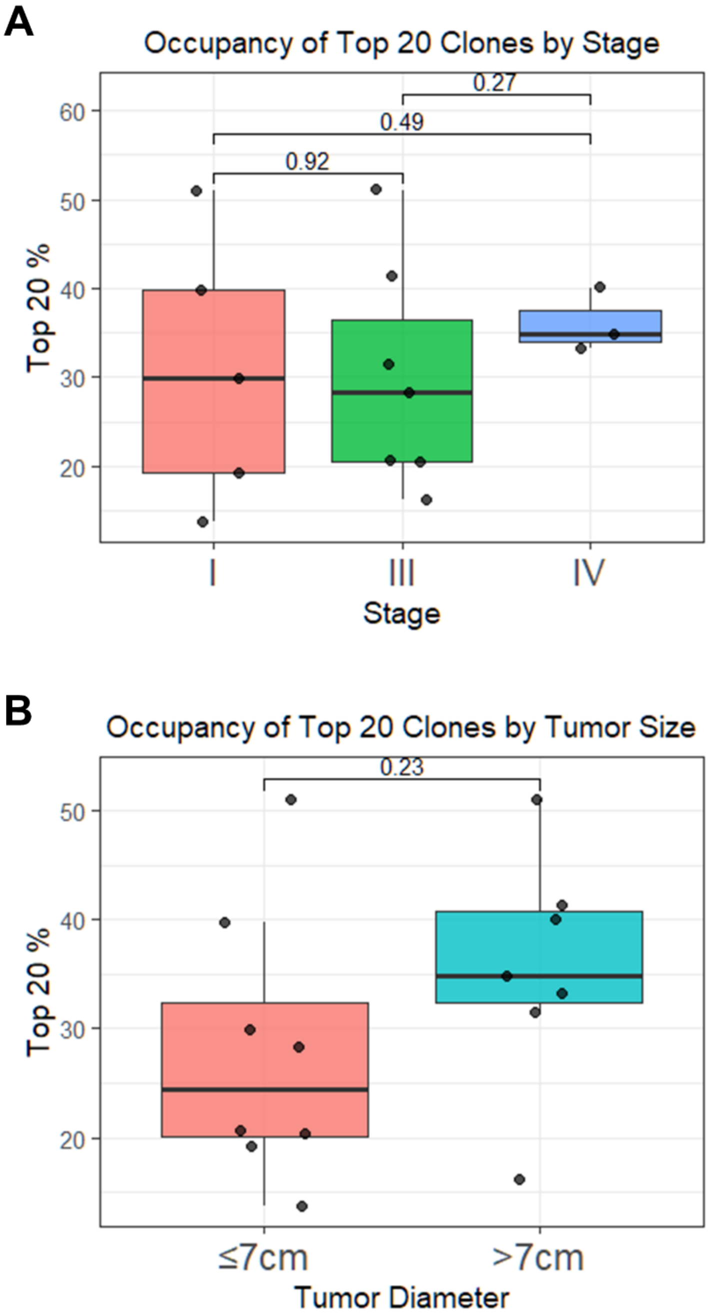
A
Occupancy of Top 20 Clones by Stage
0.27
0.49
0.92
60
50
40
30
20
Top 20 %
I
III
IV
Stage
B
Occupancy of Top 20 Clones by Tumor Size
0.23
50
40
30
20
Top 20 %
≤7cm
>7cm
Tumor Diameter

Figure S2. Clonality of T cells with the top 20 most abundant clonotypes.

The occupancy of the top 20 T cell clonotypes in each sample was calculated and plotted. (A) Comparison of top 20 clonotype occupancy by tumor stage. (B) Comparison of top 20 clonotype occupancy by tumor size. *P* values were determined using the Student's t test.

Figure S3

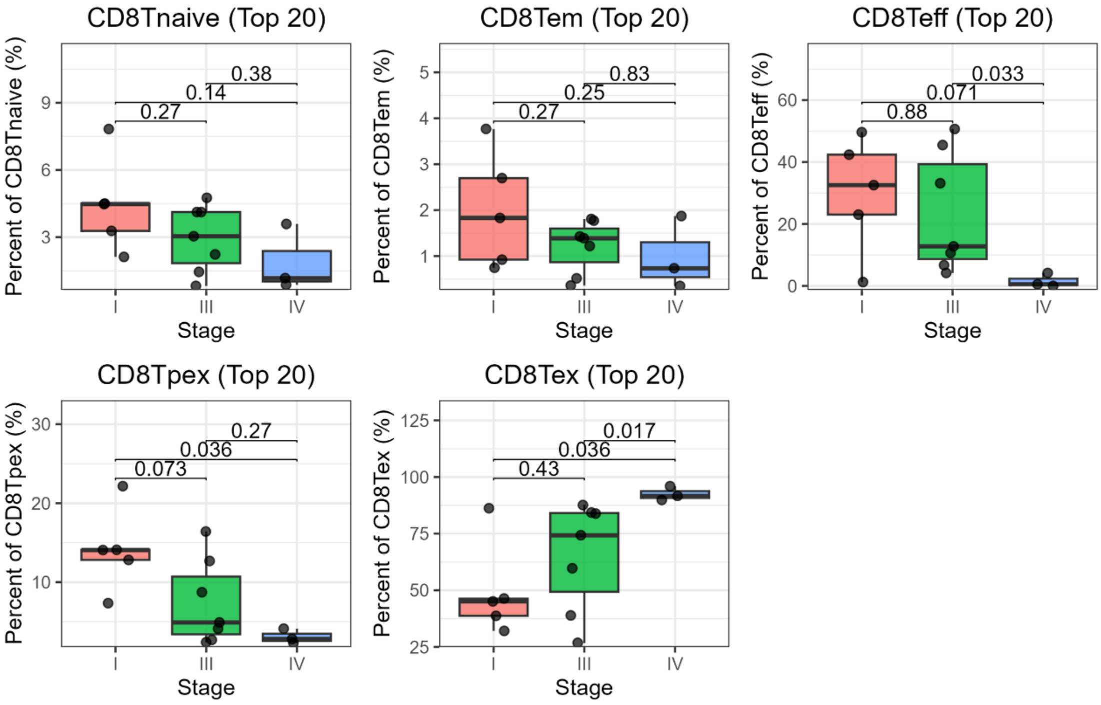

CD8Tnaive (Top 20)
Percent of CD8Tnaive (%)
0.38
0.14
0.27
Stage
CD8Tem (Top 20)
Percent of CD8Tem (%)
0.83
0.25
0.27
Stage
CD8Teff (Top 20)
Percent of CD8Teff (%)
0.033
0.071
0.88
Stage
CD8Tpex (Top 20)
Percent of CD8Tpex (%)
0.27
0.036
0.073
Stage
CD8Tex (Top 20)
Percent of CD8Tex (%)
0.017
0.036
0.43
Stage
I
III
IV

Figure S3. Cell type distribution of the top 20 most abundant clonotypes by stage. The composition of T cell subsets was quantified for T cells with the top 20 most abundant clonotypes and compared across tumor stages. In each box plot, each dot represents an individual sample. *P* values were determined using the Student's t test.

Figure S4

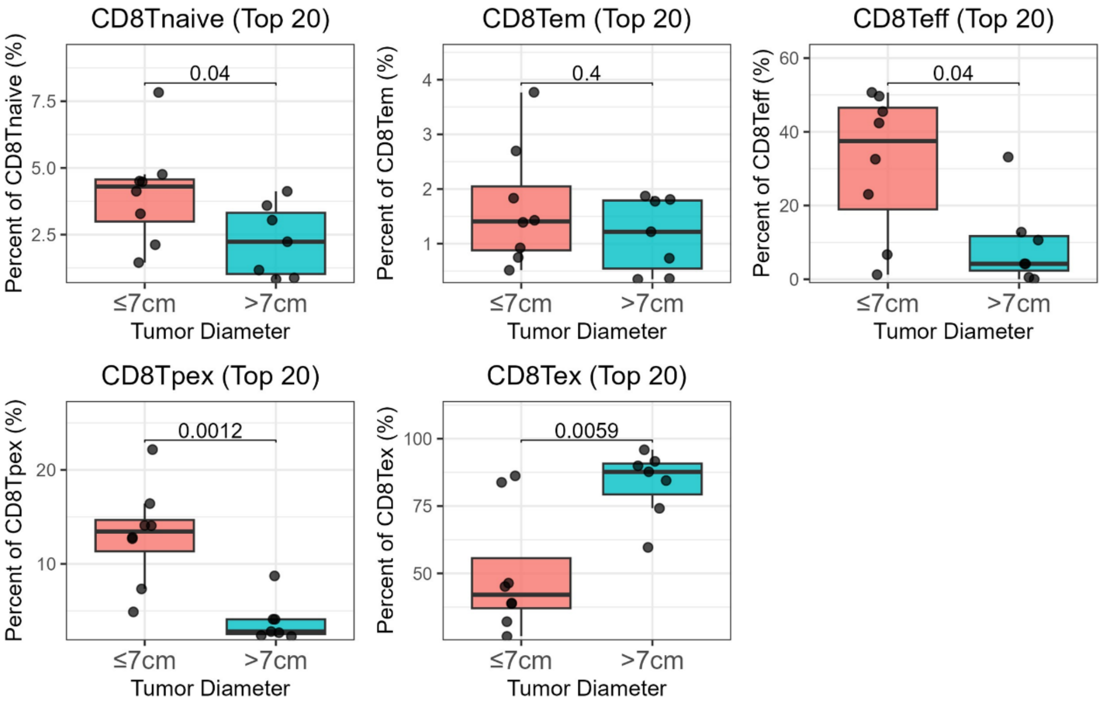

CD8Tnaive (Top 20)
Percent of CD8Tnaive (%)
0.04
≤7cm
>7cm
Tumor Diameter
CD8Tem (Top 20)
Percent of CD8Tem (%)
0.4
CD8Teff (Top 20)
Percent of CD8Teff (%)
0.04
CD8Tpex (Top 20)
Percent of CD8Tpex (%)
0.0012
CD8Tex (Top 20)
Percent of CD8Tex (%)
0.0059

Figure S4. Cell type distribution of the top 20 most abundant clonotypes by tumor size.

The composition of T cell subsets was quantified for T cells with the top 20 most abundant clonotypes and compared between small (≤ 7cm, n = 8) and large (>7cm, n = 7) tumors. In each box plot, each dot represents an individual sample. *P* values were determined using the Student's t test.

Figure S5

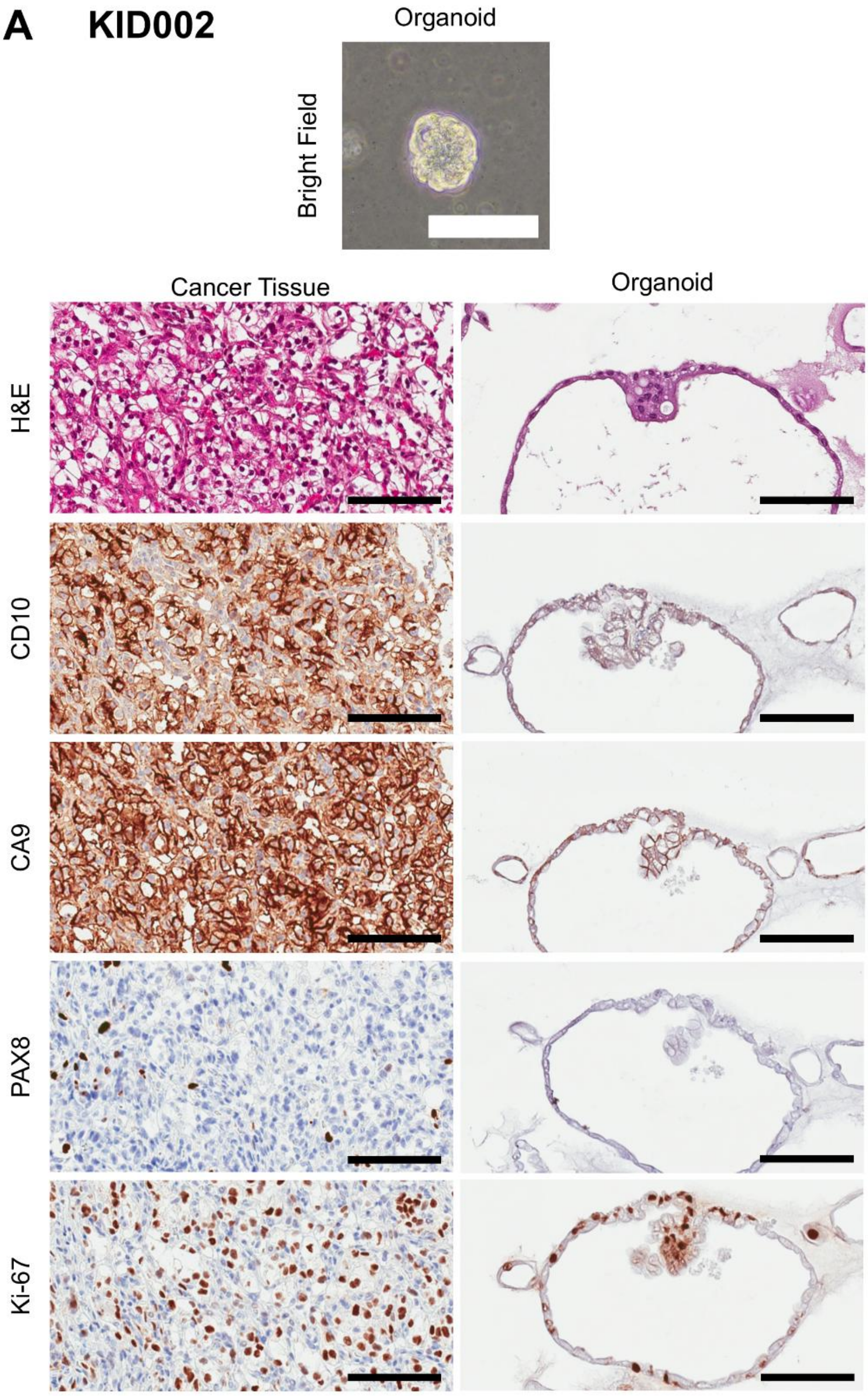
A
KID002
Organoid
Bright Field
Cancer Tissue
Organoid
H&E
CD10
CA9
PAX8
Ki-67

Figure S5

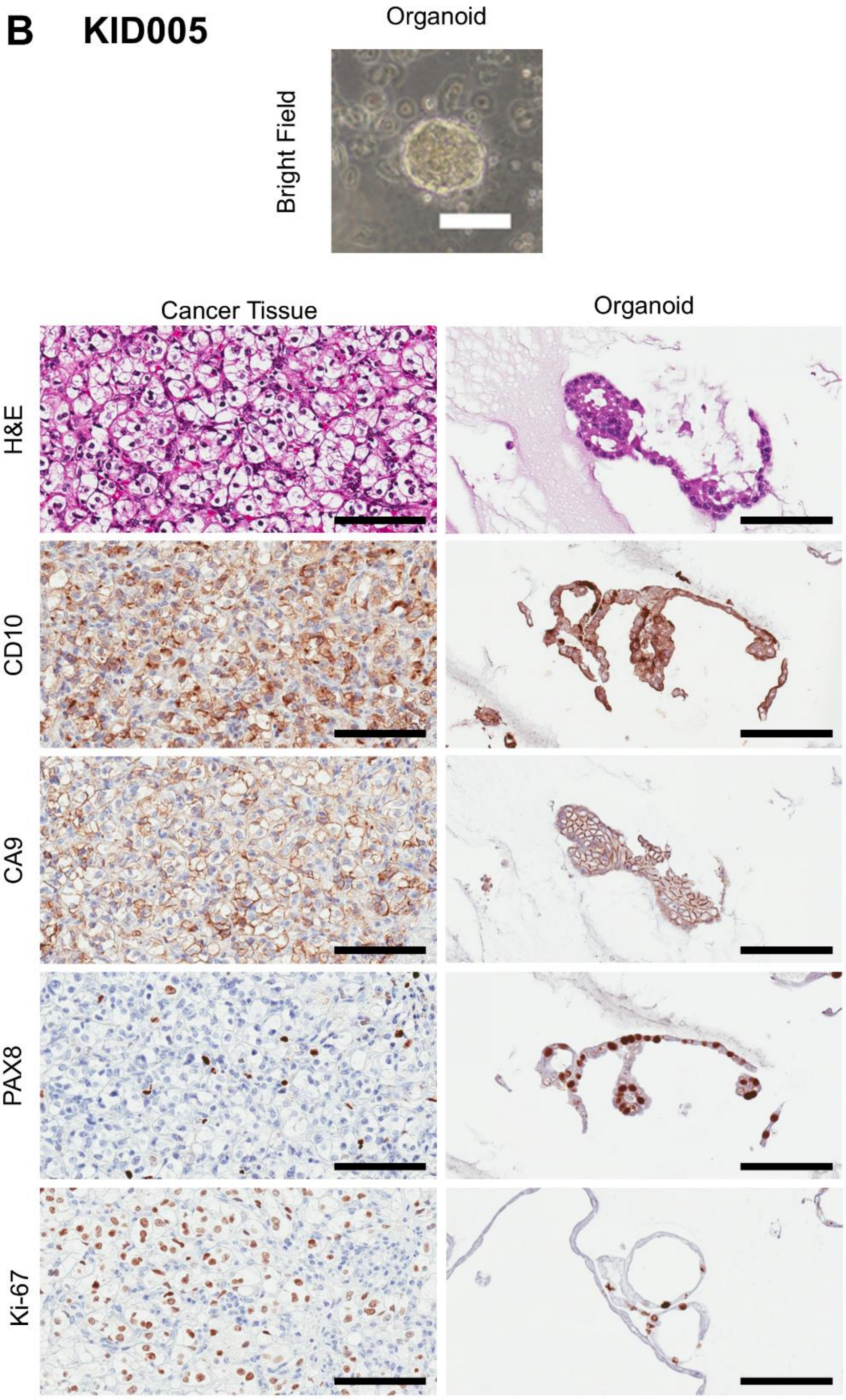
B
KID005
Organoid
Bright Field
Cancer Tissue
Organoid
H&E
CD10
CA9
PAX8
Ki-67

Figure S5

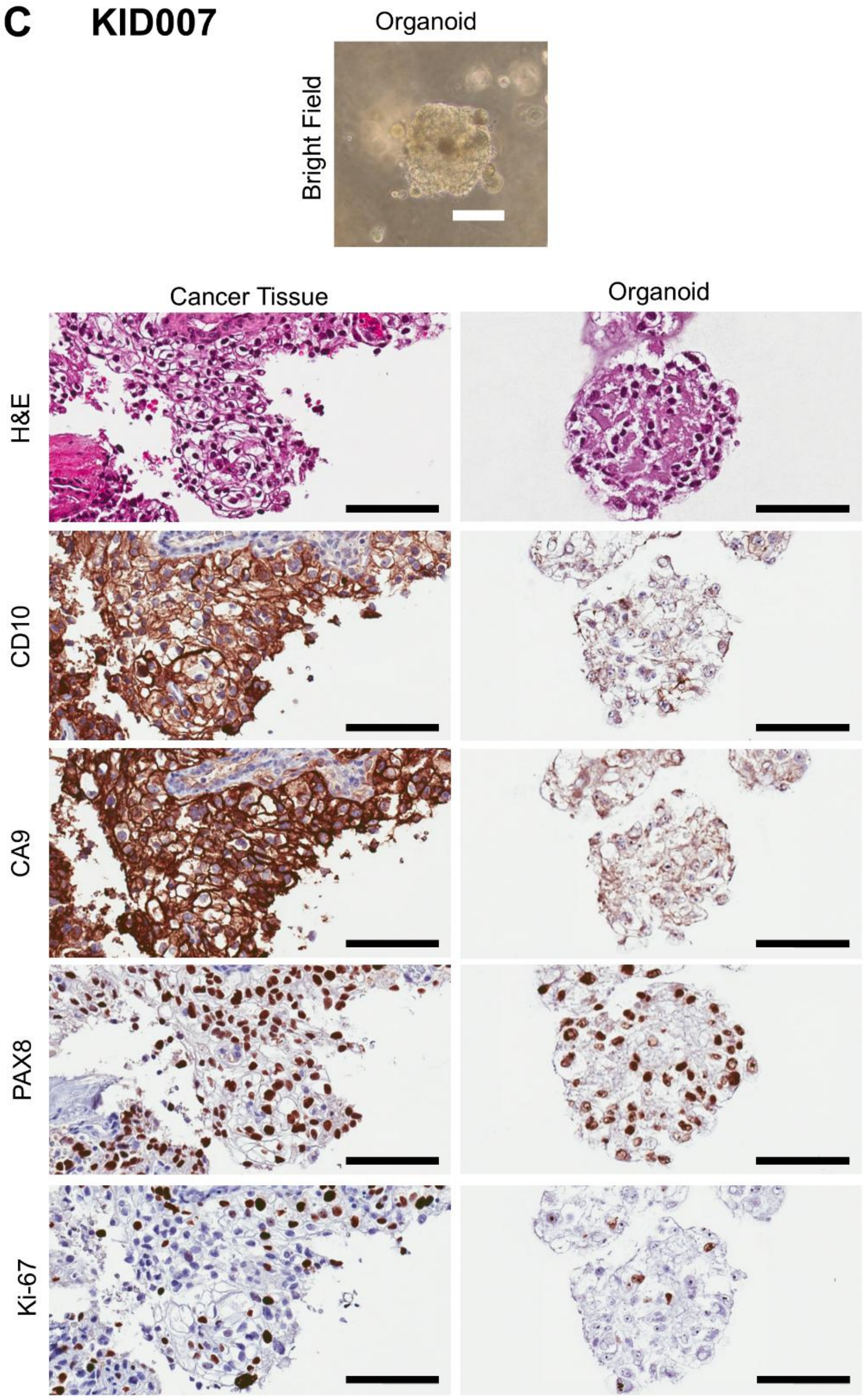
C
KID007
Organoid
Bright Field
Cancer Tissue
Organoid
H&E
CD10
CA9
PAX8
Ki-67

Figure S5

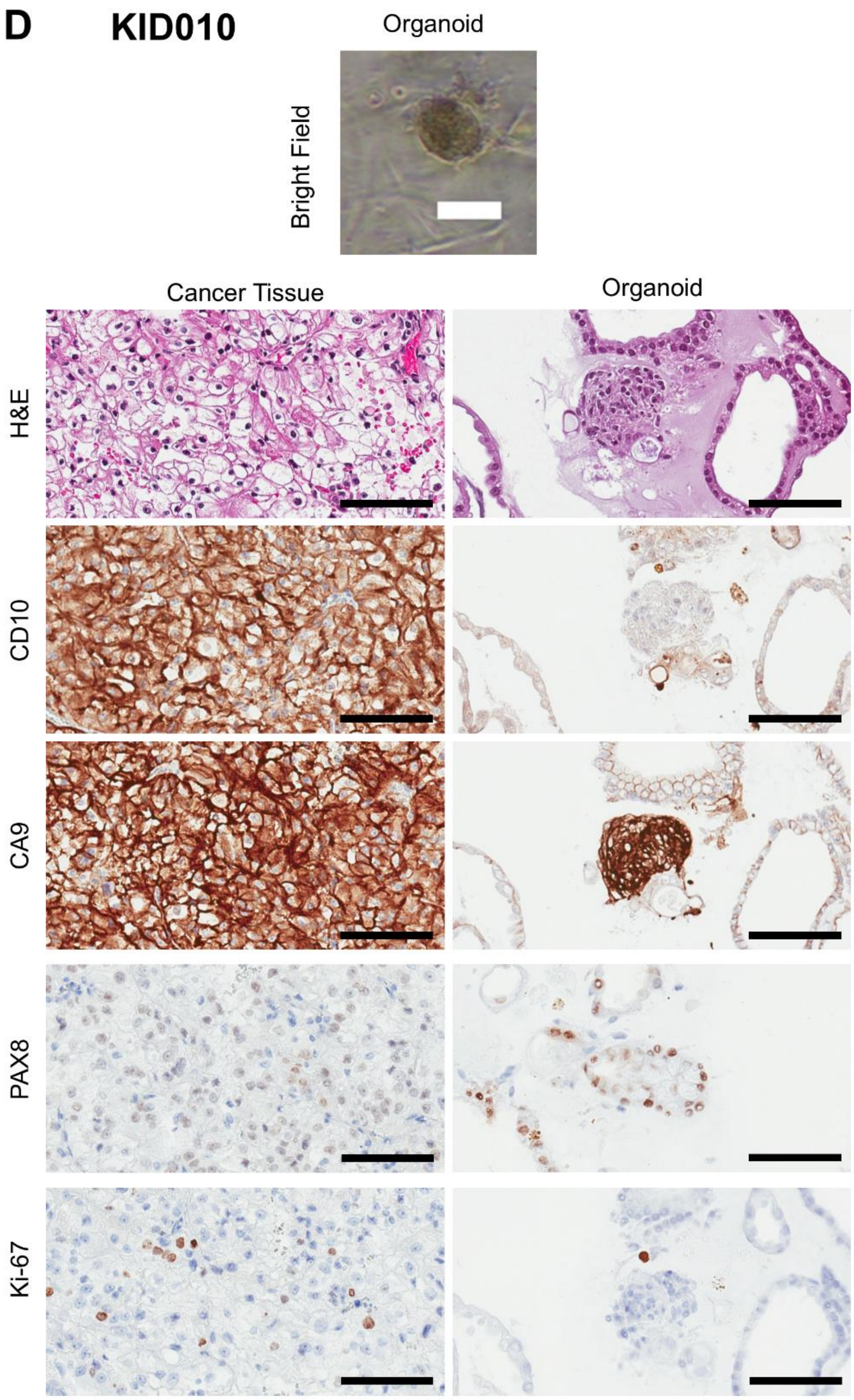
D
KID010
Organoid
Bright Field
Cancer Tissue
Organoid
H&E
CD10
CA9
PAX8
Ki-67

Figure S5. Establishment and phenotyping of ccRCC-derived organoids

Representative phase-contrast microscopic images of ccRCC-derived organoids: KID002 (A), KID005 (B), KID007 (C), and KID010 (D). Representative hematoxylin–eosin staining and immunohistochemical staining for ccRCC-related antigens (CD10, CA9, and PAX8) and proliferation marker (Ki-67) in original ccRCC tissues and ccRCC-derived organoids: KID002 (A) , KID005 (B), KID007 (C), and KID010 (D). Scale bars: 100 μm.

Figure S6

A KID001

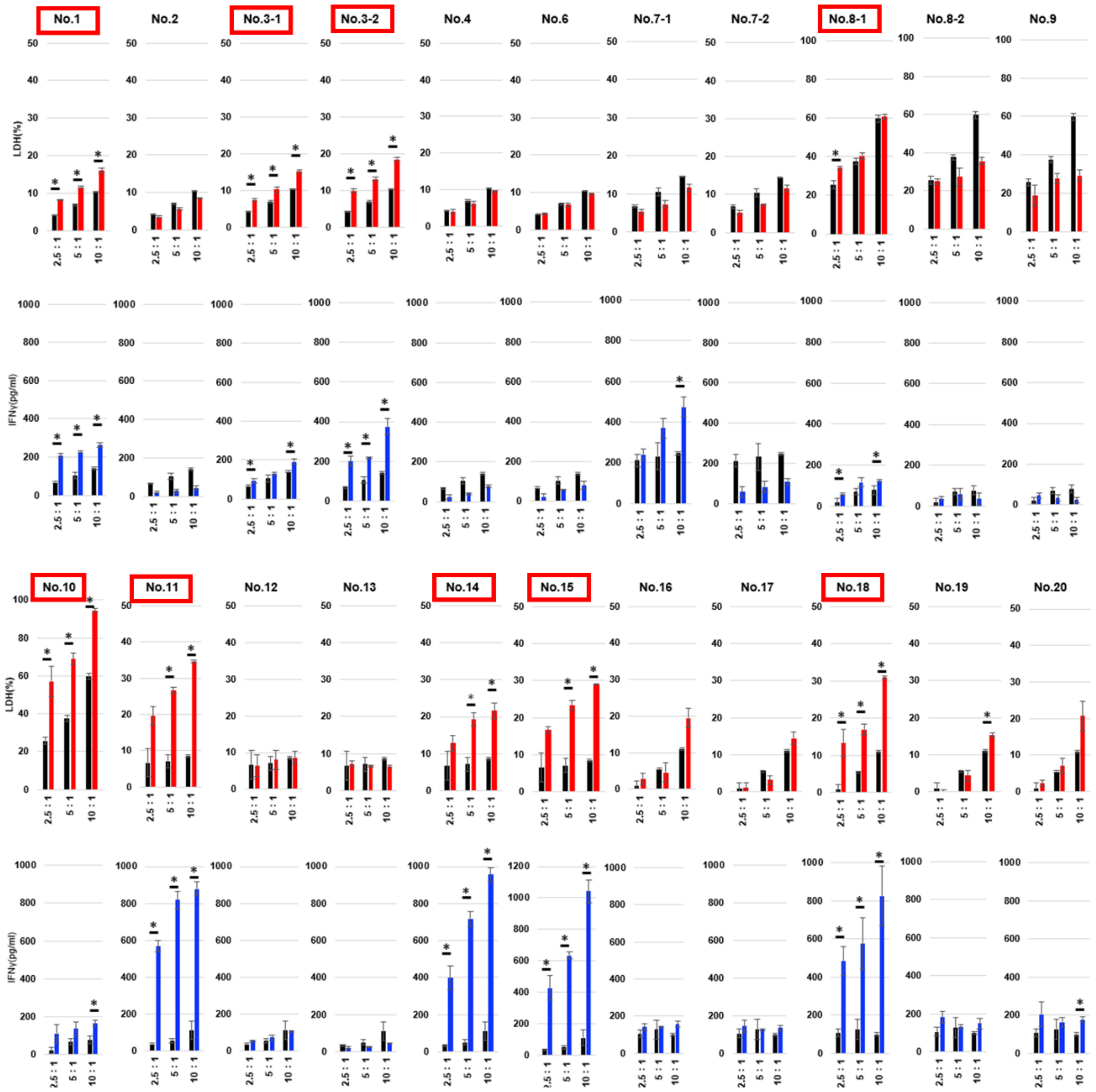

Figure S6

B KID002

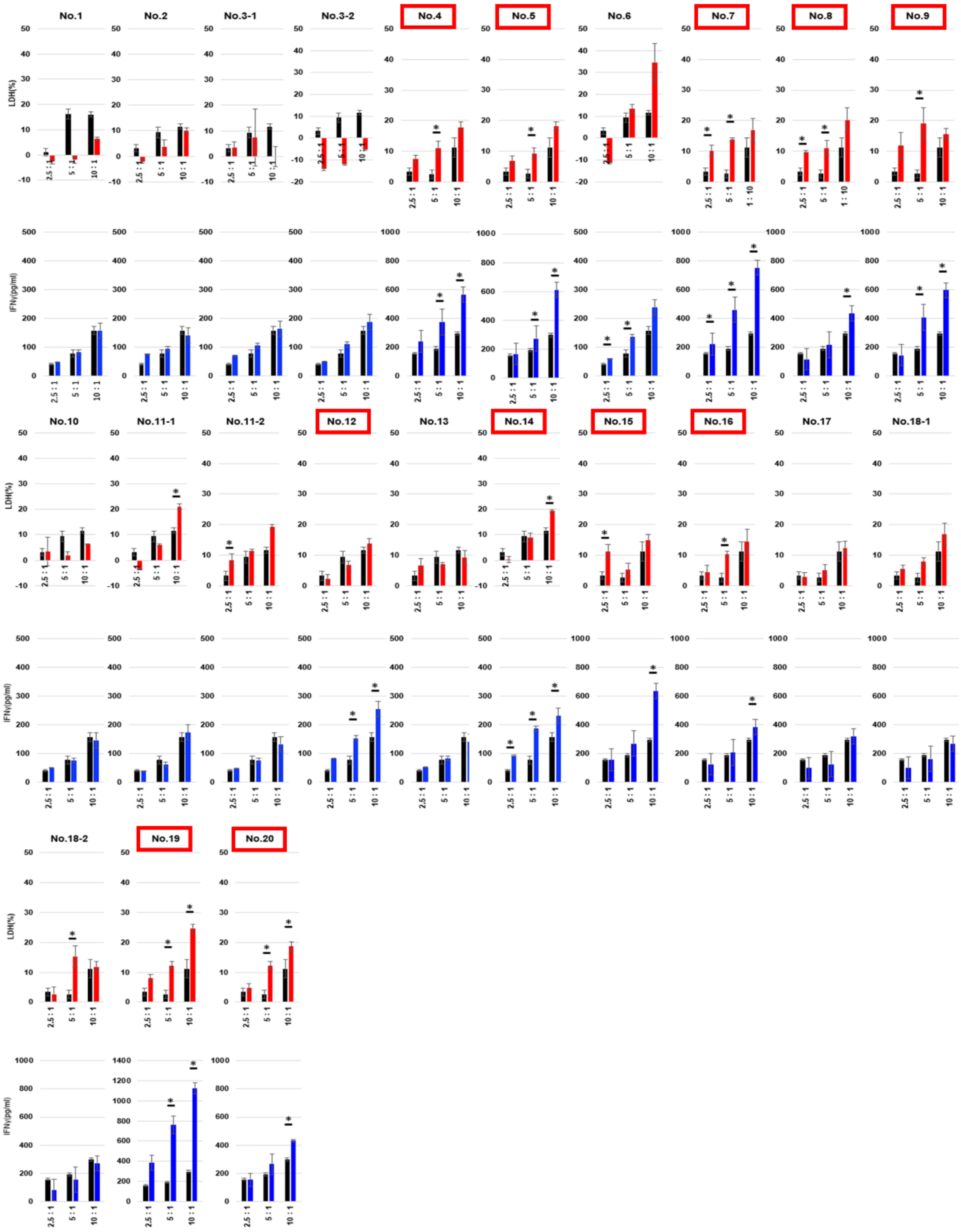

Figure S6

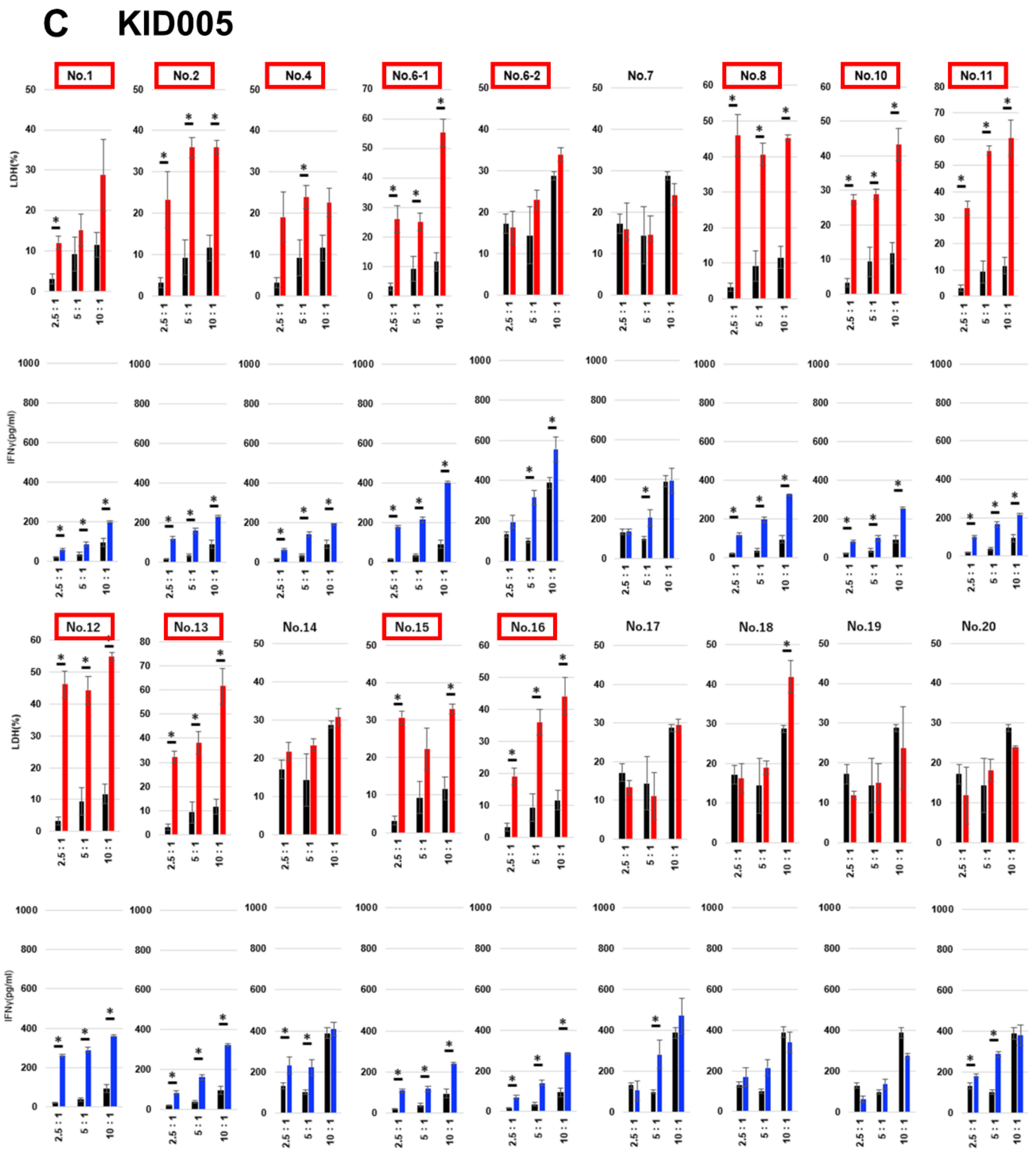
C KID005
No.1
No.2
No.4
No.6-1
No.6-2
No.7
No.8
No.10
No.11
No.12
No.13
No.14
No.15
No.16
No.17
No.18
No.19
No.20
LDH(%)
IFNγ(pg/ml)
2.5 : 1
5 : 1
10 : 1

Figure S6

D KID007

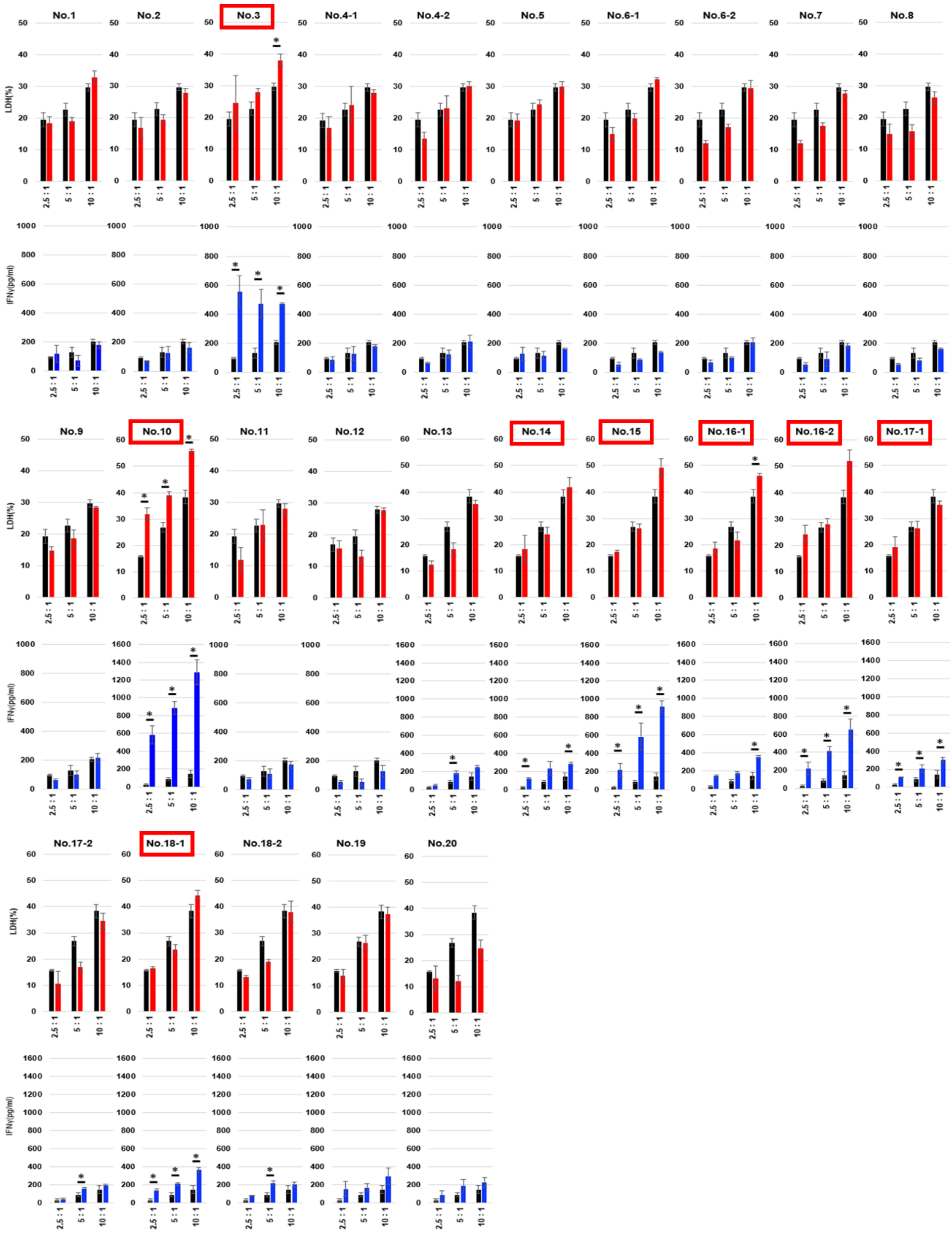

Figure S6

E KID010

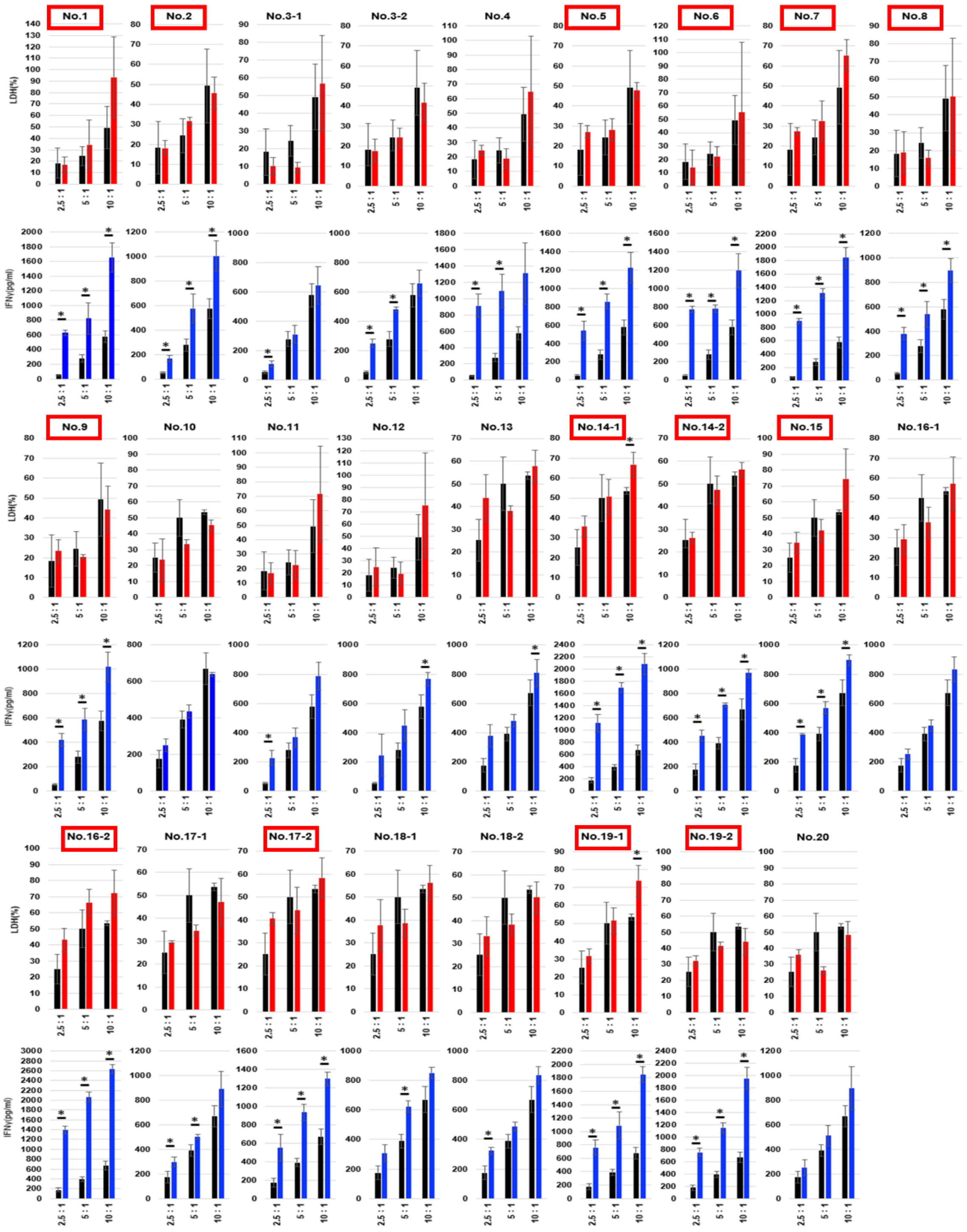

Figure S6. Reactivity of TCR-T cells against autologous tumor-derived organoids
PBMCs from healthy donors were transfected with TCR-expressing vectors derived from the top 20 most abundant TCR clonotypes in five ccRCC patients: (A) KID001, (B) KID002, (C) KID005, (D) KID007, and (E) KID010. The resulting TCR-T cells were co-cultured with autologous tumor-derived organoids, and anti-tumor reactivity was assessed by LDH release assays and IFN-γ secretion measured by ELISA. Bar graphs show LDH release (red) and IFN-γ levels (blue) for each sample. PBMCs transduced with EGFP-containing expression vectors without TCR constructs served as controls (black bars). *P* values were determined using the Student's t test compared with controls. $^*p < 0.05$. Reactivity was assigned when, at an E:T ratio of 10:1, either the LDH release assay or the IFN-γ ELISA showed a statistically significant difference from the control sample, and when at least two data points showed statistically significant differences across all tested conditions. Reactive clonotypes are highlighted in red.

Figure S7

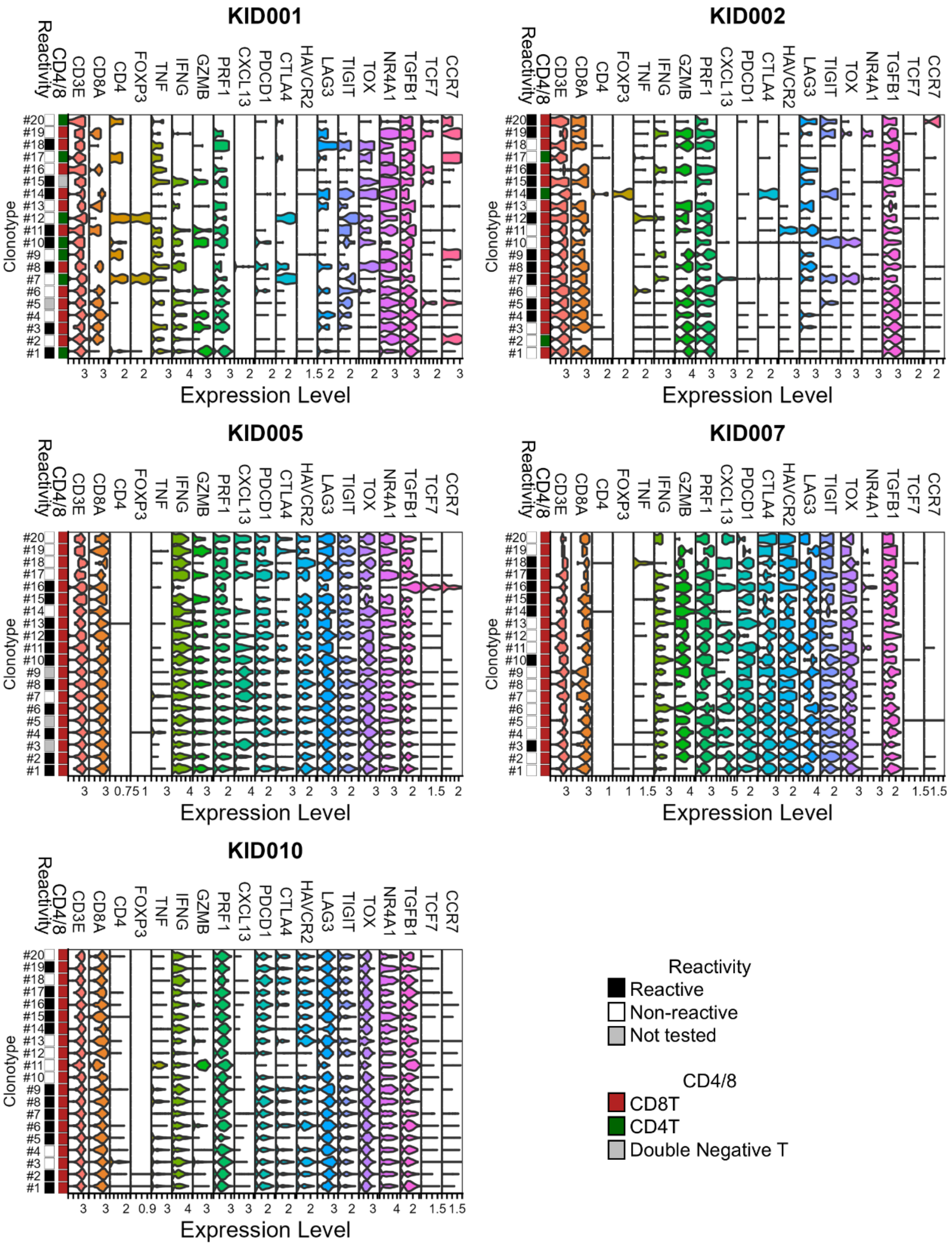
KID001
KID002
KID005
KID007
KID010
Reactivity
CD4/8
CD3E
CD8A
CD4
FOXP3
TNF
IFNG
GZMB
PRF1
CXCL13
PDCD1
CTLA4
HAVCR2
LAG3
TIGIT
TOX
NR4A1
TGFB1
TCF7
CCR7
Clonotype
Expression Level
Reactivity
Reactive
Non-reactive
Not tested
CD4/8
CD8T
CD4T
Double Negative T

Figure S7. Gene expression profiles of organoid-reactive and -non-reactive T cells.

Gene expression profiles of T cell populations expressing the top 20 most abundant TCR clonotypes. Reactivity of each clonotype to organoid cultures is indicated by color bars.

Figure S8

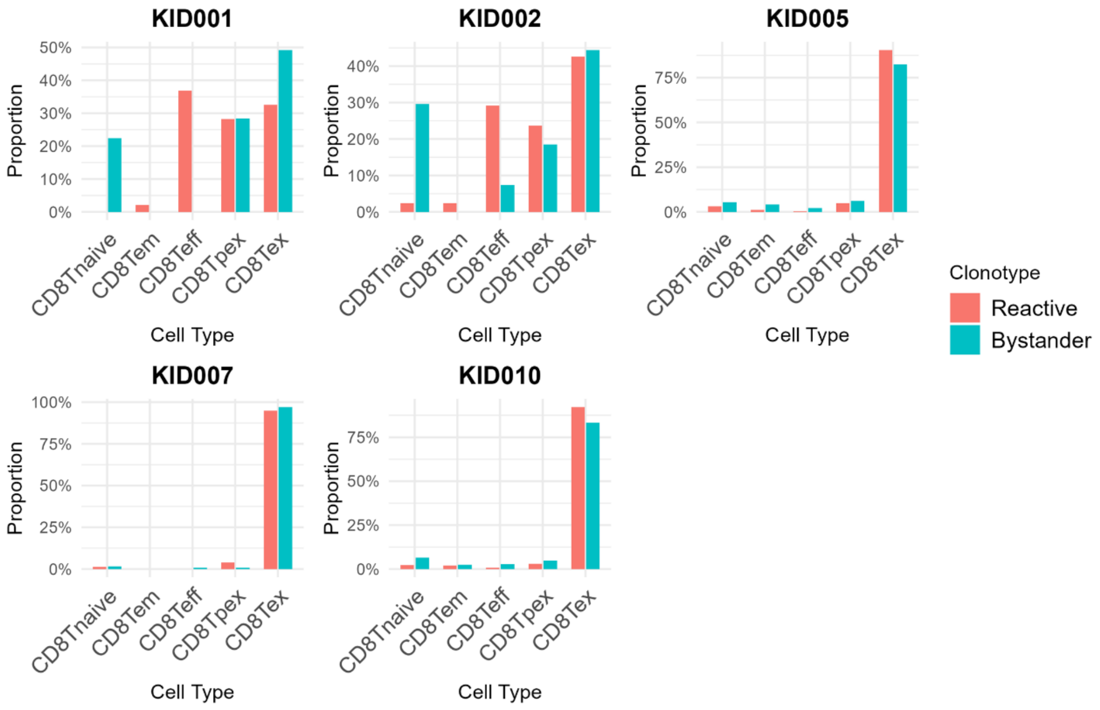
KID001
KID002
KID005
KID007
KID010
Proportion
Cell Type
CD8Tnaive
CD8Tem
CD8Teff
CD8Tpex
CD8Tex
0%
10%
20%
30%
40%
50%
25%
75%
100%
Clonotype
Reactive
Bystander

Figure S8. Cell Type distribution of organoid-reactive clonotypes and bystander clonotypes.

The distribution of $CD8^+$ naïve T cells, $CD8^+$ effector memory T cells, $CD8^+$ effector T cells, $CD8^+$ progenitor exhausted T cells, and $CD8^+$ exhausted T cells was compared between organoid-reactive T cells and bystander T cells.
